# Cleavage of [Pd_2_(PP)_2_(*μ*-Cl)_2_][BArF_24_]_2_ (PP = Bis(phosphino)ferrocene, BArF_24_ = Tetrakis(3,5-bis(trifluoromethyl)phenyl)borate) with Monodentate Phosphines

**DOI:** 10.3390/molecules29092047

**Published:** 2024-04-29

**Authors:** Ian S. Leiby, Virginia Parparcén, Natalya Ding, Klara J. Kunz, Sadie A. Wolfarth, Jeremiah E. Stevens, Chip Nataro

**Affiliations:** 1 Department of Chemistry, Lafayette College, Easton, PA 18045, USA; 2 Department of Chemistry and Biochemistry, The Ohio State University, Columbus, OH 43210, USA

**Keywords:** synthesis, X-ray crystallography, cyclic voltammetry, phosphine, dimer

## Abstract

The addition of Na[BArF_24_] (BArF_24_ = tetrakis(3,5-bis(trifluoromethyl)phenyl)borate) to [Pd(PP)Cl_2_] (PP = 1,1′-bis(phosphino)ferrocene ligands) compounds results in the loss of a chloride ligand and the formation of the dimeric species [Pd_2_(PP)_2_(*μ*-Cl)_2_][BArF_24_]_2_. In most cases, the addition of a monodentate phosphine, PR_3_, to these dimeric species leads to cleaving of the dimer and formation of [Pd(PP)(PR_3_)Cl][BArF_24_]. While these reactions are readily observed via a significant color change, the ^31^P{^1^H} NMR spectra offer more significant support, as the singlet for the dimer is replaced with three doublets of doublets. The reaction seems to take place for a wide range of PR_3_ ligands, although there do appear to be steric limitations to the reaction. The compounds were thoroughly characterized by NMR, and X-ray crystal structures of several of the compounds were obtained. In addition, the ferrocenyl backbone of the 1,1′-bis(phosphino)ferrocene ligands provides an opportunity to examine the oxidative electrochemistry of these compounds. In general, the potential at which oxidations of these compounds occurs shows a dependence on the phosphine substituents.

## 1. Introduction

Palladium(II) compounds with the general formula [Pd(PP)Cl_2_] (PP = two monodentate phosphines or a bidentate phosphine) are commonly used in a wide variety of catalytic applications [1]. When it comes to stoichiometric reactions, one of the more common is the abstraction of a chloride ligand, typically resulting in the formation of a dicationic dimer with the general formula [Pd_2_(PP)_2_(*μ*-Cl)_2_][X]_2_. Numerous reagents have been employed to form these dimers, including BF_3_ [2,3,4], alkali metal salts [5,6,7], [Cu(MeCN)_4_][ClO_4_] [8], silver salts [3,6,9,10,11,12,13,14,15,16,17,18,19,20,21,22,23], thallium salts [24,25], and [Et_3_O][BF_4_] [19]. There have also been several reports in which these dimers form over time in solutions of related species [26,27,28]. While there are many possible routes to these dimers, there are additional factors in their formation. The choice of solvent is significant, as the addition of AgX (X = BF_4_^−^ or ClO_4_^−^) to [Pd(dppe)Cl_2_] in pyridine, dimethylformamide, or dimethylsulfoxide leads to the formation of the solvent-bound species [Pd(dppe)(S)Cl][X] (S = py, dmf or dmso), while the same reactions in acetone, methanol, acetonitrile, tetrahydrofuran, and benzene gave [Pd_2_(dppe)_2_(*μ*-Cl)_2_][X]_2_ [9,29]. Both the solvent and the anion influence the relative stability of the dimer with respect to formation of monometallic compounds for [Pd_2_(bicyclo [3.2.0]heptanyl diphosphinite)_2_(*μ*-Cl)_2_]X_2_ [6]. The ligands also played a role in this reactivity, as the related [Pd(Ph_2_ECH_2_CH_2_E′Ph_2_)Cl_2_] (E = P, E′ = As or E = E′ = As) reacted with AgX to give dimers in all of the aforementioned solvents [29].

Although dimer formation has been reported using a variety of different reagents, there have been surprisingly few studies examining the reactivity of these dimers. The majority of these reactions have focused on the addition of L-type ligands that replace the L-type interaction of a bridging chloride [30]. The first reported study showed that the phosphine can have a significant impact on the reactivity, as there was no reaction between CO and [Pd_2_(PPh_3_)_4_(*μ*-Cl)_2_][BF_4_]_2_, but CO cleaved the analogous PEt_3_ dimer to give [Pd(PEt_3_)_2_(CO)Cl][BF_4_] [2,3]. A later study determined that there was no reaction between CO and [Pd_2_(dppe)_2_(*μ*-Cl)_2_][ClO_4_]_2_, but the addition of PPh_3_ to [Pd_2_(dppe)_2_(*μ*-Cl)_2_][ClO_4_]_2_ resulted in breaking the dimer and formation of [Pd(dppe)(PPh_3_)Cl][ClO_4_] [29]. While not prepared directly from [Pd_2_(PEt_3_)_4_(*μ*-Cl)_2_][BF_4_]_2_, the addition of PR_3_ (R = Et or Ph) to [Pd(PEt_3_)_2_Cl_2_] in the presence of Na[BPh_4_] to give [Pd(PEt_3_)_2_(PR_3_)Cl][BPh_4_] suggests that [Pd_2_(PEt_3_)_4_(*μ*-Cl)_2_][BF_4_]_2_ could be cleaved by the addition of monodentate phosphines [3]. When [Pd_2_(dppf)_2_(*μ*-Cl)_2_][BF_4_]_2_ (dppf = 1,1′-bis(diphenylphosphino)ferrocene) was dissolved in MeCN, the dimer was cleaved to give [Pd(dppf)(MeCN)Cl][BF_4_] [14]. A similar reaction was observed for [Pd_2_(dppp)_2_(*μ*-Cl)_2_][P(1,2-C_6_H_4_O_2_)_3_]_2_ [7]. The reverse reaction of dimer formation can also be accomplished by adding chloride to a dimer, as shown by the reaction of [Pd(dppo)_2_(*μ*-Cl)_2_][BArF_24_]_2_ (dppo = 1,1′-bis(diphenylphosphino)osmocene and BArF_24_ = tetrakis(3,5-bis(trifluoromethyl)phenyl)borate) with [PPN]Cl (PPN = bis(triphenylphosphine)iminium) [31]. One additional reaction that has been reported is the addition of HSiMe(OEt)_2_ to what is presumed to be [Pd_2_(BINAP)_2_(*μ*-Cl)_2_][OTf]_2_, which results in the formation of [Pd(BINAP)(H)Cl] [20].

Additional stoichiometric reactions of these dimers focused on the substitution of the remaining chloride ligands. The addition of two equivalents of Ag[BF_4_] to [Pd_2_(dppf)_2_(*μ*-Cl)_2_][BF_4_]_2_ results in the removal of the chloride ligands and the formation of [Pd(dppf)(dmf)_2_][BF_4_] if the reaction is performed in DMF or [Pd_2_(dppf)_2_(*μ*-OH)_2_][BF_4_]_2_ if the reaction is performed in MeOH [10]. Interestingly, the combination of [Pd_2_(PPh_3_)_4_(*μ*-Cl)_2_][PF_6_]_2_, [H_3_L][PF_6_]_2_ ([H_3_L][PF_6_]_2_ = 3,5-bis(methylimidazolium-1-ylmethyl]-1H-pyrazole bis(hexafluorophosphate)), and 2.5 equivalents of Ag_2_O does not result in chloride loss, but rather yields [Pd_2_(PPh_3_)_2_(*μ*-L)_2_Cl_2_)][PF_6_]_4_ [19]. When two equivalents of Tl[acac] were added to [Pd_2_((1R,2R)-(PPh_2_NH)_2_C_6_H_10_)(*μ*-Cl)_2_][ClO_4_]_2_, the monometallic [Pd((1R,2R)-(PPh_2_NH)_2_C_6_H_10_)(acac)][ClO_4_] forms, whereas with two equivalents of Ag[OAc], the product remains a dimer, [Pd_2_((1R,2R)-(PPh_2_NH)_2_C_6_H_10_)_2_(*μ*-OAc)_2_][ClO_4_] [15]_._ Finally, the reaction of [Pd_2_(PPh_3_)_4_(*μ*-Cl)_2_][BF_4_]_2_ with sodium propargylglycinate yields [Pd(PPh_3_)_2_(propargylglycinate)][BF_4_] [32].

There have also been limited studies of the catalytic activity of these dimeric species. For the isomerization of 1-octene, the dimeric species [Pd_2_(PP)_2_(*μ*-Cl)_2_][ClO_4_]_2_ (PP = 2 PMePh_2_ or dppe) were found to possess inferior catalytic activity compared to the corresponding [Pd(PP)(acetone)_2_][ClO_4_]_2_ compounds [33]. That study also found that the same dimers were generally poorer catalysts than the monometallic species for the isomerization and hydrogenation of 1-octene under varying pressures of hydrogen. Finally, the dimer [Pd_2_(dppe)_2_(*μ*-Cl)_2_][BF_4_]_2_ was found to be inactive as a polymerization catalyst, whereas [Pd(dppe)(MeCN)_2_][BF_4_]_2_ was an active catalyst for alkene polymerization and the co-polymerization of alkenes and carbon monoxide [26]. As these dimeric species generally show inferior catalytic activity and can readily form in the presence of species that can be present in catalytic mixtures, for example alkali metal salts [5,6,7], further examination of their reactivity is warranted.

**Figure 1:**
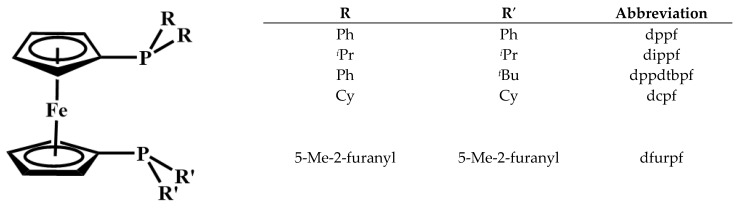
1,1′-Bis(phosphino)ferrocene ligands employed in this study.

**Figure 2:**
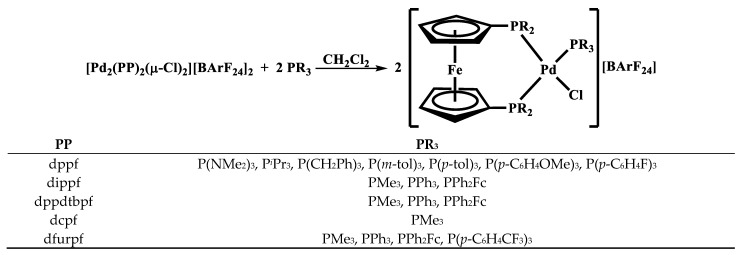
Synthesis of [Pd(PP)(PR_3_)Cl][BArF_24_] compounds.

Previous work in this laboratory has focused on the synthesis and reactivity of similar dimeric species with bis(phosphino)metallocene ligands (Figure 1). The first reported dimer of this type was [Pd_2_(dppf)_2_(*μ*-Cl)_2_][PF_6_]_2_ [34,35]. Related compounds with a variety of different substituents on phosphorous were prepared by the addition of Na[BArF_24_] to [Pd(PP)Cl_2_] (PP = dppf, dippf, dcpf, and dppdtbpf) [36]. Further work examined dimers with the ruthenium and osmium analogs of dppf [31]. Most recently, the reactivity of [Pd_2_(dppf)_2_(*μ*-Cl)_2_][BArF_24_]_2_ with three monodentate phosphines to yield [Pd(dppf)(PR_3_)Cl][BArF_24_] was reported [37]. Herein, additional reactions of dimeric species containing 1,1′-bis(phosphino)ferrocene ligands with monodentate phosphines are reported (Figure 2). The new compounds were all characterized by multinuclear NMR spectroscopy and cyclic voltammetry, and, in several cases, X-ray crystal structures were obtained.

## 2. Results and Discussion

The synthesis of [Pd_2_(dfurpf)_2_(*μ*-Cl)_2_][BArF_24_]_2_ was performed similarly to that of the related dimers in this study. The addition of Na[BArF_24_] to a solution of [Pd(dfurpf)Cl_2_] in CH_2_Cl_2_ resulted in an immediate color change from orange to brown–green. A downfield shift in the ^31^P{^1^H} NMR signal, typically seen in dimer formation, was noted [36]. The electrochemistry of [Pd_2_(dfurpf)_2_(*μ*-Cl)_2_][BArF_24_]_2_ was examined, and the oxidative electrochemistry displays a single reversible wave at 0.74 V vs. FcH^0/+^. Oxidation of the neutral dichloride, [Pd(dfurpf)Cl_2_], occurs at 0.55 V vs. FcH^+^ [38], and the more positive potential found for the oxidation of the dimeric species is consistent with the dimer being a dication. The potential at which oxidation of the dfurpf dimer occurs is similar to the potential for the most positive oxidative wave for the [Pd_2_(PP)_2_(*μ*-Cl)_2_][BArF_24_]_2_ (PP = dppf (0.74 V), dippf (0.70 V), dcpf (0.73 V), or dppdtbpf (0.74 V)) [36]. The similarity of these is somewhat surprising, as the potentials at which the oxidation of the [Pd(PP)Cl_2_] compounds (PP = dppf (0.57 V) [39], dippf (0.43 V) [40], dcpf (0.47 V) [41], and dpptdtbpf (0.51 V) [39]) occur show a greater dependence on the substituents on phosphorus. A single, irreversible wave for the reduction of [Pd_2_(dfurpf)_2_(*μ*-Cl)_2_][BArF_24_]_2_ was observed at −0.73 V vs. FcH^0/+^. The related compounds also displayed a single, irreversible wave, i.e., [Pd_2_(PP)_2_(*μ*-Cl)_2_][BArF_24_]_2_ (PP = dppf (−0.75 V), dippf (−0.95 V), dcpf (−0.82 V), or dppdtbpf (−0.71 V)) [36], which also showed more dependence on the phosphorus substituents than the oxidative wave.

**Figure 3:**
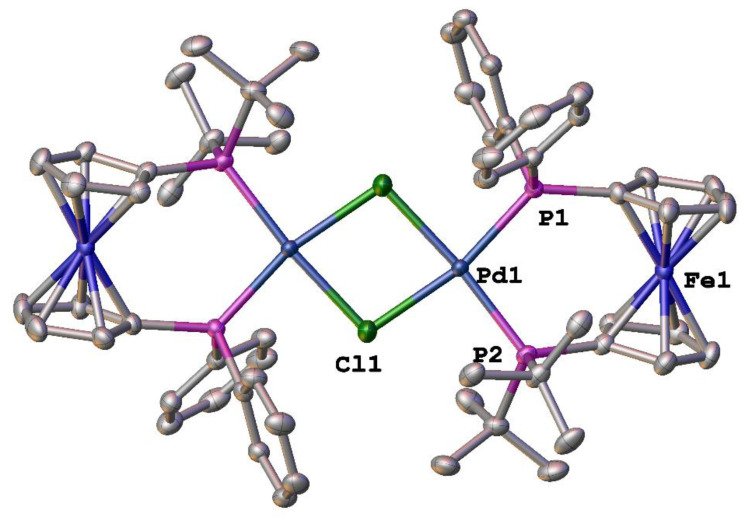
Structure of [Pd_2_(dppdtbpf)_2_(*μ*-Cl)_2_][BArF_24_]_2_. Thermal ellipsoids are drawn at the 50% probability level and the H atoms and two [BArF_24_]^−^ are omitted for clarity.

During the course of this investigation, crystals of [Pd_2_(dppdtbpf)_2_(*μ*-Cl)_2_][BArF_24_]_2_ suitable for X-ray analysis were obtained. While there are potentially two different isomers of [Pd_2_(dppdtbpf)_2_(*μ*-Cl)_2_][BArF_24_]_2_ (*syn*- and *anti*-), the compound adopts the anti-form in the solid state (Figure 3). The solution NMR data suggest that there is a single isomer present [36], presumably the anti-isomer observed in the solid state. However, the possibility that both isomers do exist in solution as either an undetectable amount of one isomer, fast exchange of both isomers, or coincidental signals for both isomers cannot be ruled out. The structural parameters of [Pd_2_(dppdtbpf)_2_(*μ*-Cl)_2_][BArF_24_]_2_ are quite similar to those for [Pd(dppdtbpf)Cl_2_] [42] (Table 1). The percent buried volume (%*V*_bur_) is a calculation used to quantify the steric bulk of various ligands [43], and there is an increase in this parameter for the dppdtbpf ligand in [Pd_2_(dppdtbpf)_2_(*μ*-Cl)_2_][BArF_24_]_2_ as compared to [Pd(dfurpf)Cl_2_]. A similar trend has been noted for the analogous dppf [36], 1,1′-bis(diphenylphosphino)ruthenocene [31] and 1,1′-bis(diphenylphosphino)osmocene [31] compounds. The [Pd_2_(dppdtbpf)_2_(*μ*-Cl)_2_][BArF_24_]_2_ also shows a slight decrease in the P–Pd–P bite angle as compared to [Pd(dppdtbpf)Cl_2_], but the overall geometries of the palladium centers, as indicated by the four-coordinate geometry indices *τ*_4_ [44] and *τ*′_4_ [45], are identical.

**Figure 4:**
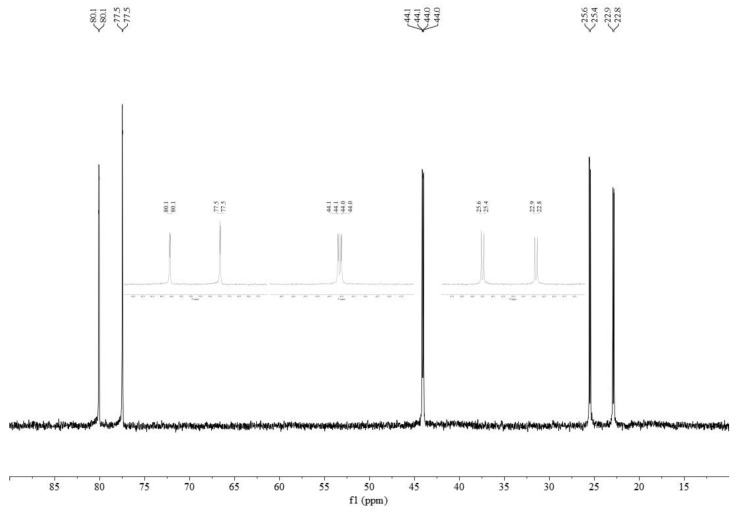
^31^P{^1^H} NMR spectrum of [Pd(dppdtbpf)(PPh_3_)Cl][BArF_24_] in CDCl_2_. Expanded views of each signal are inset.

The addition of two equivalents of a monodentate phosphine to the various [Pd_2_(PP)_2_(*μ*-Cl)_2_][BArF_24_]_2_ dimers typically resulted in an immediate change in the color of the solution from green–brown to orange–red. The single peak in the ^31^P{^1^H} spectra for most of the dimers is replaced by three doublets of doublets, two of which exhibit large coupling constants (approx. 500 Hz) indicative of coupling for *trans*-phosphorus atoms (Table 2). When comparing the same monodentate phosphine, there is an apparent trend in the magnitude of the *trans*-coupling constants with dfurpf > dppf > dippf > dcpf > dppdtbpf. In the case of [Pd_2_(dppdtbpf)_2_(*μ*-Cl)_2_][BArF_24_]_2_, the dimer exhibits two peaks due to the inequivalent phosphorus atoms, but upon the addition of a monodentate phosphine, this changes to three doublets of doublets (Figure 4). The similarity of dppdtbpf to dippf and dcpf in the ranking of *trans*-coupling constants suggests that the monodentate phosphines add *trans*- to the –P*^t^*Bu_2_ group. Similarly, the signal for the –P*^t^*Bu_2_ group in dppdtbpf occurs approximately 30 ppm downfield compared to peak for the –PPh_2_ groups in the dppdtbpf and dppf compounds, and this downfield peak displays coupling to the *trans*-PR_3_ group.

There were two notable exceptions in which no reaction was observed, and in both cases this can most likely be attributed to steric considerations. The first was the addition of P(*o*-tol)_3_ to [Pd_2_(dppf)_2_(*μ*-Cl)_2_][BArF_24_]_2_. The %*V*_bur_ of P(*o*-tolyl)_3_ is 41.4%, which is significantly larger than P(*p*-tol)_3_ (28.2%)[43] and P(*m*-tol)_3_ (28.0%) which was calculated from the reported structure [46]. The second was the addition of PPh_3_ to [Pd_2_(dcpf)_2_(*μ*-Cl)_2_][BArF_24_]_2_. The %*V*_bur_ for the [PdCl_2_(PP)] compounds has been calculated, and dcpf (57.5%) [43] is larger than dippf (56.5%) [43], dppf (55.5%) [43], and dfurpf (53.7%) [47], suggesting that the steric hinderance of the bis(phosphino)ferrocene ligand may prevent a reaction from occurring. The %*V*_bur_ for dppdtbpf in [Pd(dppdtbpf)Cl_2_] has also been calculated (58.2%) [48], and this value suggests that [Pd_2_(dppdtbpf)_2_(*μ*-Cl)_2_][BArF_24_]_2_ may not react with PPh_3_. However, the %*V*_bur_ value for dppdtbpf in these compounds is the exact average of dppf and dtbpf (dtbpf = 1,1′-bis(di*tert*-butylphosphino)ferrocene, 60.9%) [43], and, thus, the inherent asymmetry in dppdtbpf may provide sufficient access to the palladium center to allow for a reaction with PPh_3_.

**Figure 5:**
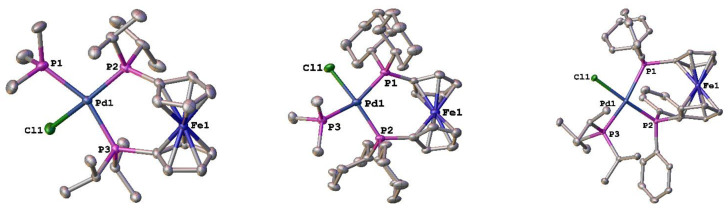
Structures of [Pd(PP)(PR_3_)Cl][BArF_24_] (PP = dippf or dcpf and PR_3_ = PMe_3_; PP = dppf and PR_3_ = P*^i^*Pr_3_). Thermal ellipsoids are drawn at the 50% probability level, and the H atoms, [BArF_24_]^−^, and any solvent molecules are omitted for clarity.

Structures of several of these new compounds with both alkyl and aryl monodentate phosphine ligands were obtained. For the alkylphosphines, the structures of [Pd(PP)(PR_3_)Cl][BArF_24_] (PP = dippf or dcpf and PR_3_ = PMe_3_; PP = dppf and PR_3_ = P*^i^*Pr_3_) (Figure 5) were determined. In comparing the same monodentate phosphine, PMe_3_, some general trends can be noted. For the [Pd(PP)Cl_2_] compounds, the %*V*_bur_ values vary by 2%, with dppf being the smallest, followed by dippf (56.5), and then dcpf (57.5) [43]. In the [Pd(PP)(PMe_3_)Cl][BArF_24_] compounds, the order of the %*V*_bur_ values is dippf (56.6) > dppf (55.1) > dcpf (55.0) (Table 3). Additional structural metrics provide some insight into this discrepancy. The ability of the C_5_ rings in the 1,1′-bis(phosphino)ferrocene ligands to twist (C–Cent–Cent–C) allow for these ligands to adopt a variety of different conformations [49,50]. The twist angle in [Pd(dppf)(PMe_3_)Cl][BArF_24_] is nearly double that of the dippf and dcpf analogues. The bite angles for the dippf and dcpf ligands are within the experimental error of each other and are significantly larger than that of dppf in these compounds. There is also significant distortion at the palladium center, as the palladium in the dppf compound is much closer to the idealized square planar geometry than in the dippf or dcpf analogues. The %*V*_bur_ for the PMe_3_ ligand spans a range of 0.6% in these compounds, suggesting that it is the flexibility of the 1,1′-bis(phosphino)ferrocene ligands that allows for coordination of the monodentate phosphine.

The structure of [Pd(dppf)(P*^i^*Pr_3_)Cl][BArF_24_] allows for comparison of structures in which the bis(phosphino)ferrocene ligand is kept constant while the monodentate phosphine is changed. In comparison to the PMe_3_ analogue, [Pd(dppf)(P*^i^*Pr)Cl][BArF_24_] shows a significant compression (5.8°) of the dppf bite angle. This is accompanied by further distortion from the idealized square planar geometry of palladium. There is also an increase in the deviation from the C_5_ rings being parallel of 0.5° and a decrease in the twist angle of 3.7°. The %*V*_bur_ is significantly smaller compared to similar dppf compounds, as the 52.1% value found for this compound is well outside one standard deviation of the average value of 54.0% found for 61 similar compounds [37].

**Figure 6:**
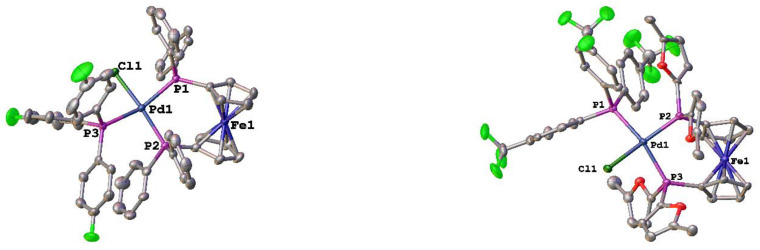
Structures of [Pd(PP)(PR_3_)Cl][BArF_24_] (PP = dppf and PR_3_ = P(*p*-C_6_H_4_F)_3_; PP = dfurpf and PR_3_ = P(*p*-C_6_H_4_CF_3_)_3_). Thermal ellipsoids are drawn at the 50% probability level and the H atoms, [BArF_24_]^-^ and any solvent molecules are omitted for clarity.

In addition to the tri-alkyl phosphines, the structures of two new tri-aryl phosphine compounds were determined (Figure 6). Not surprisingly, there are not tremendous differences between the structures of [Pd(dppf)(PPh_3_)Cl][BArF_24_] and [Pd(dppf)(P(*p*-C_6_H_4_F)_3_)Cl][BArF_24_] (Table 4). The monodentate phosphines were calculated to have the same %*V*_bur_ (27.9). The dppf has a slightly larger %*V*_bur_ in the P(*p*-C_6_H_4_F)_3_ compound (56.1 vs. 55.4), the P–Pd–P bite angle decreases by 0.75°, and there is a slightly larger deviation from the ideal square planar geometry for the palladium. For [Pd(dfurpf)(P(*p*-C_6_H_4_CF_3_)_3_)Cl][BArF_24_], the monodentate phosphine has a slightly larger %*V*_bur_, while the dfurpf ligand has a %*V*_bur_ that is approximately 4% less than that of dppf in [Pd(dppf)(PPh_3_)Cl][BArF_24_]. This is somewhat surprising, as dfurpf only has a 1.5% difference in %*V*_bur_ in the corresponding [Pd(PP)Cl_2_] compounds [47].

The oxidative electrochemistry of the [Pd(PP)(PR_3_)Cl][BArF_24_] compounds was examined in CH_2_Cl_2_. With the exception of the [Pd(PP)(PPh_2_Fc)Cl][BArF_24_] compounds, a single, reversible wave at potentials more positive than that of the corresponding [Pd(PP)Cl_2_] compounds is observed. Variations in the 1,1′-bis(phosphino)ferrocene ligands have a significant impact on the potential at which oxidation of the [Pd(PP)Cl_2_] compounds occurs, and a similar trend is noted for the [Pd(PP)(PR_3_)Cl][BArF_24_] compounds, wherein alkyl substituents on the 1,1′-bis(phosphino)ferrocene ligands lead to less positive potentials (Table 5). Varying the PR_3_ group has a minimal effect on the potentials at which oxidations occur, with the various [Pd(PP)(PR_3_)Cl][BArF_24_] compounds undergoing oxidation at similar potentials for PMe_3_ and PPh_3_ derivatives.

**Figure 7:**
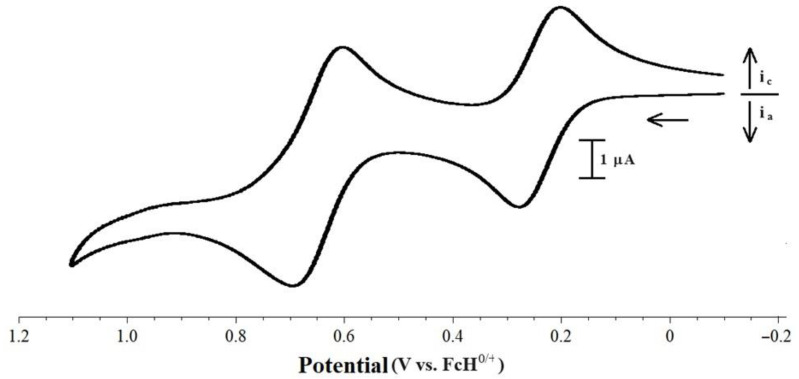
CV scan of 1.0 mM [Pd(dfurpf)(PPh_2_Fc)Cl][BArF_24_] with 0.1 M [NBu_4_][PF_6_] as the supporting electrolyte, measured at 100 mV s^−1^.

Two waves were observed for the [Pd(PP)(PPh_2_Fc)Cl][BArF_24_] compounds, as both iron centers undergo oxidation at significantly different potentials (Figure 7). The separation of the peaks classifies all of these compounds as Class I Robin–Day mixed valence systems with *K_c_* < 10^2^ [51,52]. There was no change in the reversibility of the waves at approximately 0.25 V vs. FcH^0/+^ when the switching potential was set at 0.55 V. The oxidation at approximately 0.25 V is likely due to the oxidation of the iron center of the PPh_2_Fc ligand. Free PPh_2_Fc undergoes oxidation at 0.06 V vs. FcH^0/+^ [53], which is significantly less positive than the potential, 0.23 V vs. FcH^0/+^ [54], at which the oxidation of free dppf occurs. Coordination of these ligands leads to a further positive shift in the potentials at which oxidation occurs. For [Pd(dppf)Cl_2_], oxidization occurs at 0.62 V vs. FcH^0/+^ [55]. In [Pd(PPh_2_Fc)_2_Cl_2_] the presence of the two iron centers results in two oxidative waves, the first at 0.14 V vs. FcH^0/+^ and the second at 0.26 V [56]. Based on these compounds, it is reasonable to suggest that for the [Pd(PP)(PPh_2_Fc)Cl][BArF_24_] compounds in this study, the oxidation at approximately 0.26 V is likely due to the iron of the PPh_2_Fc ligand, while the wave at approximately 0.65 V is likely due to oxidation of the iron of the PP ligands. The similarity of the potential for the second oxidation in the [Pd(PP)(PPh_2_Fc)Cl][BArF_24_] compounds to that of the lone oxidation for other [Pd(PP)(PR_3_)Cl][BArF_24_] compounds is also supportive of this assignment. However, it is somewhat surprising that the second oxidation in these compounds does not occur at even more positive potentials, as the oxidation of the second iron center in [Pd(PPh_2_Fc)_2_Cl_2_] occurs 0.12 V more positive than the first wave [56].

**Figure 8:**
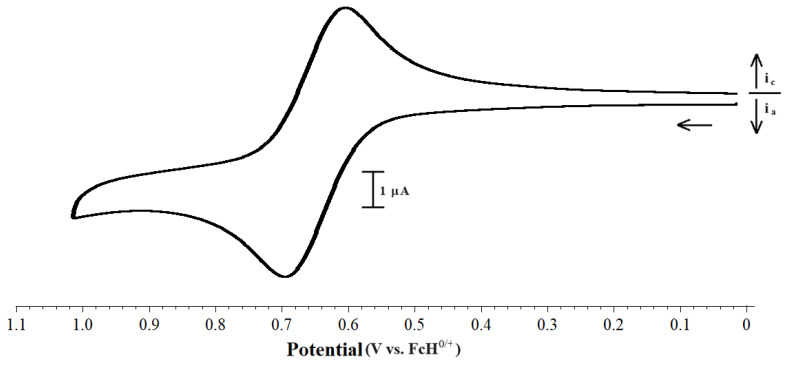
CV scan of 1.0 mM [Pd(dppf)(P^i^Pr_3_)Cl][BArF_24_] with 0.1 M [NBu_4_][PF_6_] as the supporting electrolyte, measured at 100 mV s^−1^.

The electrochemistry of several other [Pd(PP)(PR_3_)Cl][BArF_24_] compounds, primarily with dppf as the bis(phosphino)ferrocene ligand, was also examined. These compounds display a single reversible wave (Figure 8). The potentials at which the oxidation of these compounds occur are quite narrow for the variety of phosphines employed in this study, indicating that the iron center is somewhat insulated from the effects by small changes in the electron richness and the steric properties of the monodentate phosphine (Table 6).

## 3. Materials and Methods

### 3.1. General Procedures

All experiments were conducted under an argon atmosphere, employing standard Schlenk techniques, unless otherwise specified. Reagents were used in their as-received form from the manufacturer, except as otherwise indicated. Bis(acetonitrile) palladium dichloride [PdCl_2_(MeCN)_2_], triphenylphosphine (PPh_3_), 1.0 M trimethylphosphine (PMe_3_) in THF, and tetrabutylammonium hexafluorophosphate [NBu_4_][PF_6_] were purchased from Aldrich. The 1,1′-bis(phosphino)ferrocene ligands, ferrocene (FcH), tri-isopropylphoshine, tribenzylphosphine, tri(*o*-tol)phosphine, tri(*m*-tol)phosphine, tri(*p*-tol)phosphine, tri(*p*-methoxyphenyl)phosphine, tri(*p-*fluorophenyl)phosphine, and tris(*p*-trifluoromethylphenyl)phosphine were purchased from Strem. Diphenylphosphinoferrocene (PPh_2_Fc) [57], Na[BArF_24_] [58], and the dimers [Pd_2_(PP)_2_(*μ*-Cl)_2_][BArF_24_]_2_ (PP = dppf, dippf, dcpf, or dppdtbpf) [36] were synthesized following the literature methods. Solvents were obtained from Fisher Scientific. The [NBu_4_][PF_6_] was dried at 100 °C under vacuum prior to use. FcH was sublimed prior to use. The purification of methylene chloride (CH_2_Cl_2_) and diethyl ether (Et_2_O) was performed using a Solv-Tek purification system [59]. The ^31^P{^1^H}, ^13^C{^1^H}, ^19^F {^1^H}, and ^1^H NMR spectra were recorded in the reported solvents using a Bruker Avance III HD 400 FT-NMR spectrometer. The ^1^H and ^13^C{^1^H} NMR spectra were referenced to residual solvent peaks. The ^31^P{^1^H} NMR spectra were referenced using external 85% H_3_PO_4_, and the ^19^F{^1^H} NMR spectra were referenced to external C_6_F_6_. Elemental analysis was carried out by Midwest Microlab.

### 3.2. Synthetic Procedures and Characterization

#### 3.2.1. Synthesis of [Pd_2_(dfurpf)_2_(*μ*-Cl)_2_][BArF_24_]_2_

Equal molar amounts of [Pd(dfurpf)Cl_2_] (0.2100 g, 0.281 mmol) and Na[BArF_24_] (0.2437 g, 0.281 mmol) were placed in a flask equipped with a stir bar. After placing the solids under an atmosphere of argon, CH_2_Cl_2_ (15 mL) was added to the flask. The solution quickly changed color from orange to brown–green, and the reaction was stirred for 15 min. The reaction mixture was then filtered through celite, and the volume of the resulting filtrate was reduced to approximately 5 mL. The solution was then layered with Et_2_O (20 mL), and the solution was placed in a −10 °C freezer for approximately 3 d, during which time brown–green crystals formed. The resulting solution was filtered, and the solid was washed with Et_2_O (5 mL) and dried under vacuum. The product was obtained as a green–brown solid (0.6075 g, 96% yield). ^1^H NMR (CD_2_Cl_2_): δ 7.64 (br s, 8H, BArF_24_), 7.48 (br s, 4H, BArF_24_), 6.75 (br s, 4H, -*furanyl*), 6.09 (br s, 4H, -*furanyl*), 4.55 (AA′XX′, 4H, -C_5_*H*_4_), 4.40 (AA′XX′, 4H, -C_5_*H*_4_), 2.22 (s, 12H, -*Me*). ^13^C{^1^H} NMR (CD_2_Cl_2_): δ 161.9 (q, ^1^*J*_B-C_ = 49.9 Hz, no DEPT, BArF_24_), 161.9 (s, no DEPT, -*furanyl*), 137.9 (d, ^1^*J*_P-C_ = 103.2 Hz, no DEPT, -*furanyl*), 134.9 (m, DEPT +, BArF_24_), 129.0 (m, no DEPT, BArF_24_), 127.3 (s, DEPT +, -*furanyl*), 124.7 (q, ^1^*J*_F-C_ = 272.4 Hz, no DEPT, BArF_24_), 117.6 (m, DEPT +, BArF_24_), 109.0 (s, DEPT +, -*furanyl*), 77.3 (s, DEPT +, -*C*_5_H_4_), 76.0 (s, DEPT +, -*C*_5_H_4_), 68.3 (d, ^1^*J*_P-C_ = 84.4 Hz, no DEPT, -*C*_5_H_4_), 14.0 (s, DEPT +, -*Me*). ^19^F{^1^H} NMR (CD_2_Cl_2_): δ −62.8 (s). ^31^P{^1^H} NMR (CD_2_Cl_2_): δ 2.7 (s). *Anal*. Calc. for C_124_H_80_B_2_Cl_2_F_48_Fe_28_P_4_Pd_2_: C, 32.28; H, 1.80. Found: C, 32.02; H, 1.64%.

#### 3.2.2. Synthesis of [Pd(PP)(PR_3_)Cl][BArF_24_] Compounds

Approximately 100 mg of the appropriate [Pd_2_(PP)_2_(*μ*-Cl)_2_][BArF_24_]_2_ complex was placed in a flask equipped with a stir bar. For air-stable phosphines, two molar equivalents of the desired phosphine were added to the flask, which was then degassed and filled with argon before the CH_2_Cl_2_ (10 mL) was added. For air-sensitive phosphines, the flask was degassed, filled with argon, and then CH_2_Cl_2_ (10 mL), followed by two molar equivalents of the appropriate phosphine, were added. The reaction was then stirred overnight. The volume of the solution was reduced to approximately 3 mL and then layered with Et_2_O and placed in a −10 °C freezer for 48 h. The resulting solid was collected by filtration, washed with Et_2_O, and dried under vacuum.

##### [Pd(dippf)(PMe_3_)Cl][BArF_24_]

The product was isolated as an orange solid in 70% yield. ^1^H NMR (CD_2_Cl_2_) δ: 7.64 (br s, 8H, BArF_24_), 7.48 (br s, 4H, BArF_24_), 4.50 (m, 8H, -C_5_*H*_4_), 2.96 (m, 2H, -C*H*Me_2_), 2.30 (m, 2H, -C*H*Me_2_), 1.65 (d, ^2^*J*_P-H_ = 10.4 Hz, 9H, -*Me*), 1.12 (m, 18H, -CH*Me*_2_). ^13^C{^1^H} NMR (CD_2_Cl_2_): δ 161.7 (q, ^1^*J*_B-C_ = 49.9 Hz, no DEPT, BArF_24_), 134.8 (m, DEPT +, BArF_24_), 128.8 (m, no DEPT, BArF_24_), 124.6 (q, ^1^*J*_F-C_ = 272.3 Hz, no DEPT, BArF_24_), 117.5 (m, DEPT +, BArF_24_), 74.5 (d, ^2^*J*_P-C_ = 7.5 Hz, DEPT +, -*C_5_*H_4_), 74.0 (d, ^2^*J*_P-C_ = 6.6 Hz, DEPT +, -*C_5_*H_4_), 73.4 (d, ^3^*J*_P-C_ = 5.2 Hz, DEPT +, -*C_5_*H_4_), 73.3 (d, ^3^*J*_P-C_ = 6.4 Hz, DEPT +, -*C_5_*H_4_), 72.4 (m, no DEPT, -*C*_5_H_4_), 28.0 (d, ^1^*J*_P-C_ = 26.2 Hz, -*C*HMe_2_), 19.6 (s, DEPT +, -CH*Me*_2_), 17.1 (d, ^1^*J*_P-C_ = 30.2 Hz, DEPT +, -P*C*H_3_). ^19^F{^1^H} NMR (CD_2_Cl_2_): δ −62.8 (s). ^31^P{^1^H} NMR (CD_2_Cl_2_): δ 67.7 (dd, ^2^*J*_P-P_ = 31.2 and 12.0 Hz, -*P*^i^Pr_2_), 57.9 (dd, ^2^*J*_P-P_ = 446.1 and 12.0, -P^i^Pr_2_), −11.0 (dd, ^2^*J*_P-P_ = 446.1 and 31.2 Hz, -*P*Me_3_). *Anal*. Calc. for C_47_H_57_BClF_24_FeP_3_Pd: C, 44.34; H, 4.51. Found: C, 44.22; H, 4.44%.

##### [Pd(dippf)(PPh_3_)Cl][BArF_24_]

The product was isolated as an orange solid in 58% yield. ^1^H NMR (CD_2_Cl_2_) δ: 7.64 (br s, 11H, -*Ph* and BArF_24_), 7.54–7.38 (m, 13H, -*Ph* and BArF_24_), 7.23 (m, 3H, -*Ph*), 4.57 (AA′XX′, 2H, -C_5_*H*_4_), 4.52 (AA′XX′, 2H, -C_5_*H*_4_), 4.50 (AA′XX′, 2H, -C_5_*H*_4_), 4.48 (AA′XX′, 2H, -C_5_*H*_4_), 2.97 (m, 2H, -C*H*Me_2_), 2.49 (m, 2H, -C*H*Me_2_), 1.18 (m, 18H, -CH*Me*_2_). ^13^C{^1^H} NMR (CD_2_Cl_2_): δ 161.9 (q, ^1^*J*_B-C_ = 49.8 Hz, no DEPT, BArF_24_), 135.4 (d, ^3^*J*_P-C_ = 10.1 Hz, DEPT +m -*Ph*), 134.9 (m, DEPT +, BArF_24_), 131.9 (d, ^4^*J*_P-C_ = 3.0 Hz, DEPT +, -*Ph*), 131.0 (d, ^1^*J*_P-C_ = 47.6 Hz, no DEPT, -*Ph*), 129.1 (m, no DEPT, BArF_24_), 128.9 (d, ^2^*J*_P-C_ = 10.4 Hz, DEPT +, -*Ph*), 124.7 (q, ^1^*J*_F-C_ = 272.5 Hz, no DEPT, BArF_24_), 117.6 (m, DEPT +, BArF_24_), 74.7 (d, ^2^*J*_P-C_ = 7.4 Hz, DEPT +, -*C_5_*H_4_), 74.2 (d, ^2^*J*_P-C_ = 6.7 Hz, DEPT +, -*C_5_*H_4_), 73.6 (d, ^3^*J*_P-C_ = 5.6 Hz, DEPT +, -*C_5_*H_4_), 73.3 (d, ^3^*J*_P-C_ = 6.6 Hz, DEPT +, -*C_5_*H_4_), 72.6 (m, no DEPT, -*C*_5_H_4_), 28.2 (d, ^1^*J*_P-C_ = 26.5 Hz, DEPT +, -*C*HMe_2_), 19.8 (s, DEPT +, -CH*Me*_2_). ^19^F{^1^H} NMR (CD_2_Cl_2_): δ −62.8 (s). ^31^P{^1^H} NMR (CD_2_Cl_2_): δ 65.5 (dd, ^2^*J*_P-P_ = 24.0 and 14.1 Hz, -*P*^i^Pr_2_), 62.5 (dd, ^2^*J*_P-P_ = 428.1 and 14.1, -*P*^i^Pr_2_), 23.7 (dd, ^2^*J*_P-P_ = 428.1 and 24.0 Hz, -*P*Ph_3_). *Anal*. Calc. for C_72_H_63_BClF_24_FeP_3_Pd: C, 54.76; H, 4.02. Found: C, 54.87; H, 4.29%.

##### [Pd(dippf)(PPh_2_Fc)Cl][BArF_24_]

The product was isolated as a red–purple solid in 59% yield. ^1^H NMR (CD_2_Cl_2_) δ: 7.83 (br s, 4H, -*Ph*), 7.65 (br s, 8H, BArF_24_), 7.46 (m, 6H, -*Ph* and BArF_24_), 7.27 (m, 4H, -*Ph*), 4.57 (AA′XX′, 2H, -C_5_*H*_4_), 4.52 (AA′XX′, 2H, -C_5_*H*_4_), 4.48 (AA′XX′, 2H, -C_5_*H*_4_), 4.45 (AA′XX′, 2H, -C_5_*H*_4_), 4.41 (AA′XX′, 2H, -C_5_*H*_4_), 3.97 (AA′XX′, 2H, -C_5_*H*_4_), 3.94 (s, 5H, *Cp*), 2.93 (m, 4H, -C*H*Me_2_), 1.18 (m, 18H, -CH*Me*_2_). ^13^C{^1^H} NMR (CD_2_Cl_2_): δ 161.9 (q, ^1^*J*_B-C_ = 49.9 Hz, no DEPT, BArF_24_), 134.9 (m, DEPT +, BArF_24_), 134.8 (d, ^1^*J*_P-C_ = 47.5 Hz, no DEPT, -*Ph*), 133.5 (d, ^2^*J*_P-C_ = 19.3 Hz, DEPT +, -*Ph*), 131.8 (d, ^4^*J*_P-C_ = 2.6 Hz, DEPT +, -*Ph*), 129.0 (m, no DEPT, BArF_24_), 128.3 (d, ^3^*J*_P-C_ = 10.6 Hz, DEPT +, -*Ph*), 124.7 (q, ^1^*J*_F-C_ = 272.5 Hz, no DEPT, BArF_24_), 117.6 (m, DEPT +, BArF_24_), 74.6 (d, ^3^*J*_P-C_ = 7.1 Hz, DEPT +, -*C*_5_H_4_), 74.3 (d, ^1^*J*_P-C_ = 24.5 Hz, no DEPT, -*C*_5_H_4_), 74.2 (d, ^3^*J*_P-C_ = 6.8 Hz, DEPT +, -*C*_5_H_4_), 74.1 (d, ^1^*J*_P-C_ = 34.9 Hz, no DEPT, -*C*_5_H_4_), 73.5 (d, ^3^*J*_P-C_ = 5.5 Hz, DEPT +, -*C*_5_H_4_), 73.3 (d, ^2^*J*_P-C_ = 9.8 Hz, DEPT +, -*C*_5_H_4_), 73.1 (d, ^2^*J*_P-C_ = 6.2 Hz, DEPT +, -*C*_5_H_4_), 72.9 (d, ^1^*J*_P-C_ = 13.4 Hz, no DEPT, -*C*_5_H_4_), 71.7 (d, ^2^*J*_P-C_ = 8.4 Hz, DEPT +, -*C*_5_H_4_), 71.4 (s, DEPT +, *Cp*), 28.0 (d, ^1^*J*_P-C_ = 26.5 Hz, DEPT +, -*C*HMe_2_), 19.8 (s, DEPT +, -CH*Me*_2_). ^19^F{^1^H} NMR (CD_2_Cl_2_): δ −62.8 (s). ^31^P{^1^H} NMR (CD_2_Cl_2_): δ 64.1 (dd, ^2^*J*_P-P_ = 23.5 and 9.7 Hz, -*P*^i^Pr_2_), 58.8 (dd, ^2^*J*_P-P_ = 434.6 and 9.7, -*P*^i^Pr_2_), 20.0 (dd, ^2^*J*_P-P_ = 434.6 and 23.5 Hz, -*P*Ph_2_Fc). *Anal*. Calc. for C_76_H_67_BClF_24_Fe_2_P_3_Pd: C, 54.10; H, 4.00. Found: C, 54.25; H, 4.11%.

##### [Pd(dppdtbpf)(PMe_3_)Cl][BArF_24_]

The product was isolated as a red solid in 73% yield. ^1^H NMR (CD_2_Cl_2_) δ: 7.96 (m, 4H, -*Ph*), 7.64 (br s, 8H, BArF_24_), 7.55 (m, 6H, -*Ph*), 7.48 (br s, 4H, BArF_24_), 4.69 (AA′XX′, 2H, -C_5_*H*_4_), 4.44 (AA′XX′, 2H, -C_5_*H*_4_), 4.29 (AA′XX′, 2H, -C_5_*H*_4_), 4.16 (AA′XX′, 2H, -C_5_*H*_4_), 1.57 (s, 18H, -C*Me*_3_), 1.65 (d, ^2^*J*_P-H_ = 14.5 Hz, 9H, -P*Me*_3_). ^13^C{^1^H} NMR (CD_2_Cl_2_): δ 161.7 (q, ^1^*J*_B-C_ = 49.8 Hz, no DEPT, BArF_24_), 134.8 (m, DEPT +, BArF_24_), 134.1 (d, ^2^*J*_P-C_ = 13.6 Hz, DEPT +, -*Ph*), 138.4 (s, DEPT +, -*Ph*), 132.3 (d, ^1^*J*_P-C_ = 50.7 Hz, no DEPT, -*Ph*), 129.7 (d, ^3^*J*_P-C_ = 11.6 Hz, DEPT +, -*Ph*), 128.9 (m, no DEPT, BArF_24_), 124.6 (q, ^1^*J*_F-C_ = 272.4 Hz, no DEPT, BArF_24_), 117.5 (m, DEPT +, BArF_24_), 77.3 (d, ^2^*J*_P-C_ = 11.0 Hz, DEPT +, -*C_5_*H_4_), 76.5 (d, ^1^*J*_P-C_ = 61.5 Hz, no DEPT, -*C*_5_H_4_), 75.3 (d, ^3^*J*_P-C_ = 6.7 Hz, DEPT +, -*C_5_*H_4_), 73.2 (m, DEPT +, -*C_5_*H_4_), 41.0 (d, ^1^*J*_P-C_ = 14.3 Hz, no DEPT, -*C*Me_3_), 31.6 (s, DEPT +, -C*Me*_3_), 14.8 (d, ^1^*J*_P-C_ = 33.2 Hz, DEPT +, -P*C*H_3_). ^19^F{^1^H} NMR (CD_2_Cl_2_): δ −62.8 (s). ^31^P{^1^H} NMR (CD_2_Cl_2_): δ 74.0 (dd, ^2^*J*_P-P_ = 430.7 and 5.5 Hz, -*P*^t^Bu_2_), 43.5 (dd, ^2^*J*_P-P_ = 23.5 and 5.5 Hz, -*P*Ph_2_), −8.0 (dd, ^2^*J*_P-P_ = 430.7 and 23.5 Hz, -*P*Me_3_). *Anal*. Calc. for C_65_H_57_BClF_24_FeP_3_Pd: C, 52.42; H, 3.86. Found: C, 52.03; H, 3.75%.

##### [Pd(dppdtbpf)(PPh_3_)Cl][BArF_24_]

The product was isolated as a red solid in 98% yield. ^1^H NMR (CD_2_Cl_2_) δ: 8.00–7.05 (m, 37H, -*Ph* and BArF_24_), 7.48 (m, 8H, BArF_24_), 4.94 (AA′XX′, 2H, -C_5_*H*_4_), 4.59 (AA′XX′, 2H, -C_5_*H*_4_), 4.33 (AA′XX′, 2H, -C_5_*H*_4_), 3.63 (AA′XX′, 2H, -C_5_*H*_4_), 1.75 (d, ^3^*J*_P-H_ = 14.7 Hz, 18H, -*Me*). ^13^C{^1^H} NMR (CD_2_Cl_2_): δ 161.7 (q, ^1^*J*_B-C_ = 49.9 Hz, no DEPT, BArF_24_), 135.1 (d, ^3^*J*_P-C_ = 9.4 Hz, DEPT +, -*Ph*), 134.8 (m, DEPT +, BArF_24_), 133.6 (d, ^3^*J*_P-C_ = 9.0 Hz, DEPT +, -*Ph*), 132.5 (d, ^4^*J*_P-C_ = 2.8 Hz, DEPT +, -*Ph*), 130.8 (d, ^4^*J*_P-C_ = 2.9 Hz, DEPT +, -*Ph*), 129.9 (m, no DEPT, -*Ph*), 129.2 (d, ^2^*J*_P-C_ = 11.6 Hz, DEPT +, -*Ph*), 129.0 (m, no DEPT, -*Ph*), 128.6 (m, no DEPT, BArF_24_), 128.2 (d, ^2^*J*_P-C_ = 10.4 Hz, DEPT +, -*Ph*), 124.6 (q, ^1^*J*_F-C_ = 272.3 Hz, no DEPT, BArF_24_), 117.5 (m, DEPT +, BArF_24_), 77.3 (m, DEPT +, -*C_5_*H_4_), 75.7 (m, no DEPT, -*C_5_*H_4_), 73.3 (m, DEPT +, -*C_5_*H_4_), 42.5 (d, ^1^*J*_P-C_ = 15.4 Hz, no DEPT, -*C*Me_3_), 32.0 (s, DEPT +, -*Me*). ^19^F{^1^H} NMR (CD_2_Cl_2_): δ −62.8 (s). ^31^P{^1^H} NMR (CD_2_Cl_2_): δ 78.8 (dd, ^2^*J*_P-P_ = 423.0 and 6.2 Hz, -*P*^t^Bu_2_), 44.1 (dd, ^2^*J*_P-P_ = 19.6 and 6.2, -*P*Ph_2_), 24.2 (dd, ^2^*J*_P-P_ = 423.0 and 19.6 Hz, -*P*Ph_3_). *Anal*. Calc. for C_80_H_63_BClF_24_FeP_3_Pd: C, 57.35; H, 3.74. Found: C, 57.12; H, 3.84%.

##### [Pd(dppdtbpf)(PPh_2_Fc)Cl][BArF_24_]

The product was isolated as a red–purple solid in 66% yield. ^1^H NMR (CD_2_Cl_2_) δ: 7.90–6.90 (m, 24H, -*Ph* and BArF_24_), 5.25 (AA′XX′, 2H, -C_5_*H*_4_), 4.74 (AA′XX′, 2H, -C_5_*H*_4_), 4.42 (AA′XX′, 2H, -C_5_*H*_4_), 4.38 (AA′XX′, 2H, -C_5_*H*_4_), 4.17 (AA′XX′, 2H, -C_5_*H*_4_), 3.75 (s, 5H, *Cp*), 3.42 (AA′XX′, 2H, -C_5_*H*_4_), 1.63 (d, ^3^*J*_P-H_ = 14.5 Hz, 18H, -*Me*). ^13^C{^1^H} NMR (CD_2_Cl_2_): δ 161.9 (q, ^1^*J*_B-C_ = 49.7 Hz, no DEPT, BArF_24_), 134.9 (m, DEPT +, BArF_24_), 132.5 (s, DEPT +, -*Ph*), 130.3 (s, no DEPT, -*Ph*), 129.4 (d, ^2^*J*_P-C_ = 11.4 Hz, DEPT +, -*Ph*), 129.0 (m, no DEPT, BArF_24_), 127.3 (d, ^3^*J*_P-C_ = 10.4 Hz, DEPT +, -*Ph*), 124.7 (q, ^1^*J*_F-C_ = 272.2 Hz, no DEPT, BArF_24_), 117.6 (m, DEPT +, BArF_24_), 77.4 (m, DEPT +, -*C_5_*H_4_), 77.1 (m, DEPT +, -*C_5_*H_4_), 75.7 (m, no DEPT, -*C_5_*H_4_), 73.3 (m, DEPT +, -*C*_5_H_4_), 71.4 (s, DEPT +, *Cp*), 70.8 (m, DEPT +, -*C*_5_H_4_), 42.4 (d, ^1^*J*_P-C_ = 14.9 Hz, no DEPT, -*C*Me_3_), 32.1 (s, DEPT +, -*Me*). ^19^F{^1^H} NMR (CD_2_Cl_2_): δ −62.8 (s). ^31^P{^1^H} NMR (CD_2_Cl_2_): δ 74.4 (dd, ^2^*J*_P-P_ = 429.6 and 6.1 Hz, -*P*^t^Bu_2_), 43.0 (dd, ^2^*J*_P-P_ = 18.1 and 6.1, -*P*Ph_2_), 22.8 (dd, ^2^*J*_P-P_ = 429.6 and 18.1 Hz, -*P*Ph_2_Fc). *Anal*. Calc. for C_82_H_67_BClF_24_Fe_2_P_3_Pd: C, 55.98; H, 3.84. Found: C, 56.11; H, 3.93%.

##### [Pd(dcpf)(PMe_3_)Cl][BArF_24_]

The product was isolated as an orange solid in 74% yield. ^1^H NMR (CD_2_Cl_2_) δ: 7.64 (br s, 8H, BArF_24_), 7.48 (br s, 4H, BArF_24_), 4.51 (AA′XX′, 2H, -C_5_*H*_4_), 4.49 (AA′XX′, 2H, -C_5_*H*_4_), 4.45 (m, 4H, -C_5_*H*_4_), 2.71 (m, 2H, -*Cy*), 2.03–1.04 (m, 40H, -*Cy*), 1.68 (d, ^2^*J*_P-H_ = 11.1 Hz, 9H, -*Me*), 1.08 (m, 2H, -*Cy*). ^13^C{^1^H} NMR (CD_2_Cl_2_): δ 161.9 (q, ^1^*J*_B-C_ = 49.7 Hz, no DEPT, BArF_24_), 134.9 (m, DEPT +, BArF_24_), 129.0 (m, no DEPT, BArF_24_), 124.7 (q, ^1^*J*_F-C_ = 272.3 Hz, no DEPT, BArF_24_), 117.6 (m, DEPT +, BArF_24_), 77.0 (d, ^1^*J*_P-C_ = 10.9 Hz, no DEPT, -*C_5_*H_4_), 76.5 (d, ^1^*J*_P-C_ = 10.0 Hz, no DEPT, -*C_5_*H_4_), 74.9 (d, ^2^*J*_P-C_ = 7.9 Hz, DEPT +, -*C_5_*H_4_), 74.3 (d, ^3^*J*_P-C_ = 6.6 Hz, DEPT +, -*C_5_*H_4_), 73.4 (d, ^3^*J*_P-C_ = 5.6 Hz, DEPT +, -*C_5_*H_4_), 73.2 (d, ^3^*J*_P-C_ = 6.5 Hz, DEPT +, -*C_5_*H_4_), 39.0 (d, ^1^*J*_P-C_ = 38.8 Hz, DEPT + -*Cy*), 37.8 (d, ^1^*J*_P-C_ = 24.4 Hz, DEPT +, -*Cy*), 31.1 (d, ^3^*J*_P-C_ = 11.0 Hz, DEPT -, -*Cy*), 30.7 (d, ^3^*J*_P-C_ = 13.9 Hz, DEPT -, -*Cy*), 27.5 (dd, *J*_P-C_ = 28.5 and 13.4 Hz, DEPT -, -*Cy*), 26.9 (dd, *J*_P-C_ = 21.4 and 10.7 Hz, DEPT -, -*Cy*), 26.0 (s, DEPT -, -*Cy*), 25.6 (s, DEPT -, -*Cy*), 17.4 (dd, *J*_P-C_ = 29.9 and 2.6 Hz, DEPT +, -*Me*). ^19^F{^1^H} NMR (CD_2_Cl_2_): δ −62.8 (s). ^31^P{^1^H} NMR (CD_2_Cl_2_): δ 60.6 (dd, ^2^*J*_P-P_ = 30.4 and 13.6, -*P*Cy_2_), 50.2 (dd, ^2^*J*_P-P_ = 443.0 and 13.6 Hz, -*P*Cy_2_), −11.0 (dd, ^2^*J*_P-P_ = 443.0 and 30.4 Hz, -*P*Me_3_). *Anal*. Calc. for C_69_H_73_BClF_24_FeP_3_Pd: C, 53.35; H, 4.74. Found: C, 53.04; H, 4.51%.

##### [Pd(dfurpf)(PMe_3_)Cl][BArF_24_]

The product was isolated as a red–orange solid in 86% yield. ^1^H NMR (CDCl_3_) δ: 7.64 (br s, 8H, BArF_24_), 7.44 (br s, 4H, BArF_24_), 6.99 (br s, 2H, -*furanyl*), 6.27 (br s, 2H, -*furanyl*), 6.12 (br s, 2H, -*furanyl*), 5.79 (br s, 2H, -*furanyl*), 4.44 (AA′XX′, 2H, -C_5_*H*_4_), 4.41 (AA′XX′, 2H, -C_5_*H*_4_), 4.34 (AA′XX′, 2H, -C_5_*H*_4_), 3.93 (AA′XX′, 2H, -C_5_*H*_4_), 2.14 (d, ^2^*J*_P-H_ = 15.0 Hz, 9H, -*Me*), 1.19 (s, 12H, -*Me*). ^13^C{^1^H} NMR (CDCl_3_): δ 160.7 (q, ^1^*J*_B-C_ = 49.5 Hz, no DEPT, BArF_24_), 158.3 (d, ^1^*J*_P-C_ = 8.1 Hz, no DEPT, *-furanyl*), 139.0 (m, no DEPT, -*furanyl*), 133.6 (m, DEPT +, BArF_24_), 130.5 (d, ^3^*J*_P-C_ = 2.9 Hz, DEPT +, -*furanyl*), 127.9 (m, no DEPT, BArF_24_), 125.2 (d, ^2^*J*_P-C_ = 17.0 Hz, DEPT +, -*furanyl*), 124.8 (d, ^2^*J*_P-C_ = 17.0 Hz, DEPT +, -*furanyl*), 123.5 (q, ^1^*J*_F-C_ = 272.4 Hz, no DEPT, BArF_24_), 116.4 (m, DEPT +, BArF_24_), 108.0 (d, ^3^*J*_P-C_ = 7.3 Hz, -*furanyl*), 107.4 (d, ^3^*J*_P-C_ = 7.3 Hz, -*furanyl*), 75.1 (d, ^2^*J*_P-C_ = 13.9 Hz, DEPT +, -*C_5_*H_4_), 74.1 (d, ^2^*J*_P-C_ = 11.6 Hz, DEPT +, -*C_5_*H_4_), 73.7 (d, ^3^*J*_P-C_ = 9.3 Hz, DEPT +, -*C_5_*H_4_), 73.4 (d, ^3^*J*_P-C_ = 7.2 Hz, DEPT +, -*C_5_*H_4_), 28.7 (s, DEPT +, furanyl-*C*H_3_), 12.8 (d, ^1^*J*_P-C_ = 60.1 Hz, DEPT +, -P*C*H_3_), 32.1 (s, DEPT +, -*Me*). ^19^F{^1^H} NMR (CDCl_3_): δ −62.4 (s). ^31^P{^1^H} NMR (CDCl_3_): δ 31.4 (dd, ^2^*J*_P-P_ = 493.0 and 15.7, -*P*Ph_2_), −5.9 (dd, ^2^*J*_P-P_ = 15.7 and 12.9 Hz, -*P*(furanyl)_2_), −9.4 (dd, ^2^*J*_P-P_ = 493.0 and 12.9 Hz, -*P*(furanyl)_2_. *Anal*. Calc. for C_65_H_49_BClF_24_FeO_4_P_3_Pd: C, 50.53; H, 3.20. Found: C, 50.34; H, 3.11%.

##### [Pd(dfurpf)(PPh_3_)Cl][BArF_24_]

The product was isolated as a red–orange solid in 57% yield. ^1^H NMR (CDCl_2_) δ: 7.65 (br s, 8H, BArF_24_), 7.50–7.20 (m, 19H, -*Ph* and BArF_24_), 6.99 (br s, 2H, -*furanyl*), 6.33 (br s, 2H, -*furanyl*), 6.16 (br s, 2H, -*furanyl*), 5.86 (br s, 2H, -*furanyl*), 4.44 (AA′XX′, 2H, -C_5_*H*_4_), 4.39 (AA′XX′, 4H, -C_5_*H*_4_), 4.01 (AA′XX′, 2H, -C_5_*H*_4_), 2.34 (s, 6H, -*Me*), 2.01 (s, 6H, -*Me*). ^13^C{^1^H} NMR (CD_2_Cl_2_): δ 161.9 (q, ^1^*J*_B-C_ = 49.8 Hz, no DEPT, BArF_24_), 159.8 (d, ^1^*J*_P-C_ = 5.9 Hz, no DEPT, *-furanyl*), 159.7 (d, ^1^*J*_P-C_ = 6.6 Hz, no DEPT, -*furanyl*), 139.9 (m, DEPT +, BArF_24_), 135.4 (d, ^2^*J*_P-C_ = 10.3 Hz, DEPT +, -*Ph*), 134.9 (s, DEPT +, -*furanyl*), 134.8 (s, DEPT +, -*furanyl*), 134.7 (s, DEPT +, -*furanyl*), 129.0 (m, no DEPT, BArF_24_), 128.6 (d, ^3^*J*_P-C_ = 6.6 Hz, DEPT +, -*Ph*), 128.5 (s, DEPT +, -*furanyl*), 128.4 (s, DEPT +, -*furanyl*), 126.3 (d, ^1^*J*_P-C_ = 16.7 Hz, no DEPT, -*Ph*), 126.0 (s, DEPT +, -*Ph*), 125.9 (m, DEPT +, -*furanyl*), 124.7 (q, ^1^*J*_F-C_ = 272.2 Hz, no DEPT, BArF_24_), 117.6 (m, DEPT +, BArF_24_), 109.2 (s, DEPT +, -*furanyl*), 109.1 (s, DEPT +, -*furanyl*), 108.4 (s, DEPT +, -*furanyl*), 108.3 (s, DEPT +, -*furanyl*), 76.1 (d, ^2^*J*_P-C_ = 13.9 Hz, DEPT +, -*C_5_*H_4_), 75.4 (d, ^2^*J*_P-C_ = 11.3 Hz, DEPT +, -*C_5_*H_4_), 74.9 (d, ^3^*J*_P-C_ = 9.4 Hz, DEPT +, -*C_5_*H_4_), 74.7 (d, ^3^*J*_P-C_ = 7.4 Hz, DEPT +, -*C_5_*H_4_), 74.3 (m, no DEPT, -*C*_5_H_4_), 73.4 (m, no DEPT, -*C_5_*H_4_), 14.0 (s, DEPT +, furanyl-*C*H_3_), 13.5 (s, DEPT +, -furanyl-*C*H_3_). ^19^F{^1^H} NMR (CD_2_Cl_2_): δ −62.8 (s). ^19^F{^1^H} NMR (CD_2_Cl_2_): δ −62.8 (s). ^31^P{^1^H} NMR (CD_2_Cl_2_): δ 31.8 (dd, ^2^*J*_P-P_ = 491.0 and 17.1, -*P*Ph_2_Fc), −5.8 (dd, ^2^*J*_P-P_ = 17.1 and 11.7 Hz, -*P*(furanyl)_2_), −9.0 (dd, ^2^*J*_P-P_ = 491.0 and 11.7 Hz, -*P*(furanyl)_2_). *Anal*. Calc. for C_80_H_52_BClF_24_FeO_4_P_3_Pd: C, 55.94; H, 3.03. Found: C, 56.23; H, 2.94%.

##### [Pd(dfurpf)(PPh_2_Fc)Cl][BArF_24_]

The product was isolated as a dark red solid in 95% yield. ^1^H NMR (CDCl_2_) δ: 7.64 (br s, 8H, BArF_24_), 7.58–7.45 (m, 8H, -*Ph* and BArF_24_), 7.39 (t, 2H, *J*_H-H_ = 7.5 Hz, -*Ph*), 7.28 (m, 4H, -*Ph*), 6.97 (br s, 2H, -*furanyl*), 6.45 (br s, 2H, -*furanyl*), 6.16 (br s, 2H, -*furanyl*), 5.89 (br s, 2H, -*furanyl*), 4.41 (AA′XX′, 2H, -C_5_*H*_4_), 4.36 (AA′XX′, 2H, -C_5_*H*_4_), 4.09 (AA′XX′, 2H, -C_5_*H*_4_), 4.01 (AA′XX′, 2H, -C_5_*H*_4_), 3.85 (s, 5H, -*Cp*), 2.36 (s, 6H, -*Me*), 2.03 (s, 6H, -*Me*). ^13^C{^1^H} NMR (CD_2_Cl_2_): δ 161.9 (q, ^1^*J*_B-C_ = 50.0 Hz, no DEPT, BArF_24_), 159.7 (d, ^1^*J*_P-C_ = 5.9 Hz, no DEPT, *-furanyl*), 159.4 (d, ^1^*J*_P-C_ = 6.5 Hz, no DEPT, -*furanyl*), 134.9 (m, DEPT +, BArF_24_), 134.2 (s, DEPT +, -*furanyl*), 134.0 (s, DEPT +, -*furanyl*), 131.4 (s, DEPT +, -*furanyl*), 129.0 (m, no DEPT, BArF_24_), 128.2 (s, DEPT +, -*furanyl*), 128.1 (s, DEPT +, -*furanyl*), 125.9 (m, DEPT +, -*furanyl*), 124.7 (q, ^1^*J*_F-C_ = 272.6 Hz, no DEPT, FArF_24_), 117.6 (m, DEPT +, BArF_24_), 109.1 (s, DEPT +, -*furanyl*), 109.0 (s, DEPT +, -*furanyl*), 108.4 (s, DEPT +, -*furanyl*), 108.3 (s, DEPT +, -*furanyl*), 75.9 (d, ^2^*J*_P-C_ = 9.2 Hz, DEPT +, -*C_5_*H_4_), 75.3 (m, DEPT +, -*C_5_*H_4_), 74.7 (d, ^3^*J*_P-C_ = 9.2 Hz, DEPT +, -*C_5_*H_4_), 74.4 (d, ^3^*J*_P-C_ = 9.2 Hz, DEPT +, -*C_5_*H_4_), 71.4 (m, no DEPT, -*C*_5_H_4_), 70.7 (s, DEPT +, *Cp*), 14.0 (s, DEPT +, furanyl-*C*H_3_), 13.6 (s, DEPT +, -furanyl-*C*H_3_). ^19^F{^1^H} NMR (CD_2_Cl_2_): δ −62.8 (s). ^31^P{^1^H} NMR (CD_2_Cl_2_): δ 30.1 (dd, ^2^*J*_P-P_ = 500.8 and 15.6, -*P*Ph_2_Fc), −5.9 (dd, ^2^*J*_P-P_ = 17.6 and 15.6 Hz, -*P*(furanyl)_2_), −10.6 (dd, ^2^*J*_P-P_ = 500.8 and 17.3 Hz, -*P*(furanyl)_2_). *Anal*. Calc. for C_84_H_59_BClF_24_Fe_2_O_4_P_3_Pd: C, 57.77; H, 3.41. Found: C, 57.44; H, 3.42%.

##### [Pd(dppf)(P(NMe_2_)_3_)Cl][BArF_24_]

The product was isolated as a red solid in 86% yield. ^1^H NMR (CD_2_Cl_2_) δ: 8.06–7.06 (m, 32H, -*Ph* and BArF_24_), 4.65 (AA′XX′, 2H, -C_5_*H*_4_), 4.59 (AA′XX′, 2H, -C_5_*H*_4_), 4.13 (AA′XX′, 2H, -C_5_*H*_4_), 3.18 (AA′XX′, 2H, -C_5_*H*_4_), 2.56 (s, 18H, -*Me*). ^13^C{^1^H} NMR (CD_2_Cl_2_): δ 161.9 (q, ^1^*J*_B-C_ = 51.1 Hz, no DEPT, BArF_24_), 134.9 (m, DEPT +, BArF_24_), 132.4 (s, DEPT +, -*Ph*), 131.4 (s, DEPT +, -*Ph*), 128.9 (m, DEPT +, -*Ph* and BArF_24_), 124.7 (q, ^1^*J*_F-C_ = 272.2 Hz, no DEPT, BArF_24_), 117.6 (m, DEPT +, BArF_24_), 77.6 (d, ^1^*J*_P-C_ = 12.6 Hz, DEPT +, -*C*_5_H_4_), 76.4 (m, no DEPT, -*C*_5_H_4_), 75.7 (d, ^2^*J*_P-C_ = 6.8 Hz, DEPT +, -*C*_5_H_4_), 75.3 (d, ^2^*J*_P-C_ = 5.9 Hz, DEPT +, -*C*_5_H_4_), 73.9 (d, ^3^*J*_P-C_ = 5.5 Hz, no DEPT, -*C*_5_H_4_), 40.1 (d, ^2^*J*_P-C_ = 7.8 Hz, DEPT +, -*Me*). ^19^F{^1^H} NMR (CD_2_Cl_2_): δ −62.8 (s). ^31^P{^1^H} NMR (CD_2_Cl_2_): δ 94.3 (dd, ^2^*J*_P-P_ = 607.0 and 10.0 Hz, -*P*(NMe_2_)_3_), 32.2 (dd, ^2^*J*_P-P_ = 17.5 and 9.9, -*P*Ph_2_), 19.0 (dd, ^2^*J*_P-P_ = 607.0 and 17.5 Hz, -*P*Ph_2_). *Anal*. Calc. for C_52_H_58_BClF_24_FeN_3_P_3_Pd: C, 45.39; H, 4.25. Found: C, 45.01; H, 3.99%.

##### [Pd(dppf)(P*^i^*Pr_3_)Cl][BArF_24_]

The product was isolated as a red solid in 75% yield. ^1^H NMR (CD_2_Cl_2_) δ: 7.73 (dd, *J*_H-H_ = 11.4 and 7.7 Hz, 4H, -*Ph*), 7.64 (m, 8H, BArF_24_), 7.48 (m, 4H, BArF_24_), 7.45 (m, 12H, -*Ph*), 7.16 (t, *J*_H-H_ = 8.2 Hz, -*Ph*), 4.89 (AA′XX′, 2H, -C_5_*H*_4_), 4.68 (AA′XX′, 2H, -C_5_*H*_4_), 4.23 (AA′XX′, 2H, -C_5_*H*_4_), 3.00 (AA′XX′, 2H, -C_5_*H*_4_), 2.11 (m, 3H, -C*H*Me_2_). 1.17 (d, *J*_H-H_ = 14.7 Hz, -*Me*), 1.15 (d, *J*_H-H_ = 14.7 Hz, -*Me*). ^13^C{^1^H} NMR (CD_2_Cl_2_): δ 161.7 (q, ^1^*J*_B-C_ = 49.9 Hz, no DEPT, BArF_24_), 134.7 (m, DEPT +, BArF_24_), 134.6 (s, DEPT +, -*Ph*), 134.5 (s, DEPT +, -*Ph*), 133.4 (d, ^1^*J*_P-C_ = 53.5 Hz, no DEPT, -*Ph*), 132.9 (d, ^3^*J*_P-C_ = 2.6 Hz, DEPT +, -*Ph*), 131.4 (d, ^3^*J*_P-C_ = 2.6 Hz, DEPT +, -*Ph*), 129.0 (m, no DEPT, BArF_24_), 128.8 (d, ^2^*J*_P-C_ = 11.3 Hz, DEPT +, -*Ph*), 128.6 (d ^2^*J*_P-C_ = 10.6 Hz, DEPT +, -*Ph*), 128.6 (d ^2^*J*_P-C_ = 11.6 Hz, DEPT +, -*Ph*), 124.6 (q, ^1^*J*_F-C_ = 272.6 Hz, no DEPT, BArF_24_), 117.4 (m, DEPT +, BArF_24_), 79.0 (d, ^2^*J*_P-C_ = 13.8 Hz, DEPT +, -*C*_5_H_4_), 76.0 (d, ^3^*J*_P-C_ = 8.0 Hz, DEPT +, -*C*_5_H_4_), 75.5 (d, ^2^*J*_P-C_ = 8.8 Hz, DEPT +, -*C*_5_H_4_), 74.2 (d, ^3^*J*_P-C_ = 5.8 Hz, DEPT +, -*C*_5_H_4_), 68.4 (d, ^1^*J*_P-C_ = 46.4Hz, no DEPT, -*C*_5_H_4_), 24.8 (d, ^1^*J*_P-C_ = 20.5 Hz, DEPT +, -*C*HMe_2_), 20.3 (s, DEPT +, -*Me*). ^19^F{^1^H} NMR (CD_2_Cl_2_): δ −62.8 (s). ^31^P{^1^H} NMR (CD_2_Cl_2_): δ 50.6 (dd, ^2^*J*_P-P_ = 450.0 and 17.6 Hz, -*P*^i^Pr_3_), 32.7 (dd, ^2^*J*_P-P_ = 17.6 and 5.9, -*P*Ph_2_), 21.6 (dd, ^2^*J*_P-P_ = 450.0 and 5.9 Hz, -*P*Ph_2_). *Anal*. Calc. for C_75_H_61_BClF_24_FeP_3_Pd: C, 55.83; H, 3.81. Found: C, 56.02; H, 3.97%.

##### [Pd(dppf)(P(CH_2_Ph)_3_)Cl][BArF_24_]

The product was isolated as an orange–red solid in 84% yield. ^1^H NMR (CD_2_Cl_2_) δ: 7.86–6.94 (m, 47H, -*Ph* and BArF_24_), 4.41 (AA′XX′, 2H, -C_5_*H*_4_), 4.37 (AA′XX′, 2H, -C_5_*H*_4_), 3.99 (AA′XX′, 2H, -C_5_*H*_4_), 3.80 (AA′XX′, 2H, -C_5_*H*_4_), 2.93 (d, ^2^*J*_P-H_ = 9.4 Hz, 6H, -C*H*_2_Ph). ^13^C{^1^H} NMR (CD_2_Cl_2_): δ 161.9 (q, ^1^*J*_B-C_ = 50.0 Hz, no DEPT, BArF_24_), 134.9 (m, DEPT +, BArF_24_), 134.6 (d, ^2^*J*_P-C_ = 10.9 Hz, DEPT +, -*Ph*), 134.4 (d, ^2^*J*_P-C_ = 12.5 Hz, DEPT +, -*Ph*), 133.2 (m, no DEPT, -*Ph*), 133.2 (s, DEPT +, -*Ph*), 132.0 (d, ^3^*J*_P-C_ = 2.8 Hz, -*Ph*), 131.2 (m, no DEPT, -*Ph*), 130.4 (d, ^3^*J*_P-C_ = 5.3 Hz, DEPT +, -*Ph*), 129.6 (d, ^2^*J*_P-C_ = 11.6 Hz, -*Ph*), 129.4 (d, ^3^*J*_P-C_ = 2.2 Hz, DEPT +, -*Ph*), 129.0 (m, no DEPT, BArF_24_), 128.8 (d, ^2^*J*_P-C_ = 10.8 Hz, DEPT +, *-Ph*), 128.5 (m, no DEPT, -*Ph*), 127.9 (d, ^3^*J*_P-C_ = 2.9 Hz, -*Ph*), 124.7 (q, ^1^*J*_F-C_ = 272.3 Hz, no DEPT, BArF_24_), 117.6 (m, DEPT +, BArF_24_), 77.2 (d, ^2^*J*_P-C_ = 12.3 Hz, DEPT +, -*C*_5_H_4_), 76.8 (d, ^2^*J*_P-C_ = 10.0 Hz, DEPT +, -*C*_5_H_4_), 75.1 (d, ^3^*J*_P-C_ = 2.2 Hz, DEPT +, -*C*_5_H_4_), 75.0 (d, ^3^*J*_P-C_ = 4.1 Hz, DEPT +, -*C*_5_H_4_), 69.7 (m, no DEPT, -*C_5_*H_4_), 69.2 (m, no DEPT, -*C_5_*H_4_), 29.4 (dd, *J* = 19.9 and 2.9 Hz, DEPT -, -*C*H_2_Ph). ^19^F{^1^H} NMR (CD_2_Cl_2_): δ −62.8 (s). ^31^P{^1^H} NMR (CD_2_Cl_2_): δ 34.0 (dd, ^2^*J*_P-P_ = 16.0 and 6.5 Hz, -*P*Ph_2_), 30.1 (dd, ^2^*J*_P-P_ = 452.4 and 6.5, -*P*Ph_2_), 18.9 (dd, ^2^*J*_P-P_ = 452.4 and 16.0 Hz, -*P*Ph_3_). *Anal*. Calc. for C_87_H_61_BClF_24_FeP_3_Pd: C, 59.46; H, 3.50. Found: C, 59.14; H, 3.65%.

##### [Pd(dppf)(P(*m*-tolyl)_3_)Cl][BArF_24_]

The product was isolated as an orange–red solid in 72% yield. ^1^H NMR (CD_2_Cl_2_) δ: 8.03–7.00 (m, 44H, -*Ph*, -*tol*, and BArF_24_), 4.74 (AA′XX′, 2H, -C_5_*H*_4_), 4.67 (AA′XX′, 2H, -C_5_*H*_4_), 4.36 (AA′XX′, 2H, -C_5_*H*_4_), 3.55 (AA′XX′, 2H, -C_5_*H*_4_), 2.23 (s, 9H, -C*H*_3_). ^13^C{^1^H} NMR (CD_2_Cl_2_): δ 161.9 (q, ^1^*J*_B-C_ = 49.9 Hz, no DEPT, BArF_24_), 138.6 (s, DEPT +, -*Ph*), 138.5 (s, DEPT +, -*Ph*), 134.7 (m, DEPT +, BArF_24_), 134.2 (m, no DEPT, -*Ph*), 132.1 (d, ^3^*J*_P-C_ = 2.8 Hz, DEPT +, -*Ph*), 131.7 (d, ^3^*J*_P-C_ = 2.6 Hz, DEPT +, -*Ph*), 131.4 (s, DEPT +, -*Ph*), 130.5 (m, no DEPT, -*Ph*), 129.2 (d, ^2^*J*_P-C_ = 10.1 Hz, DEPT +, -*Ph*), 129.0 (m, no DEPT, BArF_24_), 128.7 (d ^2^*J*_P-C_ = 9.8 Hz, DEPT +, -*Ph*), 128.6 (d ^2^*J*_P-C_ = 11.6 Hz, DEPT +, -*Ph*), 128.2 (m, no DEPT, -*Ph*), 125.6 (m, DEPT +, -*Ph*), 124.7 (q, ^1^*J*_F-C_ = 272.3 Hz, no DEPT, BArF_24_), 117.6 (m, DEPT +, BArF_24_), 77.6 (d, ^2^*J*_P-C_ = 12.3 Hz, DEPT +, -*C*_5_H_4_), 76.0 (d, ^2^*J*_P-C_ = 12.3 Hz, no DEPT, -*C*_5_H_4_), 76.0 (d, ^3^*J*_P-C_ = 8.1 Hz, DEPT +, -*C*_5_H_4_), 75.4 (d, ^2^*J*_P-C_ = 8.1 Hz, DEPT +, -*C*_5_H_4_), 74.6 (d, ^3^*J*_P-C_ = 6.1 Hz, DEPT +, -*C*_5_H_4_), 21.2 (s, DEPT +, -*C*H_3_). ^19^F{^1^H} NMR (CD_2_Cl_2_): δ −62.9 (s). ^31^P{^1^H} NMR (CD_2_Cl_2_): δ 35.2 (dd, ^2^*J*_P-P_ = 17.5 and 5.8 Hz, -*P*Ph_2_), 30.2 (dd, ^2^*J*_P-P_ = 478.7 and 17.5, -*P*(*m*-tol)_3_), 26.5 (dd, ^2^*J*_P-P_ = 478.7 and 5.8 Hz, -*P*Ph_2_). *Anal*. Calc. for C_87_H_64_BClF_24_FeP_3_Pd: C, 59.35; H, 3.66. Found: C, 59.28; H, 3.72%.

##### [Pd(dppf)(P(*p*-tolyl)_3_)Cl][BArF_24_]

The product was isolated as an orange–red solid in 88% yield. ^1^H NMR (CD_2_Cl_2_) δ: 7.89–6.91 (m, 44H, -*Ph*, -*tol*, and BArF_24_), 4.56 (AA′XX′, 2H, -C_5_*H*_4_), 4.51 (AA′XX′, 2H, -C_5_*H*_4_), 4.23 (AA′XX′, 2H, -C_5_*H*_4_), 3.50 (AA′XX′, 2H, -C_5_*H*_4_), 2.24 (s, 9H, -C*H*_3_). ^13^C{^1^H} NMR (CD_2_Cl_2_): δ 161.7 (q, ^1^*J*_B-C_ = 49.6 Hz, no DEPT, BArF_24_), 142.3 (s, DEPT +, -*Ph*), 134.7 (m, DEPT +, BArF_24_), 134.2 (m, no DEPT, -*Ph*), 132.1 (d, ^3^*J*_P-C_ = 2.8 Hz, DEPT +, -*Ph*), 131.7 (d, ^3^*J*_P-C_ = 2.6 Hz, DEPT +, -*Ph*), 131.4 (s, DEPT +, -*Ph*), 130.5 (m, no DEPT, -*Ph*), 129.2 (d, ^2^*J*_P-C_ = 10.1 Hz, DEPT +, -*Ph*), 129.0 (m, no DEPT, BArF_24_), 128.7 (d ^2^*J*_P-C_ = 9.8 Hz, DEPT +, -*Ph*), 128.6 (d ^2^*J*_P-C_ = 11.6 Hz, DEPT +, -*Ph*), 128.2 (m, no DEPT, -*Ph*), 125.6 (m, DEPT +, -*Ph*), 124.6 (q, ^1^*J*_F-C_ = 272.3 Hz, no DEPT, BArF_24_), 117.5 (m, DEPT +, BArF_24_), 77.3 (d, ^2^*J*_P-C_ = 12.0 Hz, DEPT +, -*C*_5_H_4_), 76.5 (m, no DEPT, -*C*_5_H_4_), 75.8 (d, ^3^*J*_P-C_ = 8.2 Hz, DEPT +, -*C*_5_H_4_), 75.2 (d, ^2^*J*_P-C_ = 9.7 Hz, DEPT +, -*C*_5_H_4_), 74.4 (d, ^3^*J*_P-C_ = 5.6 Hz, DEPT +, -*C*_5_H_4_), 75.2 (m, no DEPT, -*C*_5_H_4_), 21.1 (s, DEPT +, -*C*H_3_). ^19^F{^1^H} NMR (CD_2_Cl_2_): δ −62.8 (s). ^31^P{^1^H} NMR (CD_2_Cl_2_): δ 35.2 (dd, ^2^*J*_P-P_ = 17.6 and 5.9 Hz, -*P*Ph_2_), 30.3 (dd, ^2^*J*_P-P_ = 479.3 and 17.6, -*P*(*p*-tol)_3_), 26.5 (dd, ^2^*J*_P-P_ = 479.3 and 5.9 Hz, -*P*Ph_2_). *Anal*. Calc. for C_87_H_64_BClF_24_FeP_3_Pd: C, 59.35; H, 3.66. Found: C, 59.28; H, 3.72%.

##### [Pd(dppf)(P(*p*-C_6_H_4_OMe)_3_)Cl][BArF_24_]

The product was isolated as an orange–red solid in 45% yield. ^1^H NMR (CD_2_Cl_2_) δ: 7.90–6.98 (m, 44H, -*Ph*, -C_6_*H*_4_OMe, and BArF_24_), 4.53 (AA′XX′, 2H, -C_5_*H*_4_), 4.50 (AA′XX′, 2H, -C_5_*H*_4_), 4.23 (AA′XX′, 2H, -C_5_*H*_4_), 3.70 (s, 9H, -OC*H*_3_), 3.54 (AA′XX′, 2H, -C_5_*H*_4_). ^13^C{^1^H} NMR (CD_2_Cl_2_): δ 162.2 (d, ^4^*J*_P-C_ = 1.5 Hz, no DEPT, -O*C*H_3_), 161.9 (q, ^1^*J*_B-C_ = 49.8 Hz, no DEPT, BArF_24_), 136.1 (d, ^3^*J*_P-C_ = 4.4 Hz, DEPT +, -*Ph*), 136.0 (d, ^3^*J*_P-C_ = 4.4 Hz, DEPT +, -*Ph*), 134.9 (m, DEPT +, BArF_24_), 134.2 (d, ^2^*J*_P-C_ = 12.1 Hz, DEPT +, -*Ph*), 132.3 (d, ^3^*J*_P-C_ = 2.9 Hz, DEPT +, -*Ph*), 131.8 (s, DEPT +, -*Ph*), 130.8 (m, no DEPT, -*Ph*), 129.2 (m, no DEPT, BArF_24_), 128.9 (d, ^2^*J*_P-C_ = 3.3 Hz, DEPT +, -*Ph*), 128.8 (d, ^2^*J*_P-C_ = 11.6 Hz, DEPT +, -*Ph*), 124.7 (q, ^1^*J*_F-C_ = 272.3 Hz, no DEPT, BArF_24_), 120.1 (m, no DEPT, -*Ph*), 117.6 (m, DEPT +, BArF_24_), 114.2 (d, ^3^*J*_P-C_ = 3.2 Hz, DEPT +, -*Ph*), 114.1 (d, ^3^*J*_P-C_ = 3.4 Hz, DEPT +, -*Ph*), 77.3 (d, ^2^*J*_P-C_ = 12.1 Hz, DEPT +, -*C*_5_H_4_), 76.0 (d, ^3^*J*_P-C_ = 6.8 Hz, DEPT +, -*C*_5_H_4_), 75.3 (d, ^2^*J*_P-C_ = 8.2 Hz, DEPT +, -*C*_5_H_4_), 74.5 (d, ^3^*J*_P-C_ = 5.0 Hz, DEPT +, -*C*_5_H_4_), 69.8 (m, no DEPT, -*C*_5_H_4_), 69.1 (m, no DEPT, -*C*_5_H_4_), 68.2 (s, DEPT +, -O*C*H_3_). ^19^F{^1^H} NMR (CD_2_Cl_2_): δ −62.8 (s). ^31^P{^1^H} NMR (CD_2_Cl_2_): δ 50.5 (dd, ^2^*J*_P-P_ = 451.9 and 17.7 Hz, -*P*(*p*-C_6_H_4_OMe)_3_), 32.6 (dd, ^2^*J*_P-P_ = 17.7 and 6.3, -*P*Ph_2_), 21.6 (dd, ^2^*J*_P-P_ = 451.9 and 6.3 Hz, -*P*Ph_2_). *Anal*. Calc. for C_87_H_64_BClF_24_FeO_3_P_3_Pd: C, 57.78; H, 3.57. Found: C, 58.09; H, 3.71%.

##### [Pd(dppf)(P(*p*-C_6_H_4_F)_3_)Cl][BArF_24_]

The product was isolated as an orange–red solid in 75% yield. ^1^H NMR (CD_2_Cl_2_) δ: 7.90–6.78 (m, 44H, -*Ph*, -C_6_*H*_4_F, and BArF_24_), 4.48 (m, 4H, -C_5_*H*_4_), 4.28 (AA′XX′, 2H, -C_5_*H*_4_), 3.69 (AA′XX′, 2H, -C_5_*H*_4_). ^13^C{^1^H} NMR (CD_2_Cl_2_): δ 163.6 (d, ^1^*J*_F-C_ = 248.6 Hz, no DEPT, *C*F), 161.9 (q, ^1^*J*_B-C_ = 49.6 Hz, no DEPT, BArF_24_), 134.9 (m, DEPT +, BArF_24_), 133.9 (d, ^2^*J*_P-C_ = 12.2 Hz, DEPT +, -*Ph*), 132.9 (m, DEPT +, -*Ph*), 132.2 (s, DEPT +, -*Ph*), 131.8 (s, DEPT +, -*Ph*), 130.1 (m, no DEPT, -*Ph*), 129.2 (m, no DEPT, BArF_24_), 128.9 (d, ^2^*J*_P-C_ = 3.3 Hz, DEPT +, -*Ph*), 128.8 (m, no DEPT +, -*Ph*), 124.7 (q, ^1^*J*_F-C_ = 272.2 Hz, no DEPT, BArF_24_), 124.3 (m, no DEPT, -*Ph*), 117.6 (m, DEPT +, BArF_24_), 116.3 (dd, *J* = 21.9 and 7.5 Hz, DEPT +, -*Ph*), 115.9 (dd, *J* = 21.1 and 7.5 Hz, DEPT +, -*Ph*), 77.1 (d, ^2^*J*_P-C_ = 11.9 Hz, DEPT +, -*C*_5_H_4_), 76.5 (m, no DEPT, -*C_5_*H_4_), 76.2 (d, ^3^*J*_P-C_ = 6.8 Hz, DEPT +, -*C*_5_H_4_), 75.9 (m, no DEPT, -*C_5_*H_4_), 75.6 (d, ^2^*J*_P-C_ = 8.2 Hz, DEPT +, -*C*_5_H_4_), 74.9 (d, ^3^*J*_P-C_ = 5.9 Hz, DEPT +, -*C*_5_H_4_). ^19^F{^1^H} NMR (CD_2_Cl_2_): δ −62.8 (s, BArF_24_), −106.7 (s, -C_6_H_4_*F*). ^31^P{^1^H} NMR (CD_2_Cl_2_): δ 36.5 (dd, ^2^*J*_P-P_ = 18.2 and 6.4 Hz, -*P*Ph_2_), 30.4 (dd, ^2^*J*_P-P_ = 486.7 and 6.4, -*P*(*p*-C_6_H_4_F)_3_), 26.1 (dd, ^2^*J*_P-P_ = 486.7 and 18.2 Hz, -*P*Ph_2_). *Anal*. Calc. for C_84_H_55_BClF_27_FeP_3_Pd: C, 56.92; H, 3.13. Found: C, 57.07; H, 3.01%.

##### [Pd(dfurpf)(P(*p*-C_6_H_4_CF_3_)_3_)Cl][BArF_24_]

The product was isolated as an orange–red solid in 67% yield. ^1^H NMR (CDCl_2_) δ: 7.64 (br s, 8H, BArF_24_), 7.58–7.32 (m, 16H, -C_6_*H*_4_CF_3_ and BArF_24_), 6.96 (br s, 2H, -*furanyl*), 6.47 (br s, 2H, -*furanyl*), 6.19 (br s, 2H, -*furanyl*), 5.94 (br s, 2H, -*furanyl*), 4.45 (m, 4H, -C_5_*H*_4_), 4.22 (m, 4H, -C_5_*H*_4_), 2.33 (s, 6H, -*Me*), 2.08 (s, 6H, -*Me*). ^13^C{^1^H} NMR (CD_2_Cl_2_): δ 161.7 (q, ^1^*J*_B-C_ = 49.8 Hz, no DEPT, BArF_24_), 160.3 (d, ^1^*J*_P-C_ = 6.1 Hz, no DEPT, *-furanyl*), 159.9 (d, ^1^*J*_P-C_ = 6.5 Hz, no DEPT, -*furanyl*), 139.6 (m, DEPT +, BArF_24_), 135.0 (d, ^2^*J*_P-C_ = 11.4 Hz, DEPT +, -*Ph*), 134.2 (s, DEPT +, -*furanyl*), 134.0 (s, DEPT +, -*furanyl*), 133.5 (s, DEPT +, -*furanyl*), 132.6 (d, ^1^*J*_P-C_ = 48.3 Ha, no DEPT, -*Ph*), 131.2 (q, ^1^*J*_F-C_ = 32.51 Hz, no DEPT, -*C*F_3_), 128.8 (m, no DEPT, BArF_24_), 126.5 (d, ^3^*J*_P-C_ = 16.4 Hz, DEPT +, -*Ph*), 126.3 (s, DEPT +, -*furanyl*), 126.1 (s, DEPT +, -*furanyl*), 125.6 (m, DEPT +, -*furanyl*), 124.6 (s, DEPT +, -*Ph*), 124.6 (q, ^1^*J*_F-C_ = 272.4 Hz, no DEPT, BArF_24_), 122.9 (d, ^1^*J*_P-C_ = 74.3 Hz, no DEPT, -*Ph*), 117.5 (m, DEPT +, BArF_24_), 109.5 (s, DEPT +, -*furanyl*), 109.4 (s, DEPT +, -*furanyl*), 108.7 (s, DEPT +, -*furanyl*), 108.7 (s, DEPT +, -*furanyl*), 75.9 (d, ^2^*J*_P-C_ = 12.7 Hz, DEPT +, -*C_5_*H_4_), 75.7 (d, ^2^*J*_P-C_ = 14.0 Hz, DEPT +, -*C_5_*H_4_), 75.2 (d, ^3^*J*_P-C_ = 9.5 Hz, DEPT +, -*C_5_*H_4_), 75.1 (d, ^3^*J*_P-C_ = 8.1 Hz, DEPT +, -*C_5_*H_4_), 72.4 (m, no DEPT, -*C*_5_H_4_), 69.2 (m, no DEPT, -*C_5_*H_4_), 13.9 (s, DEPT +, furanyl-*C*H_3_), 13.5 (s, DEPT +, -furanyl-*C*H_3_). ^19^F{^1^H} NMR (CD_2_Cl_2_): δ −62.8 (s, BArF_24_), −63.7 (s, -C*F*_3_). ^19^F{^1^H} NMR (CD_2_Cl_2_): δ −62.8 (s). ^31^P{^1^H} NMR (CD_2_Cl_2_): δ 28.0 (dd, ^2^*J*_P-P_ = 495.2 and 19.6, -*P*(*p*-C_6_H_4_CF_3_)_3_), −5.2 (dd, ^2^*J*_P-P_ = 19.6 and 6.0 Hz, -*P*(furanyl)_2_), −6.4 (dd, ^2^*J*_P-P_ = 495.2 and 6.0 Hz, -*P*(furanyl)_2_). *Anal*. Calc. for C_83_H_52_BClF_33_FeO_4_P_3_Pd: C, 51.51; H, 2.71. Found: C, 51.14; H, 2.94%.

### 3.3. Electrochemical Methods

Cyclic voltammetry was performed using a CH Instruments Model CHI620D potentiostat. The experiments took place under an argon atmosphere in 10.0 mL of CH_2_Cl_2_ at a temperature of 21 ± 1 °C. The supporting electrolyte, [NBu_4_][PF_6_], was 0.1 M, and initial background scans were conducted prior to introducing the analyte, which was present at a concentration of 1.0 mM. A non-aqueous Ag/AgCl reference electrode, separated from the solution by a porous frit, was employed. The working electrode consisted of a glassy carbon electrode, polished with 1.0 μm and 0.25 μm diamond paste and rinsed with CH_2_Cl_2_ before use. The counter–electrode was a Pt wire. Data acquisition occurred at scan rates ranging from 100 to 1000 mV/s in 100 mV increments, with all reported data at 100 mV/s; background subtraction was employed. Following each experiment, the internal reference FcH^0/+^ was introduced at the same concentration as the analyte.

### 3.4. X-ray Crystallography

Crystals of all compounds were grown by vapor diffusion of diethyl ether into a solution of the corresponding compound in CH_2_Cl_2_. The single crystal X-ray diffraction studies were carried out on a Rigaku XtaLab Mini II (Tokyo, Japan) diffractometer equipped with Mo *K*_α_ radiation (*λ* = 0.71073 A°) and a HyPixBantam (Rigaku/Oxford Diffraction, Tokyo, Japan) hybrid photon–counting (HPC) detector. The crystal was mounted on a MiTeGen Micromount with paratone oil. Data were collected in a nitrogen gas stream at 100(2) K using *ϕ* and *ω* scans. Additional experimental parameters can be found in the supporting information. Data collection strategies and data processing were performed using CrysAlis^Pro^ (v43, Rigaku/Oxford Diffraction, Tokyo, Japan) [60]. The data were integrated and scaled within the Rigaku CrysAlis^Pro^ software package. Structure solution and refinement were carried out within the Olex2-1.5 software package [61]. Solution by direct methods (SHELXT [62]) produced a complete phasing model for refinement. All nonhydrogen atoms were refined anisotropically using full-matrix least-squares on *F*^2^ (SHELXL-2014 [63]). Hydrogen atoms were placed using a riding model, with positions constrained relative to their parent atom using the appropriate HFIX command in SHELXL-2014.

## 4. Conclusions

The addition of monodentate phosphines to the dimeric species, [Pd_2_(PP)_2_(*μ*-Cl)_2_][BArF_24_]_2_ (PP = 1,1′-bis(phosphino)ferrocene ligands) typically results in cleavage of the dimer and the formation of [Pd(PP)(PR_3_)Cl][BArF_24_] compounds. These reactions are readily observed visually be the solutions changing color from green–brown to orange–red. The ^31^P{^1^H} NMR spectra confirm the presence of three inequivalent phosphorus atoms with a strong *trans*-coupling of approximately 500 Hz noted for two of the signals. The magnitude of these coupling constants varies based on the PP ligands, with dfurpf > dppf > dippf > dcpf > dppdtbpf. With especially bulky PP or PR_3_ ligands, no reaction was observed. The structures of several of these new compounds provide insight into how the conformational flexibility of the 1,1′-bis(phosphino)ferrocene ligands allows for binding of the monodentate phosphines. The oxidative electrochemistry of most of the [Pd(PP)(PR_3_)Cl][BArF_24_] compounds show one reversible oxidation. The potential at which the oxidation occurs shows dependence on the phosphorus substituents on the 1,1′-bis(phosphino)ferrocene ligands, but the range of potentials, 0.10 V, is less than was observed for the analogous [Pd(PP)Cl_2_] compounds, 0.14 V. The potential at which oxidation of these compounds occurs shows a dependence on the PR_3_ ligand, but with a somewhat limited range of 0.07 V for a variety of different monodentate phosphines, suggesting minimal communication between the iron center of the 1,1′-bis(phosphino)ferrocene ligand and the monodentate phosphine. This is especially apparent in the [Pd(PP)(PPh_2_Fc)Cl][BArF_24_] compounds, which display two oxidative waves. The wave at less positive potentials is attributed to the PPh_2_Fc ligand, while the wave at higher potentials is attributed to the PP ligand. This second wave is formally due to the oxidation of [Pd(PP)(PPh_2_Fc^+^)Cl][BArF_24_], yet the presence of the PPh_2_Fc^+^ ligand does not significantly impact the potential at which oxidation of the PP ligand occurs with respect to other PR_3_ ligands.

## Figures and Tables

**Table 1:** Structural parameters for [Pd_2_(dppdtbpf)_2_(*μ*-Cl)_2_][BArF_24_]_2_.

	[Pd(dppdtbpf)Cl_2_] [42]	[Pd_2_(dppdtbpf)_2_(μ-Cl)_2_][BArF_24_]_2_
P*_t_*_Bu_–Pd, Å	2.3183(9)	2.2977(7)
P_Ph_–Pd, Å	2.2917(9)	2.2917(6)
Pd–Cl (*trans*- to Ph), Å	2.3395(10)	2.3620(8)
Pd–Cl (*cis*- to Ph), Å	2.3416(9)	2.3794(9)
P–Pd–P, °	101.37(3)	99.35(3)
Cl–Pd–Cl, °	85.64(3)	81.04(3)
*τ*_4_ ^a^	0.14	0.14
*τ*′_4_ ^b^	0.12	0.12
Cent–Fe–Cent, °	179.08(10)	177.60(6)
C–Cent–Cent–C, ° ^c^	23.8(2)	19.25(17)
*θ*, ° ^d^	3.05(14)	4.42(10)
%*V*_bur_	58.3	60.0

^a^ Four-coordinate geometry index, where *τ*_4_ = 0.00 is square planar and *τ*_4_ = 1.00 is tetrahedral [44]. ^b^ Modified four-coordinate geometry index, where *τ*′_4_ = 0.00 is square planar and *τ*′_4_ = 1.00 is tetrahedral [45]. ^c^ The torsion angle formed between C_A_-Cent_A_-Cent_B_-C_B_, with C being the carbon atom bonded to the phosphorus and Cent being the centroid of the C_5_ ring. ^d^ The dihedral angle between the two C_5_ rings.

**Table 2:** ^31^P{^1^H} NMR data for [Pd(PP)(PR_3_)Cl][BArF_24_] compounds.

PP	PR_3_	^31^P NMR Signal in ppm (Coupling Constant in Hz)		
dppf ^a^	PMe_3_	36.1 (19.8 and 4.7)	32.8 (471.1 and 4.7)	−5.3 (471.1 and 19.8)
dippf	PMe_3_	67.7 (31.2 and 12.0)	57.9 (446.1 and 12.0)	−11.0 (446.1 and 31.2)
dppdtbpf	PMe_3_	74.0 (430.7 and 5.5)	43.5 (23.5 and 5.5)	−8.0 (430.7 and 23.5)
dcpf	PMe_3_	60.6 (30.4 and 13.6)	50.2 (443.0 and 13.6)	−11.0 (443.0 and 30.4)
dfurpf	PMe_3_	31.4 (493.0 and 15.7)	−5.9 (15.7 and 12.9)	−9.4 (493.0 and 12.9)
dppf ^a^	PPh_3_	35.5 (17.6 and 4.9)	31.0 (480.6 and 17.6)	27.1 (480.6 and 4.9)
dippf	PPh_3_	65.5 (24.0 and 14.1)	62.5 (428.1 and 14.1)	23.7 (428.1 and 24.0)
dppdtbpf	PPh_3_	78.8 (423.0 and 6.2)	44.1 (19.6 and 6.2)	24.2 (423.0 and 19.6)
dfurpf	PPh_3_	31.8 (491.0 and 17.1)	−5.8 (17.1 and 11.7)	−9.0 (491.0 and 11.7)
dppf ^a^	PPh_2_Fc	36.4 (20.0 and 5.4)	30.3 (480.8 and 20.0)	24.4 (480.4 and 5.4)
dippf	PPh_2_Fc	64.1 (23.5 and 9.7)	58.8 (434.6 and 9.7)	20.0 (434.6 and 23.5)
dppdtbpf	PPh_2_Fc	74.4 (429.6 and 18.1)	43.0 (18.1 and 6.1)	22.8 (429.6 and 18.1)
dfurpf	PPh_2_Fc	30.1 (500.8 and 17.3)	−5.9 (17.6 and 15.6)	−10.6 (500.8 and 17.3)
dppf	P(NMe_2_)_3_	94.3 (607.0 and 10.0)	32.2 (17.5 and 9.9)	19.0 (607.0 and 17.5)
dppf	P*^i^*Pr_3_	50.6 (450.0 and 17.6)	32.7 (17.6 and 5.9)	21.6 (450.0 and 5.9)
dppf	P(CH_2_Ph)_3_	34.0 (16.0 and 6.5)	30.1 (452.4 and 6.5)	18.9 (452.4 and 16.0)
dppf	P(*m*-tol)_3_	35.2 (17.5 and 5.8)	30.2 (478.7 and 17.5)	26.5 (478.7 and 5.8)
dppf	P(*p*-tol)_3_	35.2 (17.6 and 5.9)	30.3 (479.3 and 17.6)	26.5 (479.3 and 5.9)
dppf	P(*p*-C_6_H_4_OMe)_3_	50.5 (451.9 and 17.7)	32.6 (17.7 and 6.3)	21.6 (451.9 and 6.3)
dppf	P(*p*-C_6_H_4_F)_3_	36.5 (18.2 and 6.4)	30.4 (486.7 and 6.4)	26.1 (486.7 and 18.2)
dfurpf	P(*p*-C_6_H_4_CF_3_)_3_	28.0 (495.2 and 19.6)	−5.2 (19.6 and 6.0)	−6.4 (495.2 and 6.0)

^a^ Reference [37].

**Table 3:** Structural parameters for [Pd(PP)(PR_3_)Cl)][BArF_24_] compounds where R = alkyl.

	[Pd(PP)(PR_3_)Cl][BArF_24_]			
PP	dippf	dcpf	dppf [37]	dppf
PR_3_	PMe_3_	PMe_3_	PMe_3_	P*^i^*Pr_3_
Pd–P (*trans*-PR_3_), Å	2.3728(7)	2.3815(12)	2.3666(15)	2.4211(7)
Pd–P (*cis*-PR_3_), Å	2.2902(7)	2.3039(13)	2.2833(14)	2.2981(6)
Pd–PR_3_, Å	2.3908(9)	2.3915(14)	2.3722(15)	2.3767(6)
Pd–Cl, Å	2.3367(7)	2.3467(13)	2.3324(14)	2.3378(6)
P_PP_–Pd–P_PP_, °	100.25(3)	100.30(5)	98.81(5)	93.01(2)
Cl–Pd–PR_3_, °	78.74(3)	80.28(5)	82.83(5)	85.031
*τ*_4_ ^a^	0.23	0.19	0.12	0.19
*τ*′_4_ ^b^	0.20	0.15	0.11	0.17
Cent–Fe–Cent, °	178.40(7)	178.27(13)	179.03(5)	179.23(5)
C–Cent–Cent–C, ° ^c^	21.96(19)	24.4(3)	40.2(4)	36.49(12)
*θ*, ° ^d^	3.46(11)	4.4(2)	4.4(2)	4.90(9)
%*V*_bur_ (PP)	56.6	55.0	55.1	52.1
%*V*_bur_ (PR_3_)	21.6	21.5	22.1	29.1

^a^ Four-coordinate geometry index, where *τ*_4_ = 0.00 is square planar and *τ*_4_ = 1.00 is tetrahedral [44]. ^b^ Modified four-coordinate geometry index, where *τ*′_4_ = 0.00 is square planar and *τ*′_4_ = 1.00 is tetrahedral [45]. ^c^ The torsion angle formed between C_A_–Cent_A_–Cent_B_–C_B_, with C being the carbon atom bonded to the phosphorus and Cent being the centroid of the C_5_ ring. ^d^ The dihedral angle between the two C_5_ rings.

**Table 4:** Structural parameters for [Pd(PP)(PR_3_)Cl)][BArF_24_] compounds where R = aryl.

	[Pd(PP)(PR_3_)Cl][BArF_24_]		
PP	dppf	dppf [37]	dfurpf
PR_3_	P(*p*-C_6_H_4_F)_3_	PPh_3_	P(*p*-C_6_H_4_CF_3_)_3_
Pd–P (*trans*-PR_3_), Å	2.3449(13)	2.3517(7)	2.3686(17)
Pd–P (*cis*-PR_3_), Å	2.2781(14)	2.2803(6)	2.2651(15)
Pd–PR_3_, Å	2.3786(13)	2.3822(7)	2.3516(17)
Pd–Cl, Å	2.3435(15)	2.3435(6)	2.3301(14)
P_PP_–Pd–P_PP_, °	98.99(5)	99.74(2)	95.28(6)
Cl–Pd–PR_3_, °	83.36(5)	82.90(2)	88.65(5)
*τ*_4_ ^a^	0.21	0.19	0.22
*τ*′_4_ ^b^	0.18	0.16	0.20
Cent–Fe–Cent, °	178.46(15)	178.70(3)	179.27(14)
C–Cent–Cent–C, ° ^c^	37.4(5)	38.32(15)	26.6(4)
*θ*, ° ^d^	6.00(3)	5.59(11)	3.8(3)
%*V*_bur_ (PP)	56.1	55.4	51.3
%*V*_bur_ (PR_3_)	27.9	27.9	28.7

^a^ Four-coordinate geometry index, where *τ*_4_ = 0.00 is square planar and *τ*_4_ = 1.00 is tetrahedral [44]. ^b^ Modified four-coordinate geometry index, where *τ*′_4_ = 0.00 is square planar and *τ*′_4_ = 1.00 is tetrahedral [45]. ^c^ The torsion angle formed between C_A_–Cent_A_–Cent_B_–C_B_ with C being the carbon atom bonded to the phosphorus and Cent being the centroid of the C_5_ ring. ^d^ The dihedral angle between the two C_5_ rings.

**Table 5:** Cyclic voltammetry (*E* in V vs. FcH^0/+^) data for [Pd(PP)(PR_3_)Cl][BArF_24_] with similar PR_3_ ligands in CH_2_Cl_2_.

	dppf	dippf	dppdtbpf	Dcpf	dfurpf
[Pd(PP)Cl_2_]	0.57 [39]	0.43 [40]	0.51 [39]	0.47 [41]	0.55 [38]
[Pd(PP)(PMe_3_)Cl][BArF_24_]	0.66 [37]	0.58	0.59	0.56	0.64
[Pd(PP)(PPh_3_)Cl][BArF_24_]	0.69 [37]	0.59	0.58		0.62
[Pd(PP)(PPh_2_Fc)Cl[BArF_24]_	0.23 [37]	0.22	0.24		0.24
	0.68 [37]	0.64	0.63		0.65

**Table 6:** Cyclic voltammetry data for [Pd(PP)(PR_3_)Cl][BArF_24_] with various PR_3_ ligands in CH_2_Cl_2_.

	[Pd(dppf)(PR_3_)Cl][BArF_24_]	[Pd(dfurpf)(PR_3_)Cl][BArF_24_]
PMe_3_	0.66 [37]	0.64
P(NMe_2_)_3_	0.63	
P^i^Pr_3_	0.65	
P(CH_2_Ph)_3_	0.68	
PPh_3_	0.69 [37]	0.62
P(*m*-tol)_3_	0.65	
P(*p*-tol)_3_	0.64	
P(*p*-C_6_H_4_OMe)_3_	0.62	
P(*p*-C_6_H_4_F)_3_	0.65	
P(*p*-C_6_H_4_CF_3_)_3_		0.59

## Data Availability

Data are contained within the article and Supplementary Materials.

## Supplementary Materials

The following supporting information can be downloaded at: https://www.mdpi.com/article/10.3390/molecules29092047/s1: CCDC 2340750-2340755 contains the supplementary crystallographic data for the [Pd_2_(dppdtbpf)_2_(μ-Cl)_2_][BArF_24_]_2_ and [Pd(PP)(PR_3_)Cl][BArF_24_] (PP = dppf, PR_3_ = P*^i^*Pr_3_ or P(*p*-C_6_H_4_F)_3_; PP = dippf or dcpf, PR_3_ = PMe_3_; PP = dfurpf, PR_3_ = P(*p*-C_6_H_4_CF_3_)_3_) compounds. These data can be obtained free of charge via http://www.ccdc.cam.ac.uk/conts/retrieving.html, or from the Cambridge Crystallographic Data Centre, 12 Union Road, Cambridge CB2 1EZ, UK; fax: (+44) 1223-336-033; or e-mail: deposit@ccdc.cam.ac.uk. The following supporting information can be downloaded: ^31^P{1H} NMR spectra, crystallographic experimental details, %*V*_bur_ results, and CV scans for all compounds.
