# Supplementary material for: Cleavage of [Pd2(PP)2(μ-Cl)2][BArF24]2 (PP = Bis(phosphino)ferrocene, BArF24 = Tetrakis(3,5-bis(trifluoromethyl)phenyl)borate) with Monodentate Phosphines
Source: Molecules. 2024 Apr 29;29(9):2047. doi: 10.3390/molecules29092047 (PMC11085644; doi:10.3390/molecules29092047)
Supplement: Supplementary file 1 [file molecules-29-02047-s001.zip › molecules-2956794-supplementary.pdf]

Supporting Information

Fig.	Table		Pg.
S1		$^{31}P\{^1H\}$ NMR spectrum of $[Pd_2(dfurpf)_2(\mu\text{-}Cl)_2][BArF_{24}]_2$ in CD_2Cl_2.	S4
S2		$^{31}P\{^1H\}$ NMR spectrum of $[Pd(dippf)(PMe_3)Cl][BArF_{24}]$ in CD_2Cl_2.	S5
S3		$^{31}P\{^1H\}$ NMR spectrum of $[Pd(dippf)(PPh_3)Cl][BArF_{24}]$ in CD_2Cl_2.	S6
S4		$^{31}P\{^1H\}$ NMR spectrum of $[Pd(dippf)(PPh_2Fc)Cl][BArF_{24}]$ in CD_2Cl_2.	S7
S5		$^{31}P\{^1H\}$ NMR spectrum of $[Pd(dppdtbpf)(PMe_3)Cl][BArF_{24}]$ in CD_2Cl_2.	S8
S6		$^{31}P\{^1H\}$ NMR spectrum of $[Pd(dppdtbpf)(PPh_2Fc)Cl][BArF_{24}]$ in CD_2Cl_2.	S9
S7		$^{31}P\{^1H\}$ NMR spectrum of $[Pd(dcpf)(PMe_3)Cl][BArF_{24}]$ in CD_2Cl_2.	S10
S8		$^{31}P\{^1H\}$ NMR spectrum of $[Pd(dfurpf)(PMe_3)Cl][BArF_{24}]$ in $CDCl_3$.	S11
S9		$^{31}P\{^1H\}$ NMR spectrum of $[Pd(dfurpf)(PPh_3)Cl][BArF_{24}]$ in CD_2Cl_2.	S12
S10		$^{31}P\{^1H\}$ NMR spectrum of $[Pd(dfurpf)(PPh_2Fc)Cl][BArF_{24}]$ in CD_2Cl_2.	S13
S11		$^{31}P\{^1H\}$ NMR spectrum of $[Pd(dppf)(P(NMe_2)_3)Cl][BArF_{24}]$ in CD_2Cl_2.	S14
S12		$^{31}P\{^1H\}$ NMR spectrum of $[Pd(dppf)(P^iPr_3)Cl][BArF_{24}]$ in CD_2Cl_2.	S15
S13		$^{31}P\{^1H\}$ NMR spectrum of $[Pd(dppf)(P(CH_2Ph)_3)Cl][BArF_{24}]$ in CD_2Cl_2.	S16
S14		$^{31}P\{^1H\}$ NMR spectrum of $[Pd(dppf)(P(m\text{-}tol)_3)Cl][BArF_{24}]$ in CD_2Cl_2.	S17
S15		$^{31}P\{^1H\}$ NMR spectrum of $[Pd(dppf)(P(p\text{-}tol)_3)Cl][BArF_{24}]$ in CD_2Cl_2.	S18
S16		$^{31}P\{^1H\}$ NMR spectrum of $[Pd(dppf)(P(p\text{-}C_6H_4OMe)_3)Cl][BArF_{24}]$ in CD_2Cl_2.	S19
S17		$^{31}P\{^1H\}$ NMR spectrum of $[Pd(dppf)(P(p\text{-}C_6H_4F)_3)Cl][BArF_{24}]$ in CD_2Cl_2.	S20
S18		$^{31}P\{^1H\}$ NMR spectrum of $[Pd(dfurpf)(P(p\text{-}C_6H_4CF_3)_3)Cl][BArF_{24}]$ in CD_2Cl_2.	S21
	S1	Crystal data and structure analysis results for $[Pd_2(dppdtbpf)_2(\mu\text{-}Cl)_2][BArF_{24}]_2$.	S22
	S2	Crystal data and structure analysis results for monodentate alkyl phosphines.	S23
	S3	Crystal data and structure analysis results for monodentate aryl phosphines.	S24
S19		$\%V_{bur}$ calculation for the dppdtbpf ligand in $[Pd_2(dppdtbpf)_2(\mu\text{-}Cl)_2][BArF_{24}]_2$.	S25
S20		$\%V_{bur}$ calculation for the dippf ligand in $[Pd(dippf)(PMe_3)Cl][BArF_{24}]$.	S26
S21		$\%V_{bur}$ calculation for the PMe_3 ligand in $[Pd(dippf)(PMe_3)Cl][BArF_{24}]$.	S27
S22		$\%V_{bur}$ calculation for the dcpf ligand in $[Pd(dcpf)(PMe_3)Cl][BArF_{24}]$.	S28
S23		$\%V_{bur}$ calculation for the PMe_3 ligand in $[Pd(dcpf)(PMe_3)Cl][BArF_{24}]$.	S29
S24		$\%V_{bur}$ calculation for the dppf ligand in $[Pd(dppf)(P^iPr_3)Cl][BArF_{24}]$.	S30
S25		$\%V_{bur}$ calculation for the P^iPr_3 ligand in $[Pd(dppf)(P^iPr_3)Cl][BArF_{24}]$.	S31
S26		$\%V_{bur}$ calculation for the dppf ligand in $[Pd(dppf)(P(p\text{-}C_6H_4F)_3)Cl][BArF_{24}]$.	S32
S27		$\%V_{bur}$ calculation for the $P(p\text{-}C_6H_4F)_3$ ligand in $[Pd(dppf)(P(p\text{-}C_6H_4F)_3)Cl][BArF_{24}]$.	S33
S28		$\%V_{bur}$ calculation for the dfurpf ligand in $[Pd(dfurpf)(P(p\text{-}C_6H_4CF_3)_3)Cl][BArF_{24}]$.	S34
S29		$\%V_{bur}$ calculation for the $P(p\text{-}C_6H_4CF_3)_3$ ligand in $[Pd(dfurpf)(P(p\text{-}C_6H_4CF_3)_3)Cl][BArF_{24}]$.	S35
S30		CV scans of 1.0 mM $[Pd_2(dfurpf)_2(\mu\text{-}Cl)_2][BArF_{24}]_2$ with 0.1 M	S36

	$[NBu_4][PF_6]$ as the supporting electrolyte measured at 100 mV s^{-1}.	
S31	CV scan of 1.0 mM $[Pd(dippf)(PMe_3)Cl][BArF_{24}]$ with 0.1 M $[NBu_4][PF_6]$ as the supporting electrolyte measured at 100 mV s^{-1}.	S37
S32	CV scan of 1.0 mM $[Pd(dippf)(PPh_3)Cl][BArF_{24}]$ with 0.1 M $[NBu_4][PF_6]$ as the supporting electrolyte measured at 100 mV s^{-1}.	S38
S33	CV scan of 1.0 mM $[Pd(dippf)(PPh_2Fc)Cl][BArF_{24}]$ with 0.1 M $[NBu_4][PF_6]$ as the supporting electrolyte measured at 100 mV s^{-1}.	S39
S34	CV scan of 1.0 mM $[Pd(dppdtbpf)(PMe_3)Cl][BArF_{24}]$ with 0.1 M $[NBu_4][PF_6]$ as the supporting electrolyte measured at 100 mV s^{-1}.	S40
S35	CV scan of 1.0 mM $[Pd(dppdtbpf)(PPh_3)Cl][BArF_{24}]$ with 0.1 M $[NBu_4][PF_6]$ as the supporting electrolyte measured at 100 mV s^{-1}.	S41
S36	CV scan of 1.0 mM $[Pd(dppdtbpf)(PPh_2Fc)Cl][BArF_{24}]$ with 0.1 M $[NBu_4][PF_6]$ as the supporting electrolyte measured at 100 mV s^{-1}.	S42
S37	CV scan of 1.0 mM $[Pd(dcpf)(PMe_3)Cl][BArF_{24}]$ with 0.1 M $[NBu_4][PF_6]$ as the supporting electrolyte measured at 100 mV s^{-1}.	S43
S38	CV scan of 1.0 mM $[Pd(dfurpf)(PMe_3)Cl][BArF_{24}]$ with 0.1 M $[NBu_4][PF_6]$ as the supporting electrolyte measured at 100 mV s^{-1}.	S44
S39	CV scan of 1.0 mM $[Pd(dfurpf)(PPh_3)Cl][BArF_{24}]$ with 0.1 M $[NBu_4][PF_6]$ as the supporting electrolyte measured at 100 mV s^{-1}.	S45
S40	CV scan of 1.0 mM $[Pd(dppf)(P(NMe_2)_3)Cl][BArF_{24}]$ with 0.1 M $[NBu_4][PF_6]$ as the supporting electrolyte measured at 100 mV s^{-1}.	S46
S41	CV scan of 1.0 mM $[Pd(dppf)(P(CH_2Ph)_3)Cl][BArF_{24}]$ with 0.1 M $[NBu_4][PF_6]$ as the supporting electrolyte measured at 100 mV s^{-1}.	S47
S42	CV scan of 1.0 mM [Pd(dppf)(P(*m*-tol)$_3$)Cl][BArF$_{24}$] with 0.1 M $[NBu_4][PF_6]$ as the supporting electrolyte measured at 100 mV s^{-1}.	S48
S43	CV scan of 1.0 mM [Pd(dppf)(P(*p*-tol)$_3$)Cl][BArF$_{24}$] with 0.1 M $[NBu_4][PF_6]$ as the supporting electrolyte measured at 100 mV s^{-1}.	S49
S44	CV scan of 1.0 mM [Pd(dppf)(P(*p*-C$_6$H$_4$OMe)$_3$)Cl][BArF$_{24}$] with 0.1 M $[NBu_4][PF_6]$ as the supporting electrolyte measured at 100 mV s^{-1}.	S50
S45	CV scan of 1.0 mM [Pd(dppf)(P(*p*-C$_6$H$_4$F)$_3$)Cl][BArF$_{24}$] with 0.1 M $[NBu_4][PF_6]$ as the supporting electrolyte measured at 100 mV s^{-1}.	S51
S46	CV scan of 1.0 mM [Pd(dfurpf)(P(*p*-C$_6$H$_4$CF$_3$)$_3$)Cl][BArF$_{24}$] with 0.1 M $[NBu_4][PF_6]$ as the supporting electrolyte measured at 100 mV s^{-1}.	S52

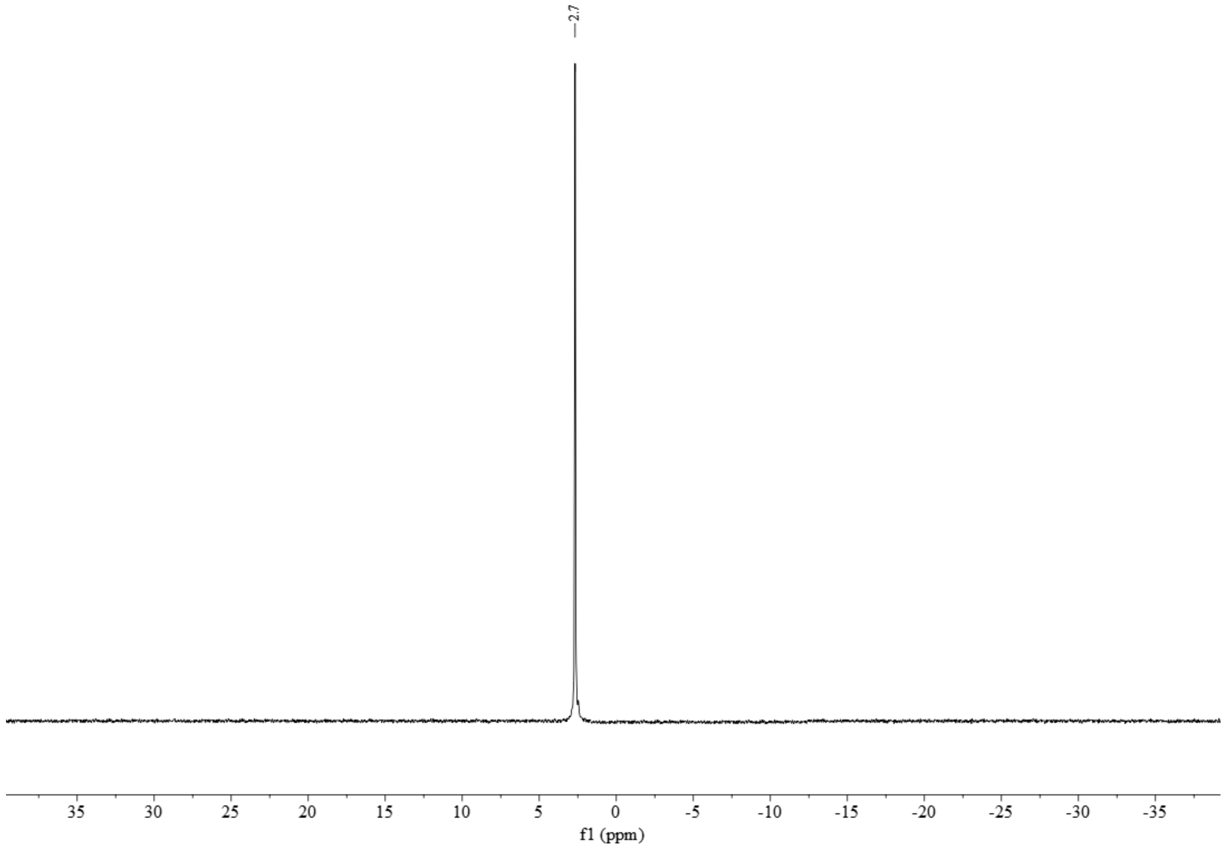

Fig. S1. $^{31}P\{^{1}H\}$ NMR spectrum of $[Pd_2(dfurpf)_2(\mu\text{-}Cl)_2][BArF_{24}]_2$ in CD_2Cl_2.

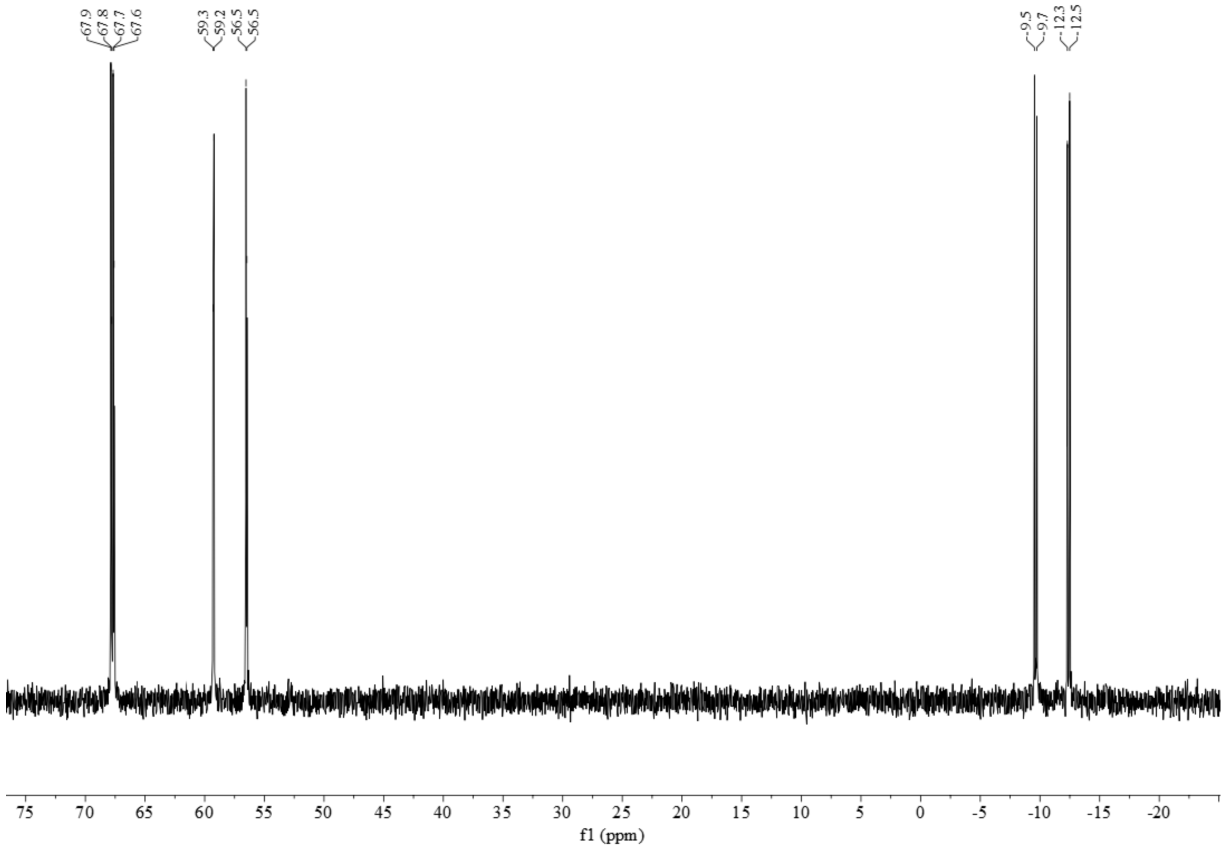

Fig. S2. $^{31}P\{^{1}H\}$ NMR spectrum of $[Pd(dippf)(PMe_3)Cl][BArF_{24}]$ in CD_2Cl_2.

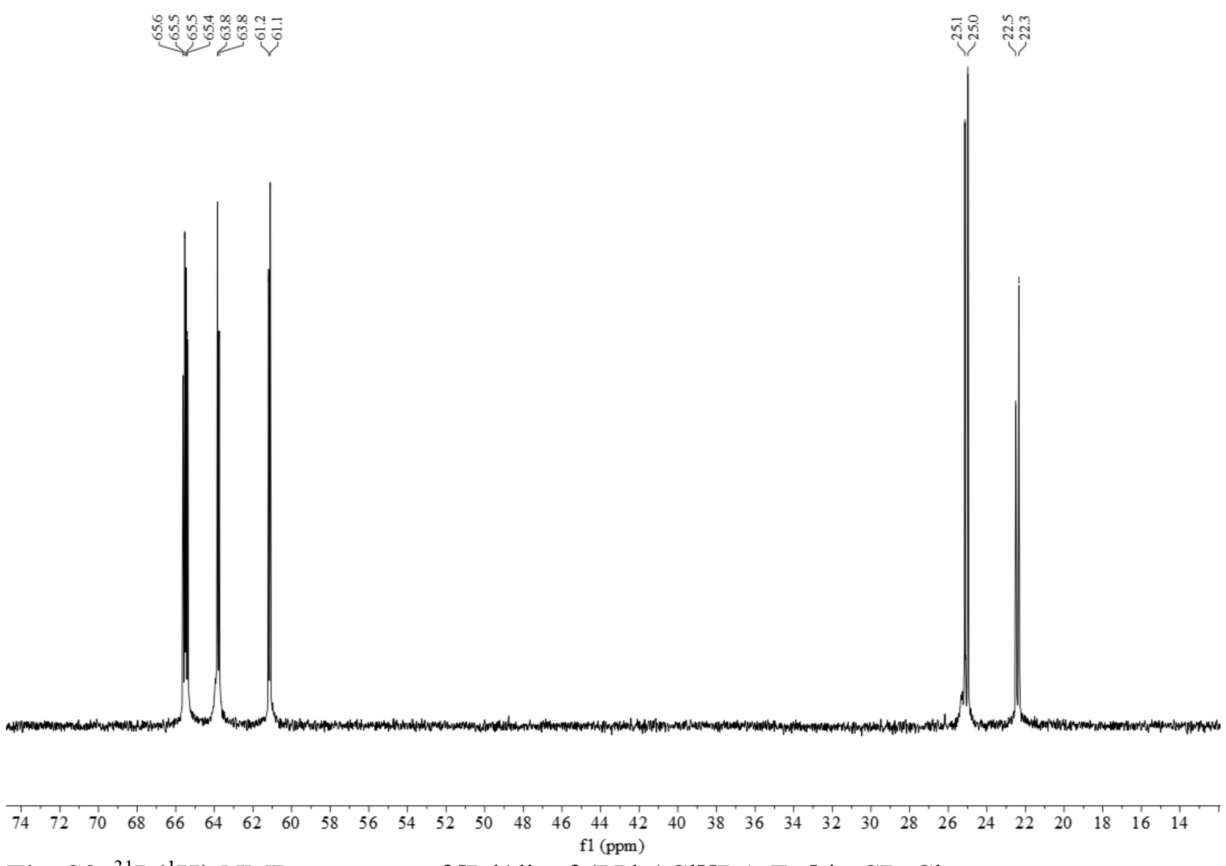

Fig. S3. $^{31}P\{^{1}H\}$ NMR spectrum of [Pd(dippf)(PPh_3)Cl][$BArF_{24}$] in CD_2Cl_2.

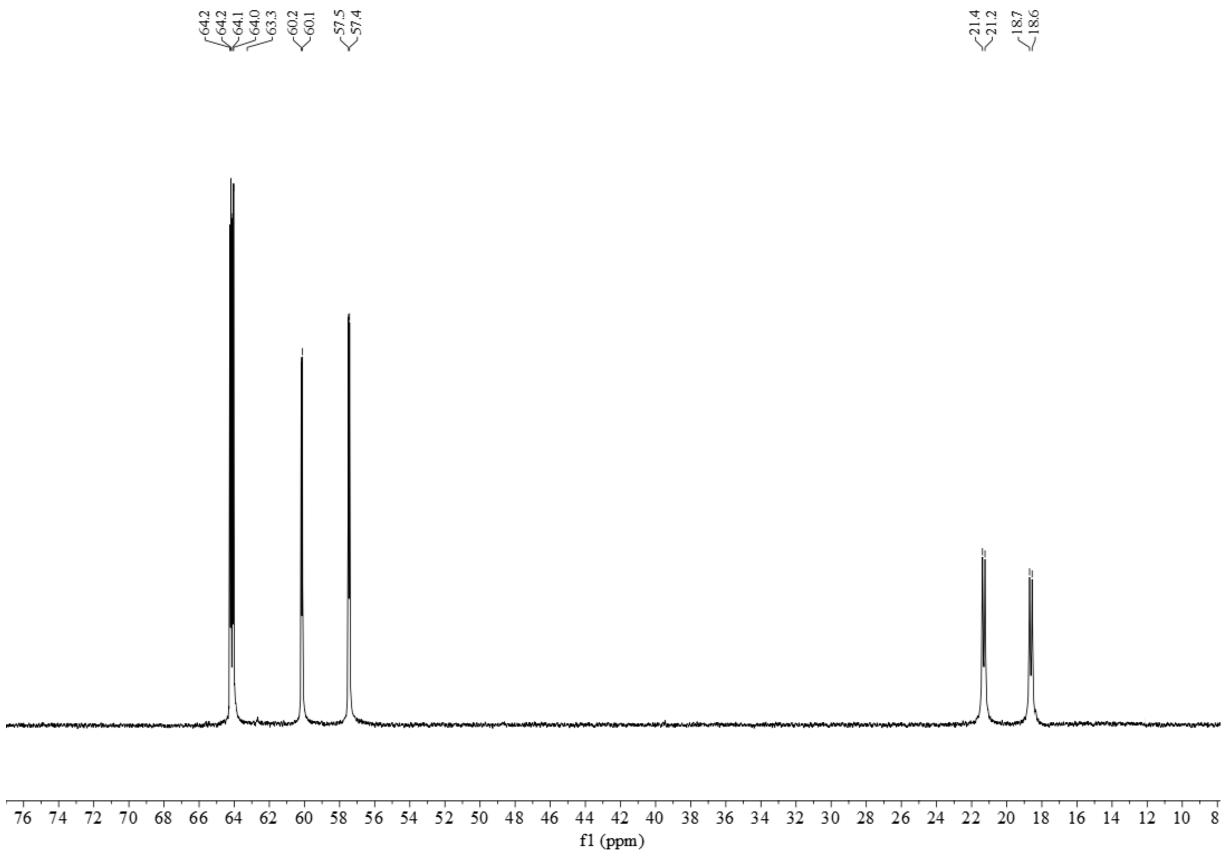

Fig. S4. $^{31}P\{^{1}H\}$ NMR spectrum of $[Pd(dippf)(PPh_2Fc)Cl][BArF_{24}]$ in CD_2Cl_2.

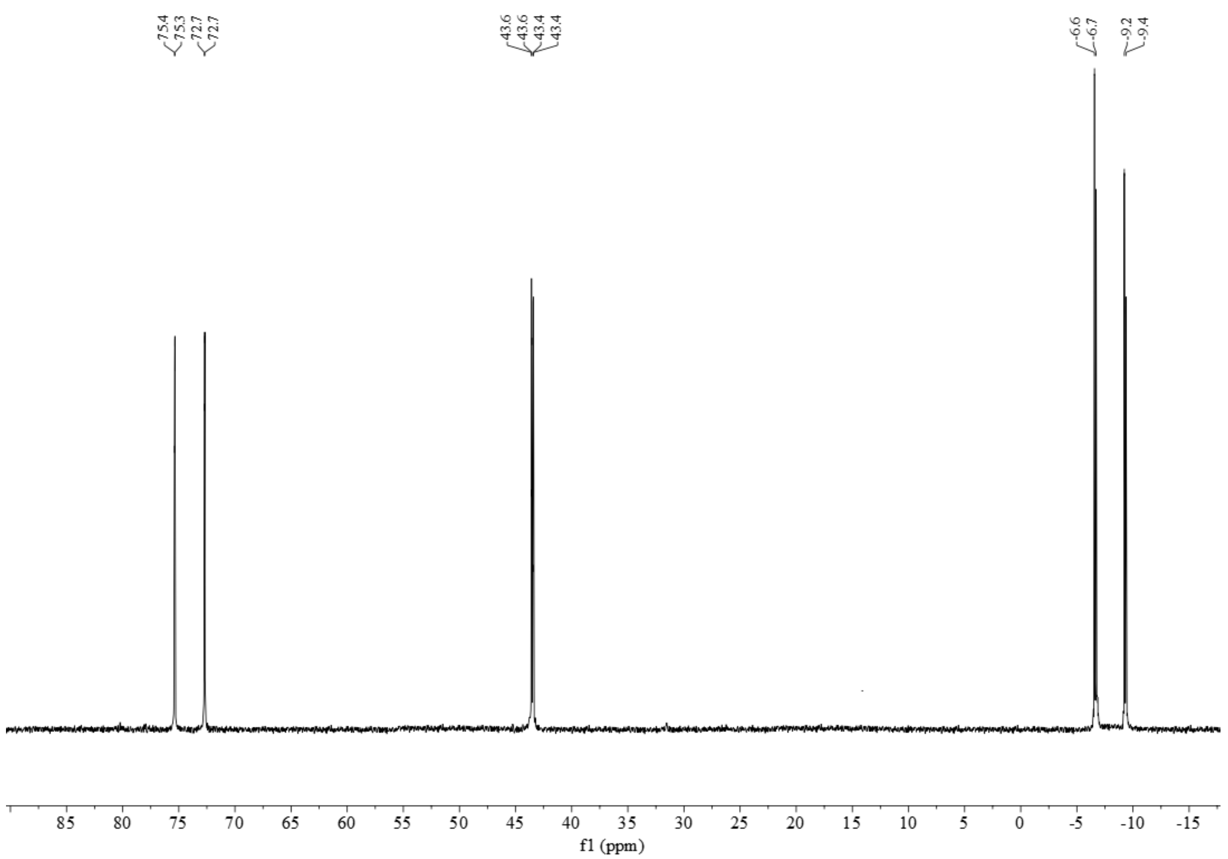

Fig. S5. $^{31}P\{^{1}H\}$ NMR spectrum of [Pd(dppdtbpf)(PMe_3)Cl][$BArF_{24}$] in CD_2Cl_2.

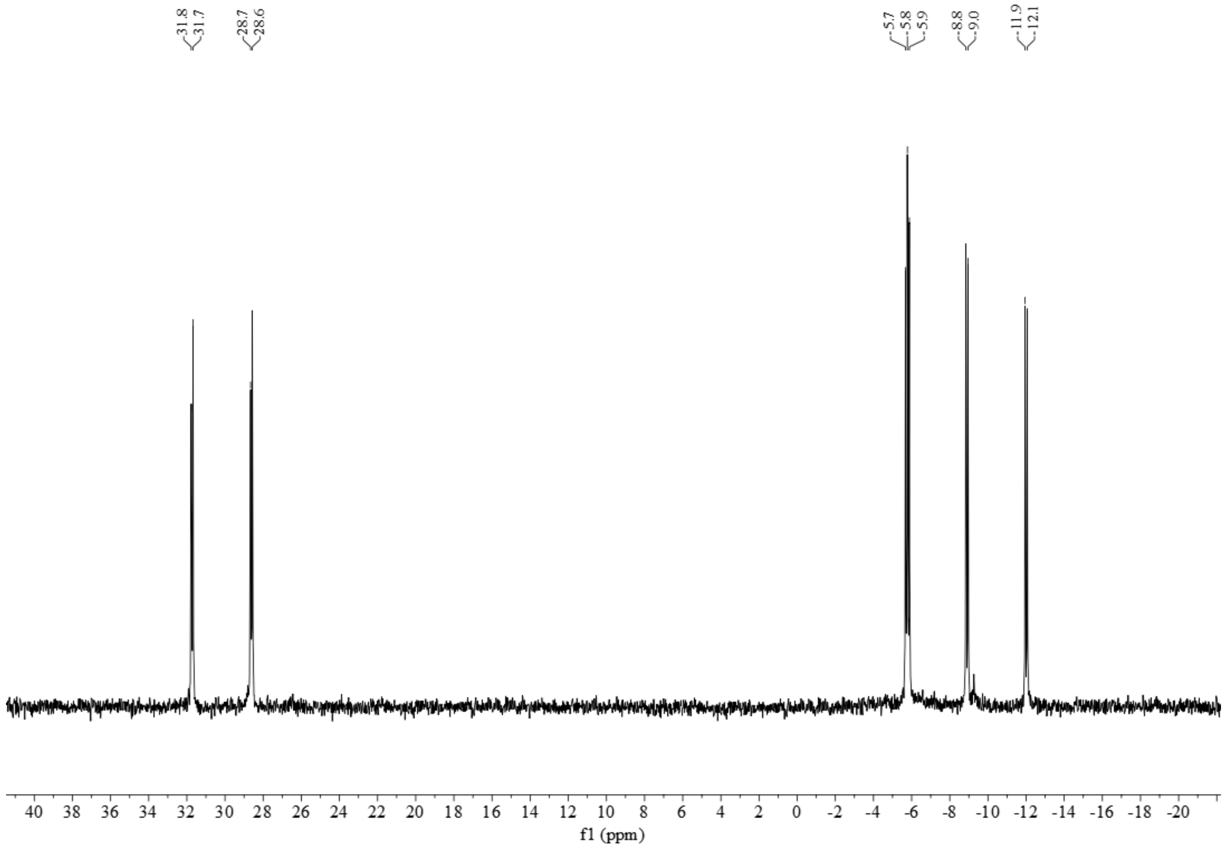

Fig. S6. $^{31}P\{^1H\}$ NMR spectrum of [Pd(dppdtbpf)(PPh_2Fc)Cl][$BArF_{24}$] in CD_2Cl_2.

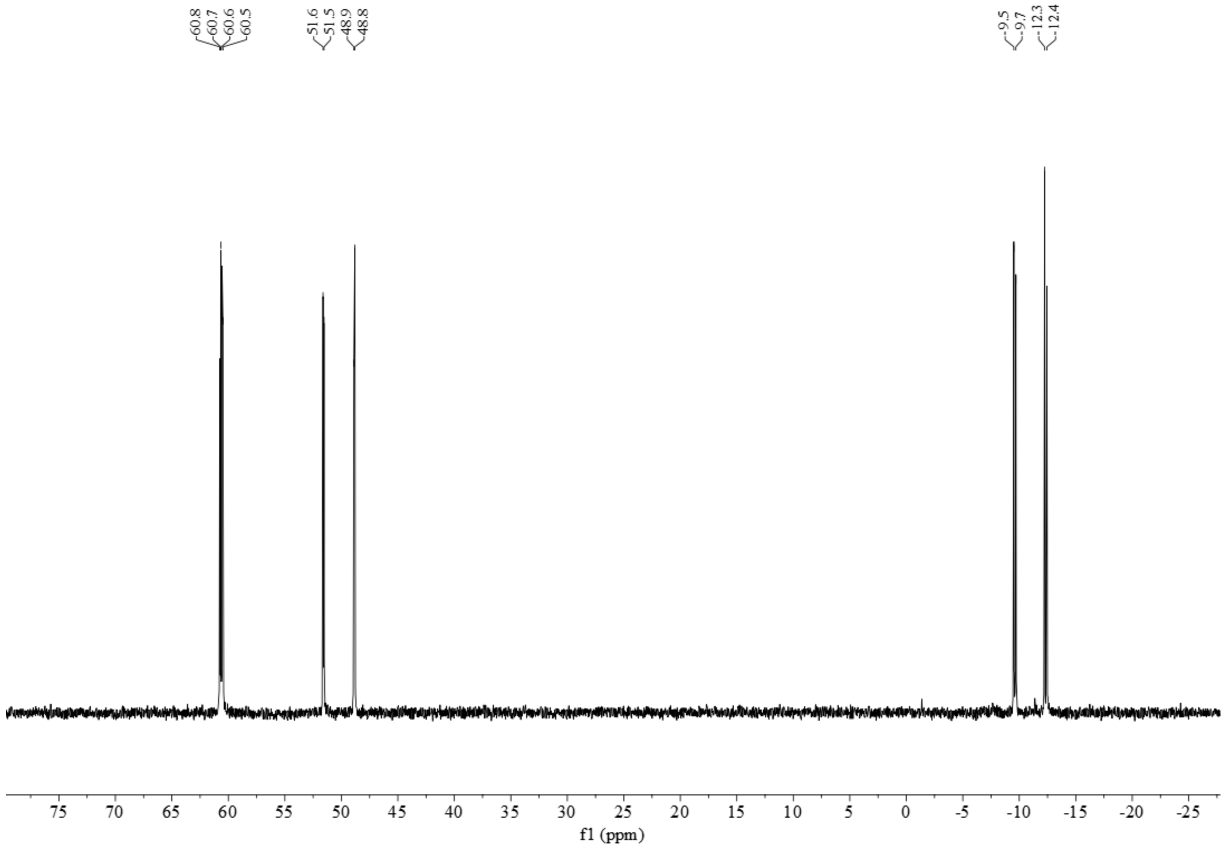

Fig. S7. $^{31}P\{^{1}H\}$ NMR spectrum of [Pd(dcpf)(PMe$_3$)Cl][BArF$_{24}$] in CD_2Cl_2.

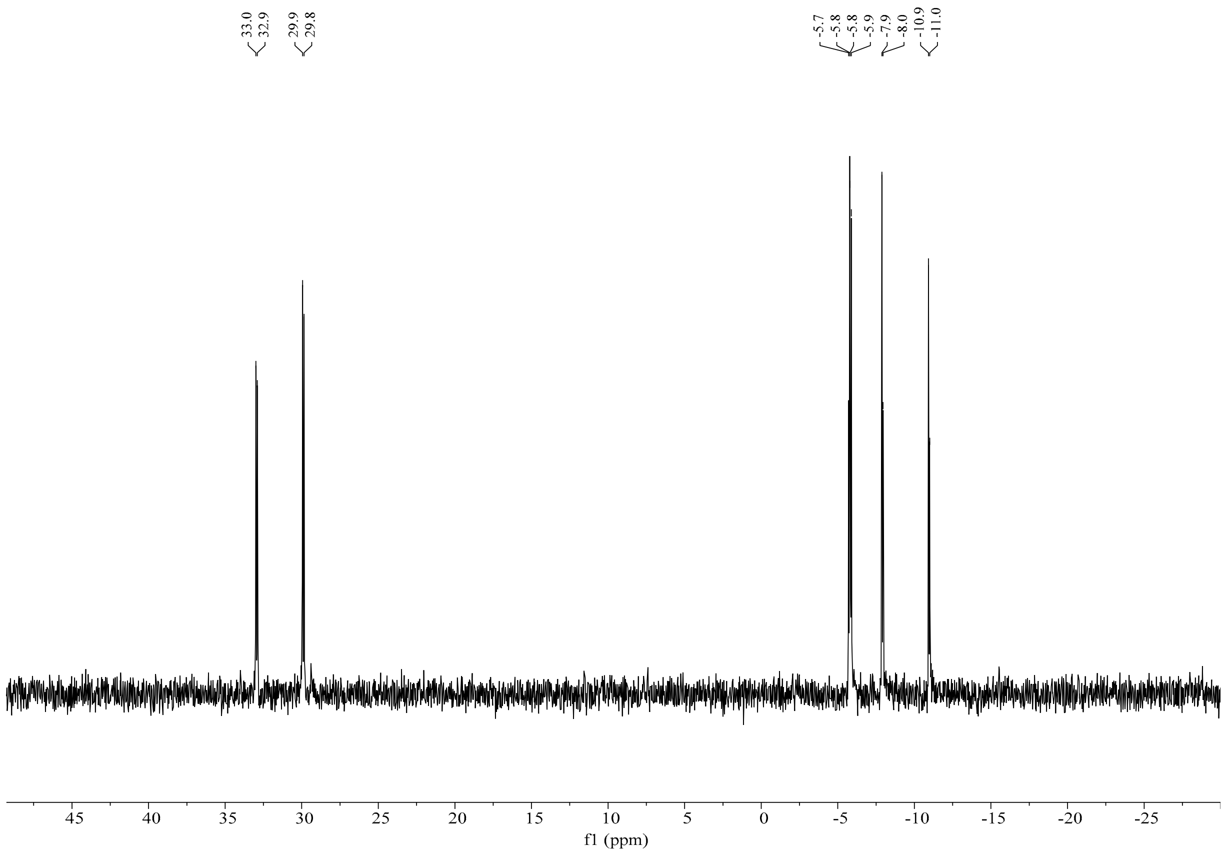

Fig. S8. $^{31}P\{^1H\}$ NMR spectrum of $[Pd(dfurpf)(PMe_3)Cl][BArF_{24}]$ in $CDCl_3$.

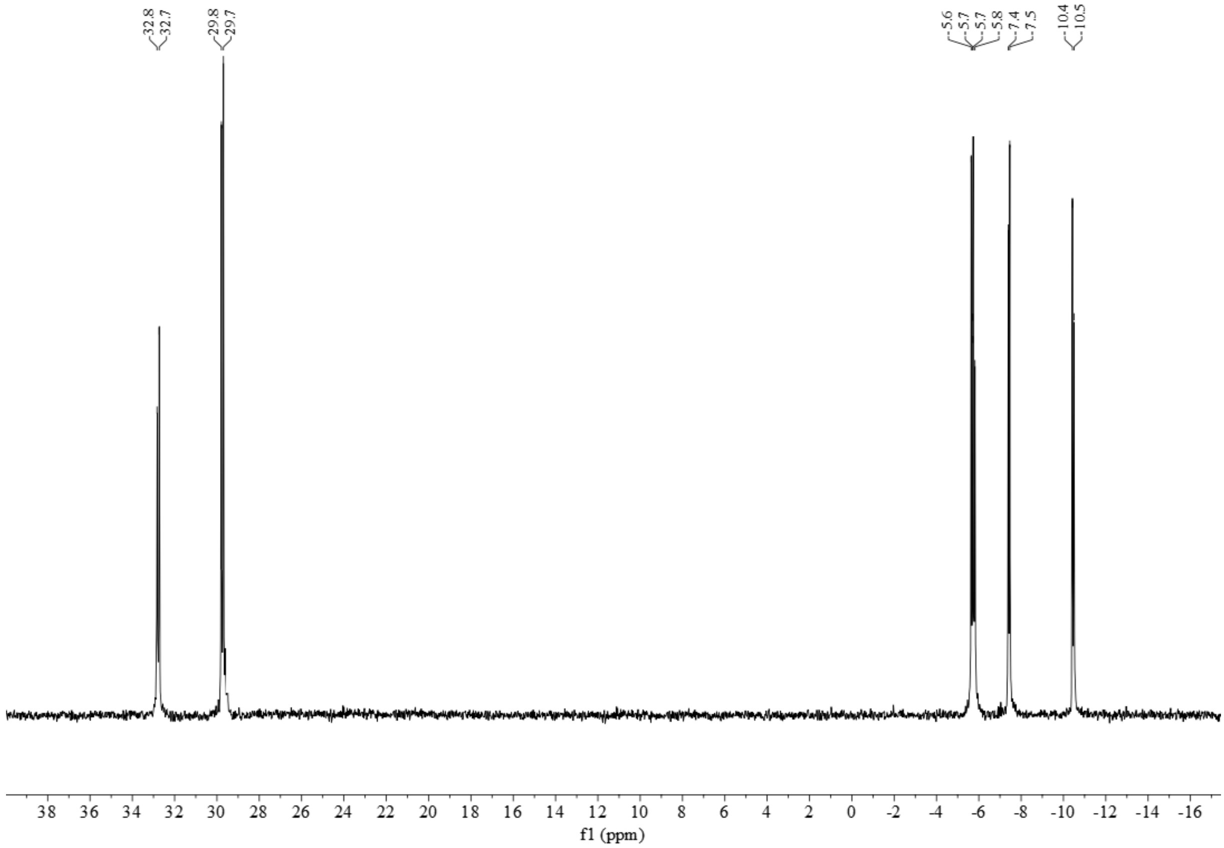

Fig. S9. $^{31}P\{^1H\}$ NMR spectrum of $[Pd(dfurpf)(PPh_3)Cl][BArF_{24}]$ in CD_2Cl_2.

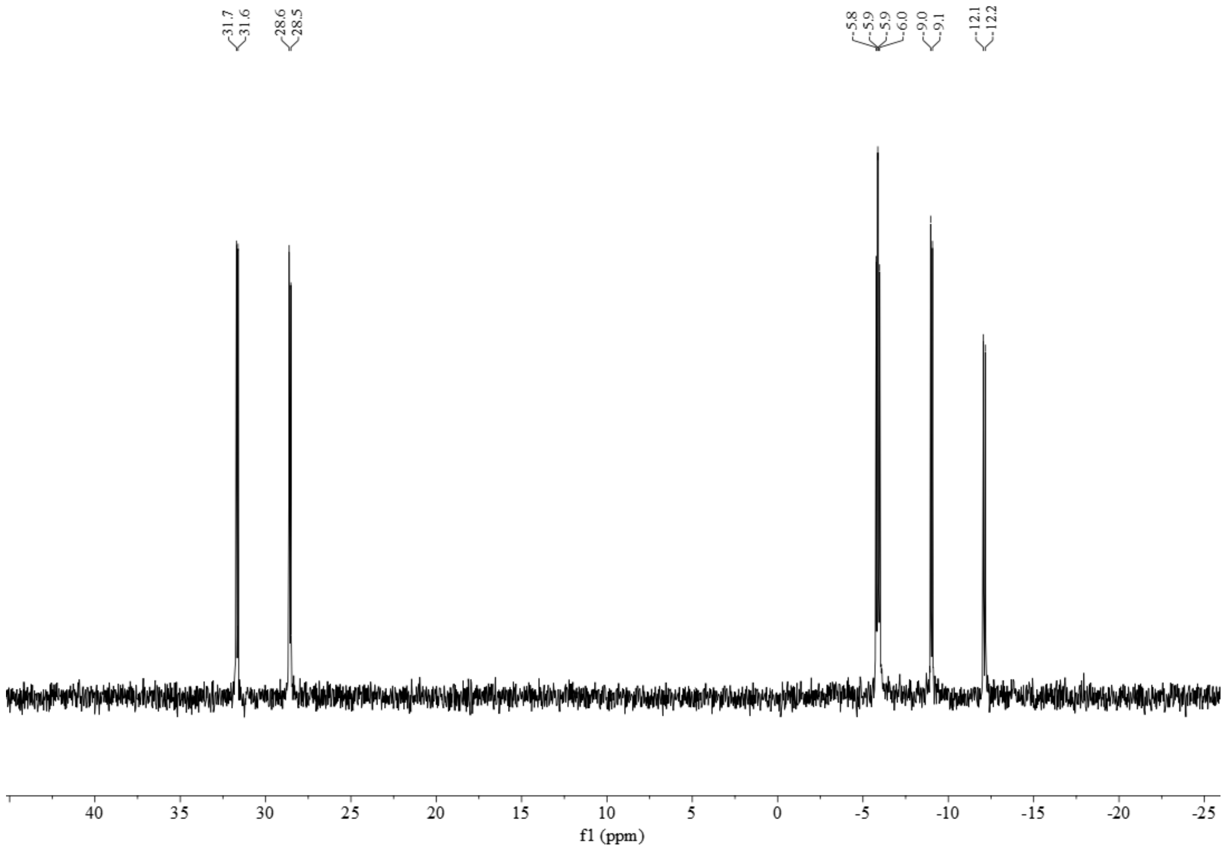

Fig. S10. $^{31}P\{^1H\}$ NMR spectrum of [Pd(dfurpf)(PPh_2Fc)Cl][$BArF_{24}$] in CD_2Cl_2.

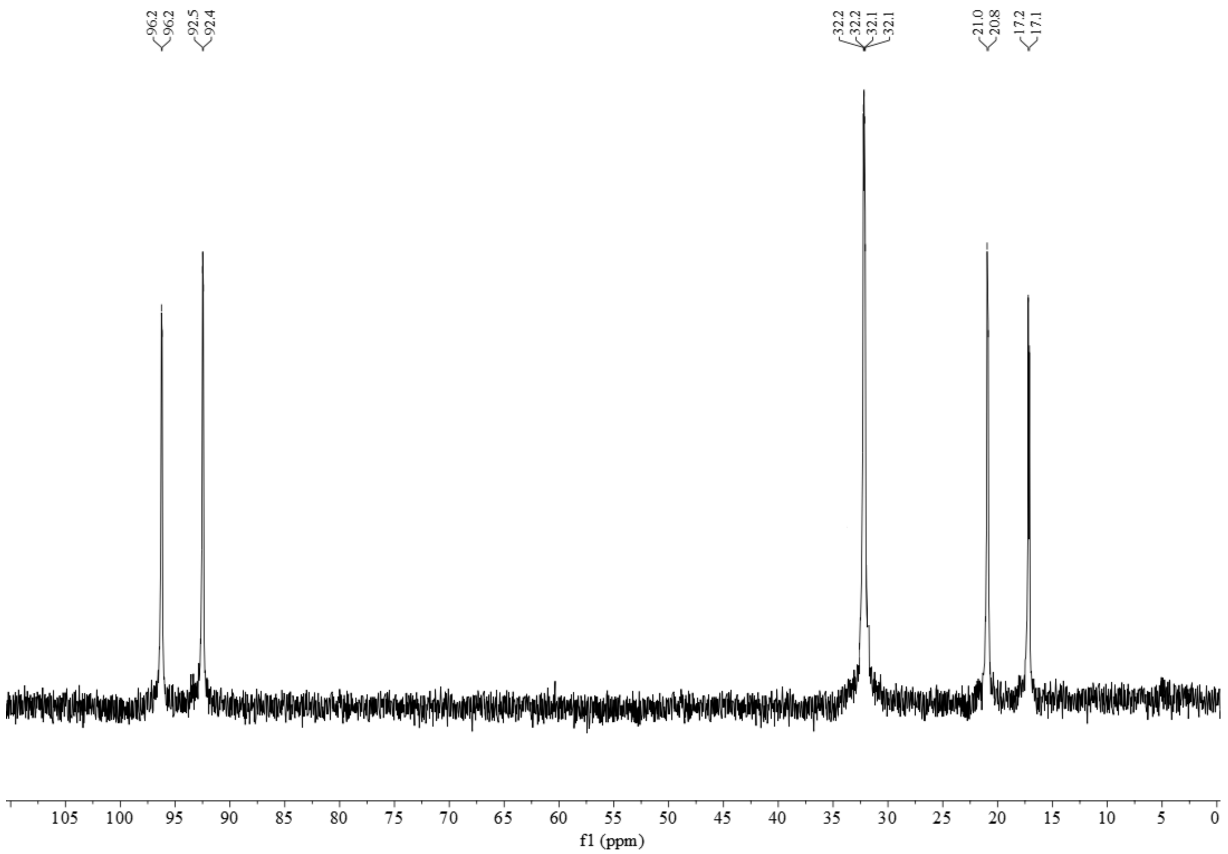

Fig. S11. $^{31}P\{^{1}H\}$ NMR spectrum of [Pd(dppf)(P(NMe$_2$)$_3$)Cl][BArF$_{24}$] in CD_2Cl_2.

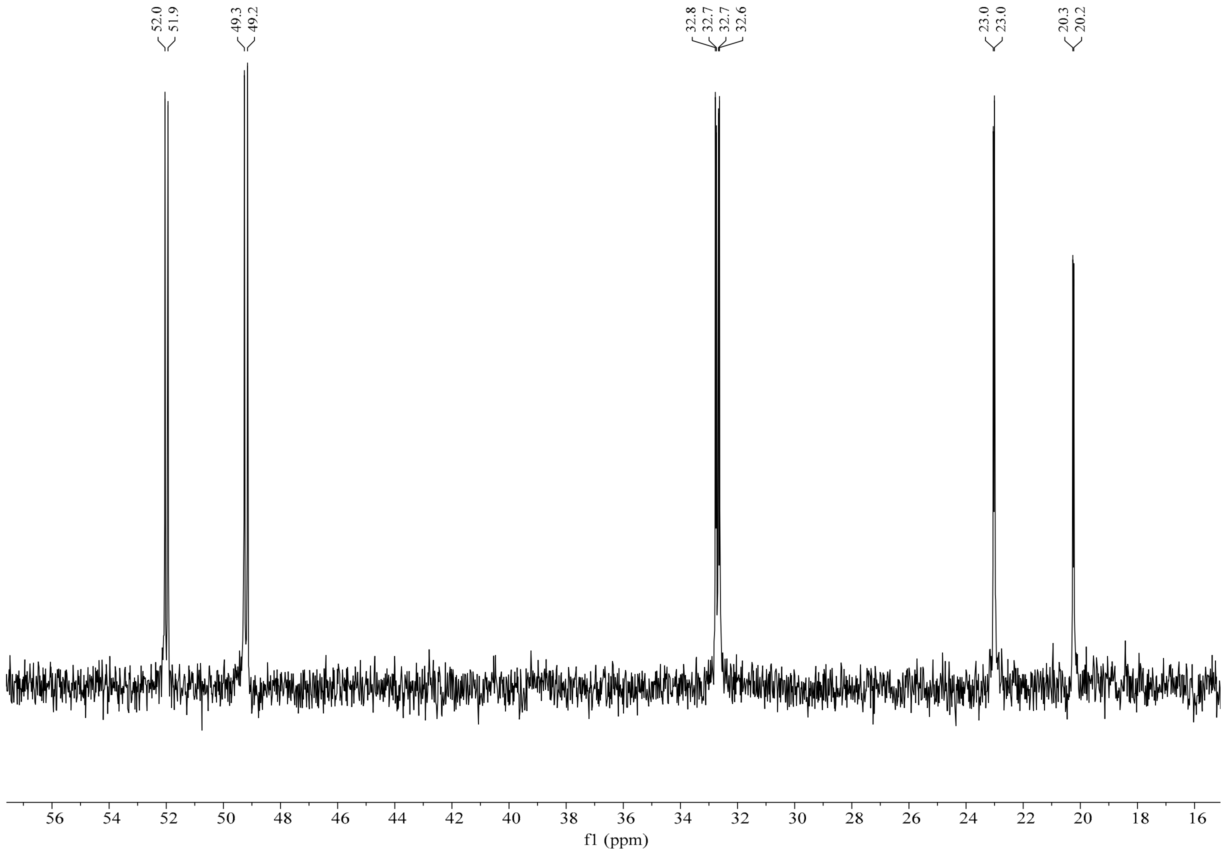

Fig. S12. $^{31}P\{^{1}H\}$ NMR spectrum of [Pd(dppf)(P^{i}Pr$_3$)Cl][BArF$_{24}$] in CD_2Cl_2.

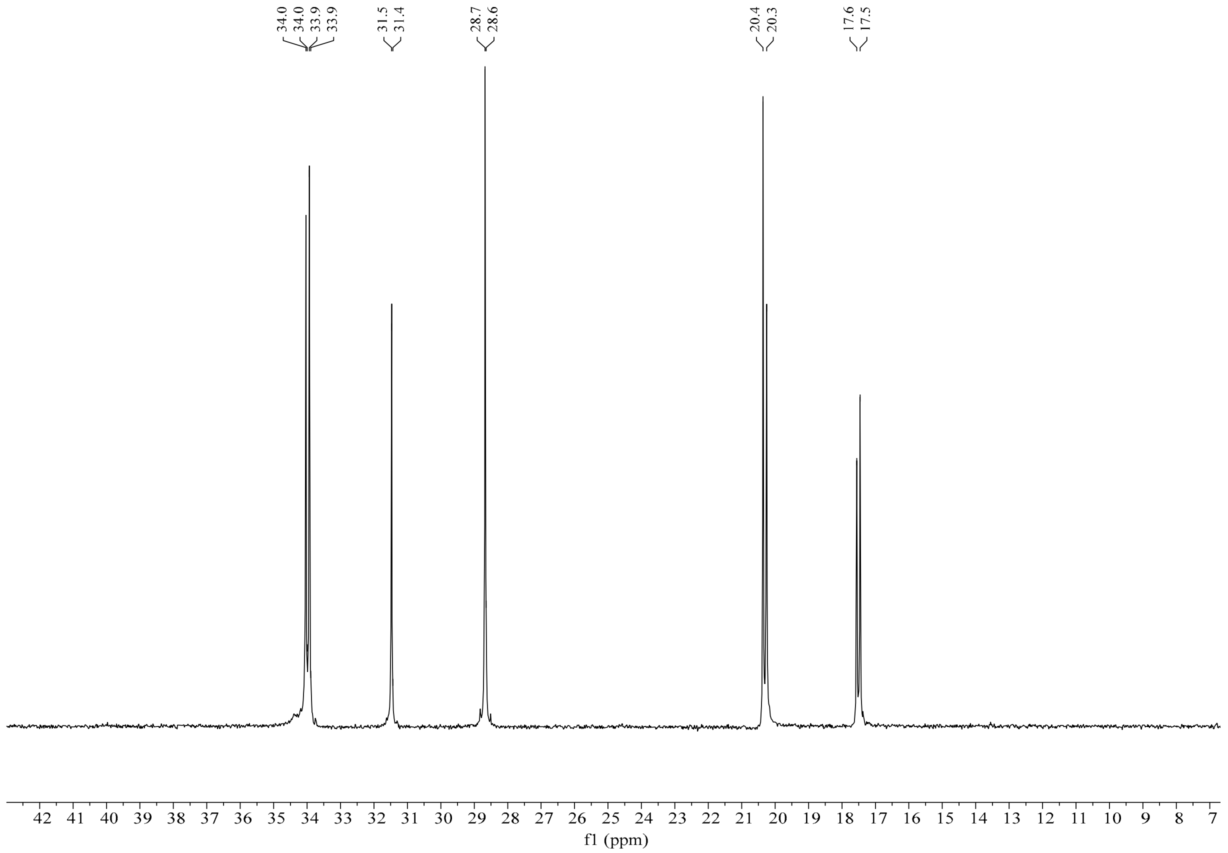

Fig. S13. $^{31}P\{^1H\}$ NMR spectrum of [Pd(dppf)(P(CH_2Ph)$_3$)Cl][BArF$_{24}$] in CD_2Cl_2.

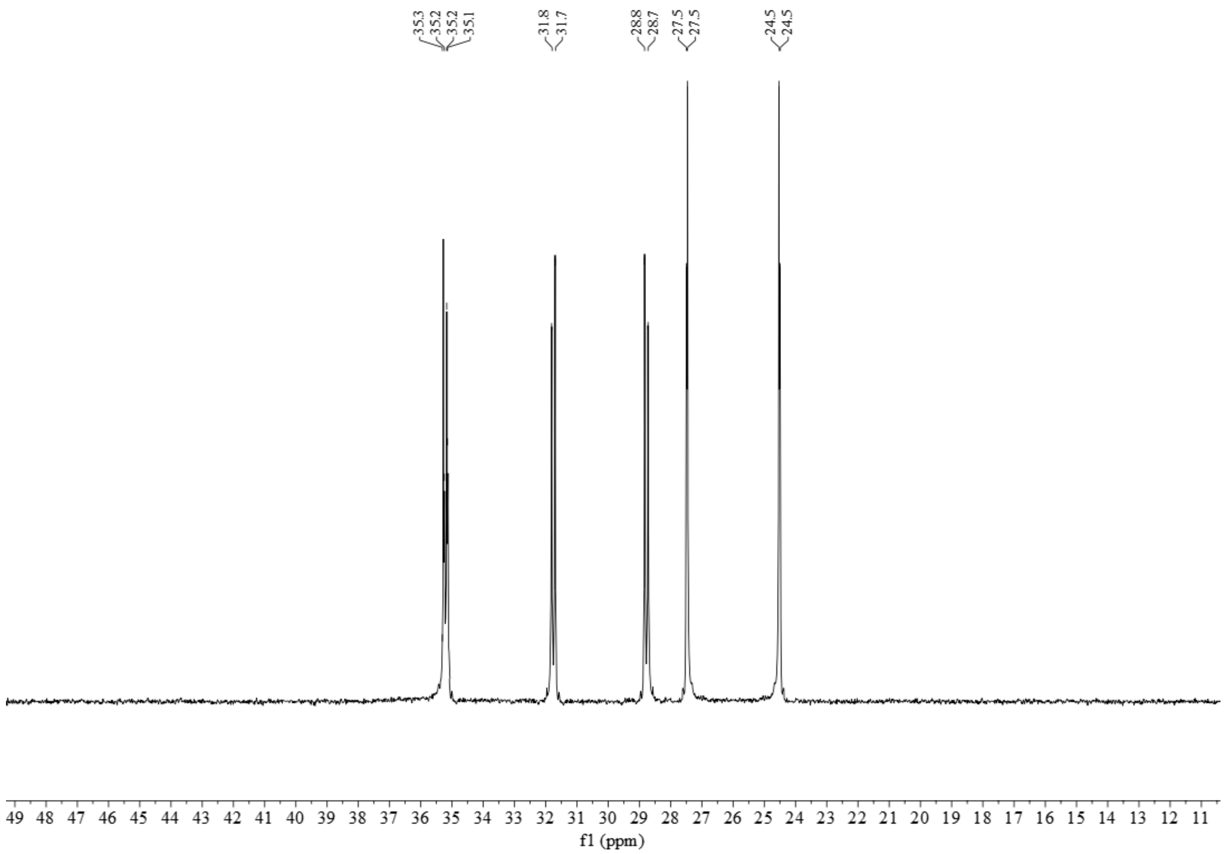

Fig. S14. $^{31}P\{^{1}H\}$ NMR spectrum of [Pd(dppf)(P(*m*-tol)$_3$)Cl][BArF$_{24}$] in CD_2Cl_2.

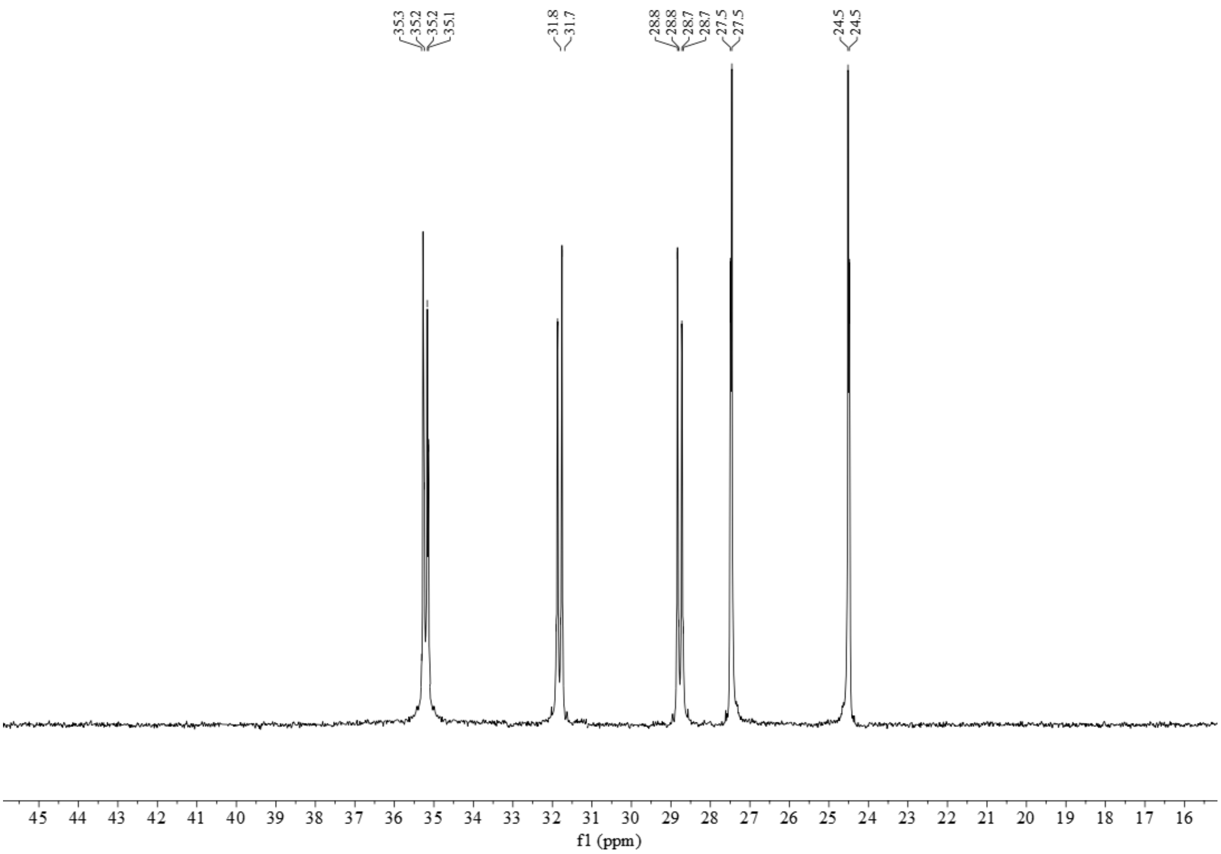

Fig. S15. $^{31}P\{^1H\}$ NMR spectrum of [Pd(dppf)(P(*p*-tol)$_3$)Cl][BArF$_{24}$] in CD_2Cl_2.

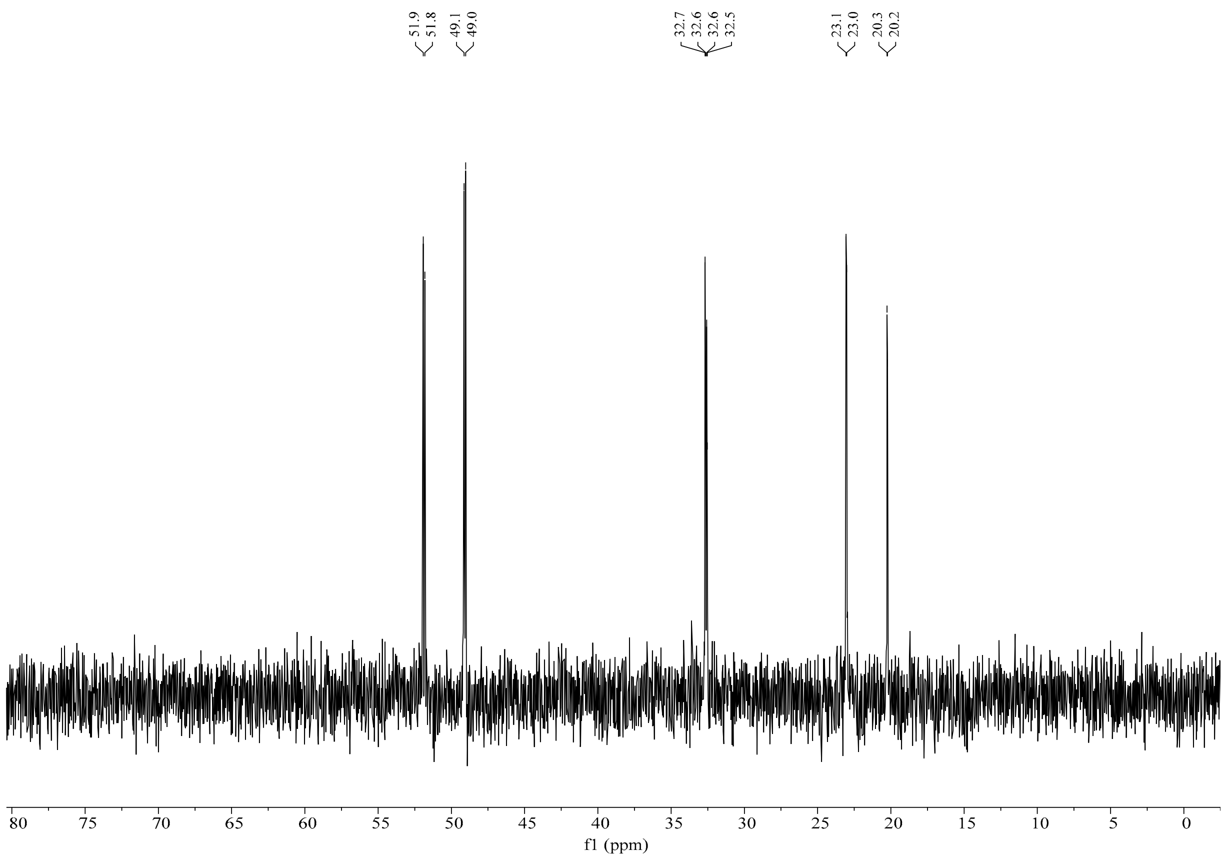

Fig. S16. $^{31}P\{^{1}H\}$ NMR spectrum of [Pd(dppf)(P(*p*-$C_6H_4OMe)_3$)Cl][BArF$_{24}$] in CD_2Cl_2.

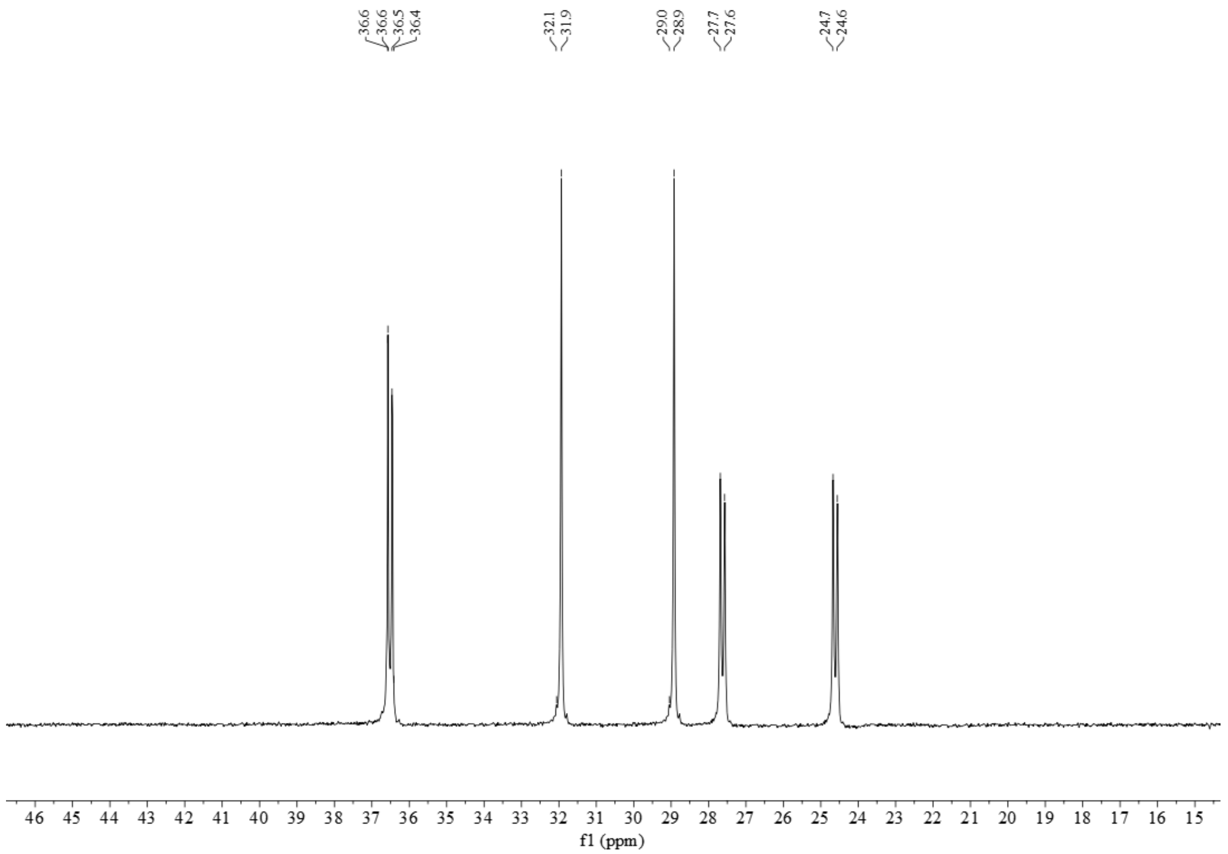

Fig. S17. $^{31}P\{^{1}H\}$ NMR spectrum of [Pd(dppf)(P(*p*-$C_6H_4F)_3$)Cl][BArF$_{24}$] in CD_2Cl_2.

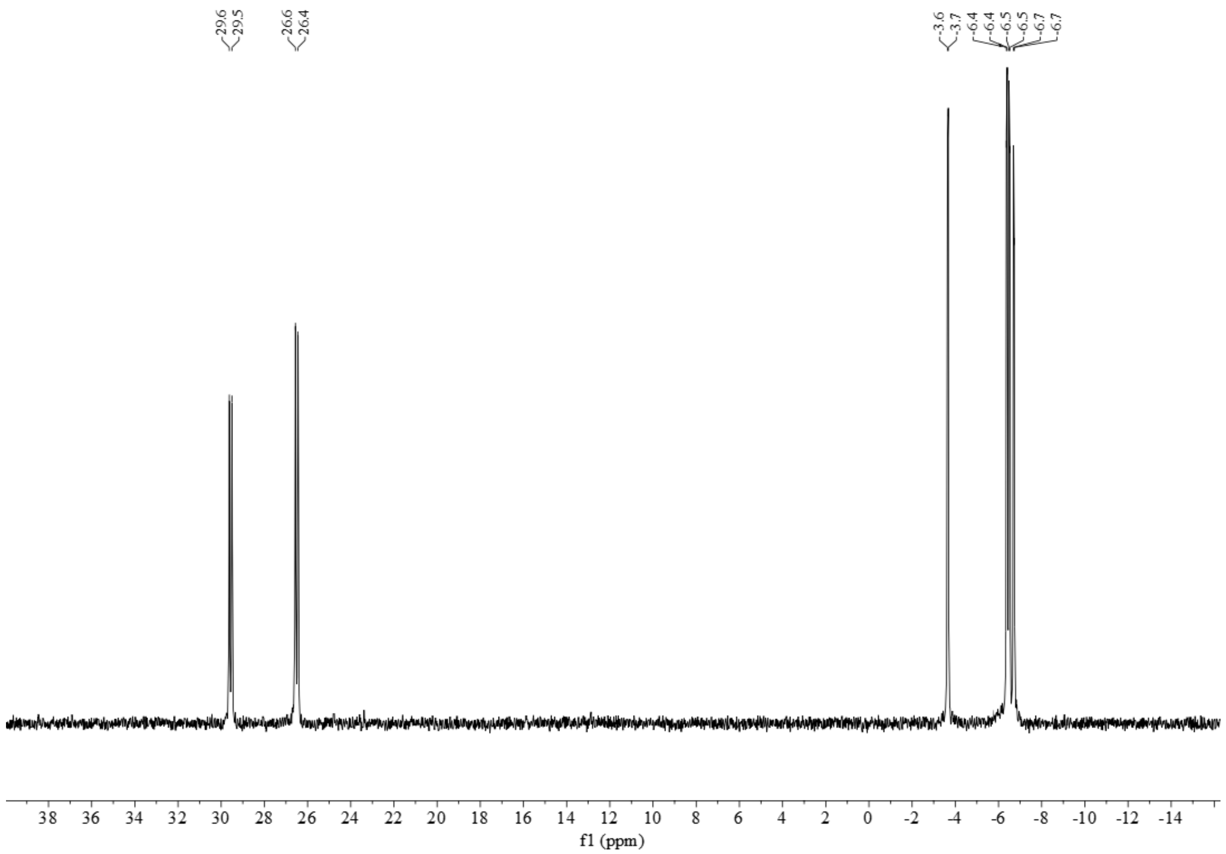

Fig. S18. $^{31}P\{^1H\}$ NMR spectrum of $[Pd(dfurpf)(P(p\text{-}C_6H_4CF_3)_3)Cl][BArF_{24}]$ in CD_2Cl_2.

Table S1.

Crystal data and structure analysis results.

	$[Pd_2(dppdtbpf)_2(\mu\text{-}Cl)_2][BArF_{24}]_2$
formula	$C_{124}H_{96}B_2Cl_2F_{48}Fe_2P_4Pd_2$
fw	3293.68
crystal system	triclinic
space group	$P\bar{1}$
a, Å	13.3640(4)
b, Å	14.1731(4)
c, Å	18.8640(5)
α, deg	107.137(2)
β, deg	99.478(2)
γ, deg	95.132(2)
V, $Å^3$	3331.50(17)
Z	1
cryst. size, mm	0.37 x 0.25 x 0.14
cryst. color	Green-brown
radiation	0.71073
temp, K	101.15
2θ range, deg	4.356-61.194
data collected	
h	-19 to 19
k	-19 to 20
l	-26 to 26
no. of data collected	90876
no. of unique data	19504
abs. corr	SCALE3 ABSPACK
final *R* indices	
R1	0.0447
wR2	0.0977
goodness of fit	1.026

Table S2.

Crystal data and structure analysis results for monodentate alkyl phoshines.

	**[Pd(dippf)(PMe$_3$)Cl][BArF$_{24}$]**	**[Pd(dcpf)(PMe$_3$)Cl][BArF$_{24}$]**	**[Pd(dppf)(P^{i}Pr$_3$)Cl][BArF$_{24}$]**
formula	$C_{114}H_{114}B_2Cl_2F_{48}Fe_2P_6Pd_2$	$C_{69}H_{73}BClF_{24}FeP_3Pd$	$C_{76}H_{63}BCl_3F_{24}FeP_3Pd$
fw	2998.89	1786.85	1889.51
crystal system	monoclinic	triclinic	triclinic
space group	$P2_1/n$	$P\bar{1}$	$P\bar{1}$
a, Å	18.8630(5)	14.5144(5)	13.7811(2)
b, Å	17.1402(5)	14.9919(4)	16.8393(3)
c, Å	19.2625(7)	18.4885(6)	19.0527(3)
α, deg	90	105.066(3)	110.586(2)
β, deg	94.472(3)	105.113(3)	107.341(2)
γ, deg	90	94.469(3)	91.4480(10)
V, Å^3	6208.9(3)	3704.3(2)	3908.90(13))
Z	2	2	2
cryst. size, mm	0.44 x 0.40 x 0.16	0.21 x 0.15 x 0.09	0.50 x 0.24 x 0.12
cryst. color	Orange	Orange	Orange
radiation	0.71073	0.71073	0.71073
temp, K	100.15	99.97(11)	99.9(7)
2θ range, deg	4.242-51.998	4.214-61.226	4.548-61.292
data collected			
h	-23 to 23	-20 to 20	-19 to 19
k	-21 to 20	-21 to 21	-24 to 23
l	-23 to 23	-26 to 26	-27 to 27
no. of data collected	40031	104524	115081
no. of unique data	11952	21606	23000
abs. corr	SCALE3 ABSPACK	SCALE3 ABSPACK	SCALE3 ABSPACK
final *R* indices			
R1	0.0342	0.0686	0.0363
wR2	0.0815	0.1506	0.0867
goodness of fit	1.044	0.984	1.008

Table S3.
Crystal data and structure analysis results for monodentate aryl phosphines.

	**[Pd(dppf)(P(*p*-$C_6H_4F)_3$)Cl][BArF$_{24}$]**	**[Pd(dfurpf)(P(*p*-$C_6H_4CF_3)_3$)Cl][BArF$_{24}$]**
formula	$C_{168}H_{104}B_2Cl_2F_{54}Fe_2P_6Pd_2$	C_{168}H108$B_2Cl_6F_{66}Fe_2O_8P_6Pd_2$
fw	3293.68	4252.80
crystal system	monoclinic	triclinic
space group	$P2_1/c$	$P\bar{1}$
a, Å	22.2520(11)	10.6072(4)
b, Å	20.9008(8)	19.0430(7)
c, Å	18.6833(8)	21.9658(8)
α, deg	107.137(2)	86.413(3)
β, deg	90	80.154(3)
γ, deg	111.786(5)	83.342(3)
V, Å^3	90	4338.0(3)
Z	2	1
cryst. size, mm	0.29 x 0.22 x 0.06	0.16 x 0.13 x 0.11
cryst. color	Orange	orange
radiation	0.71073	0.71073
temp, K	100(2)	100.15
2θ range, deg	4.24-50	3.92-52
data collected		
h	-26 to 26	-13 to 13
k	-24 to 24	-23 to 23
l	-22 to 22	-27 to 27
no. of data collected	88989	96097
no. of unique data	14216	16896
abs. corr	SCALE3 ABSPACK	SCALE3 ABSPACK
final *R* indices		
R1	0.0567	0.0674
wR2	0.1350	0.1284
goodness of fit	1.012	1.005

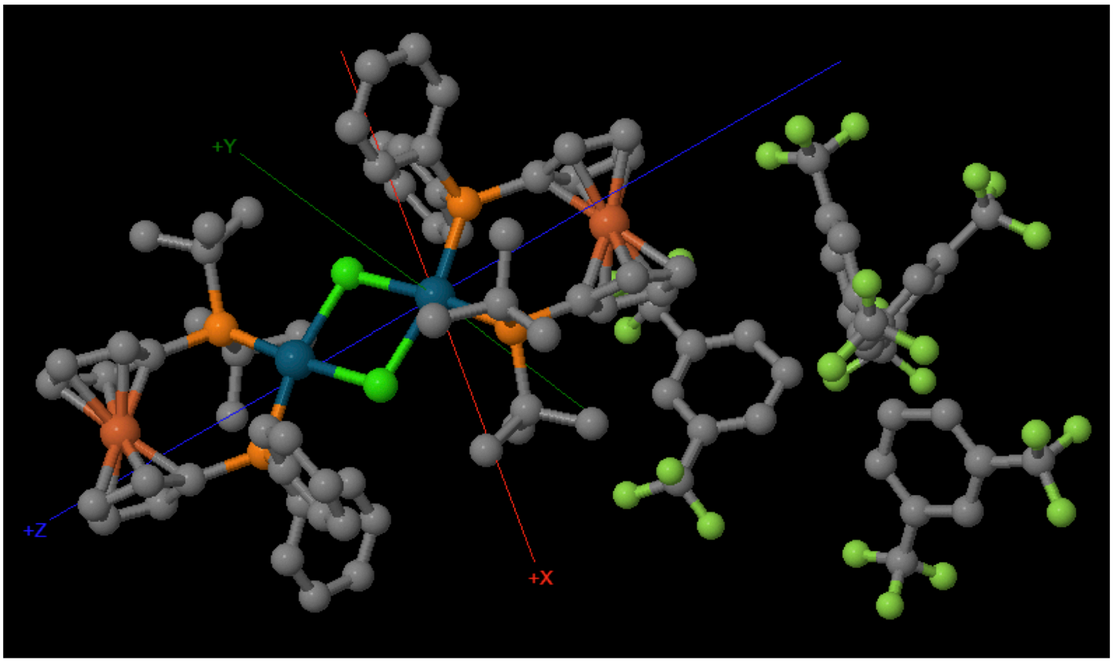

%V Free	%V Buried	%V tot/V Ex
44.0	**60.0**	99.9

Quadrant	V f	V b	V t	%V f	%V b
SW	18.9	25.9	44.9	42.2	57.8
NW	17.8	27.1	44.9	39.7	60.3
NE	17.0	27.9	44.9	37.9	62.1
SE	18.0	26.9	44.8	40.1	59.9

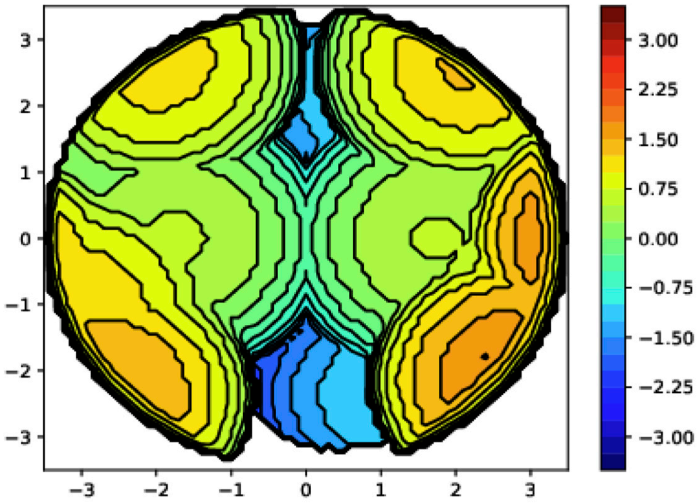

Fig. S19. %V_{bur} calculation for the dppdtbpf ligand in $[Pd_2(dppdtbpf)_2(\mu\text{-}Cl)_2][BArF_{24}]_2$.

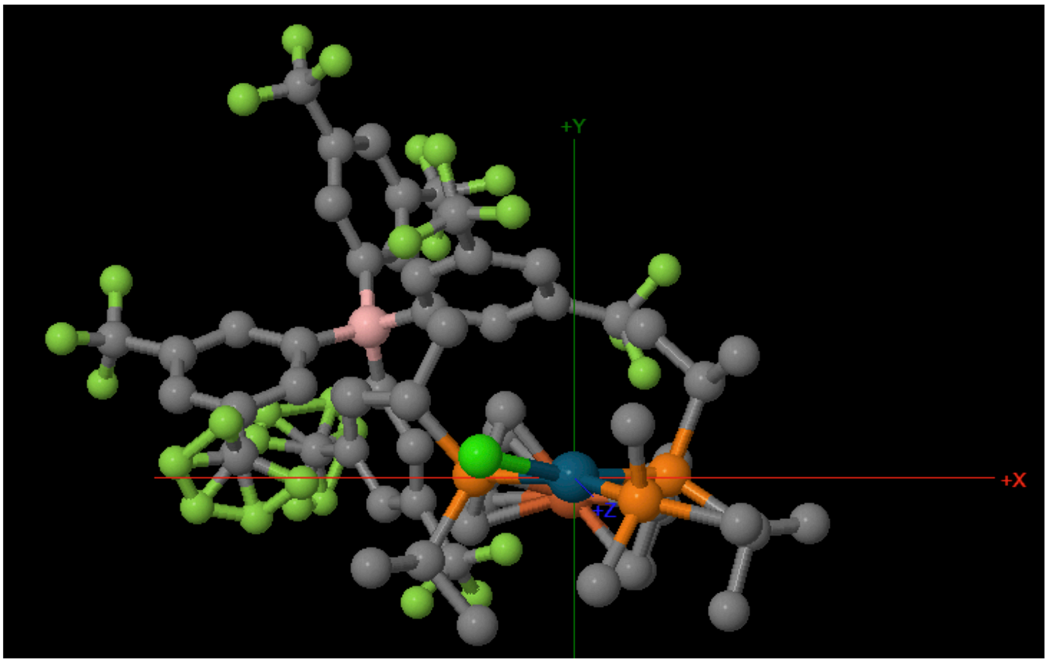

%V Free	**%V Buried**	%V tot/V Ex
43.4	**56.6**	99.9

**Quadrant**	**V f**	**V b**	**V t**	**%V f**	**%V b**
SW	18.3	26.6	44.9	40.7	59.3
NW	21.0	23.9	44.9	46.8	53.2
NE	18.0	26.9	44.9	40.0	60.0
SE	20.7	24.1	44.9	46.2	53.8

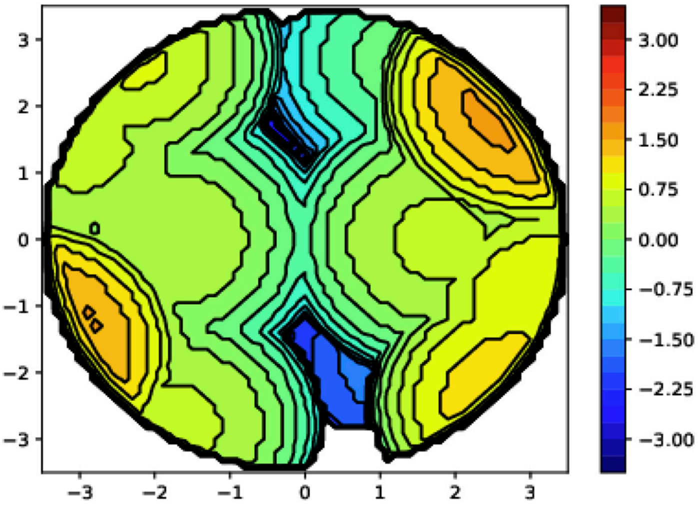

Fig. S20. %V_{bur} calculation for the dippf ligand in [Pd(dippf)(PMe_3)Cl][BArF$_{24}$].

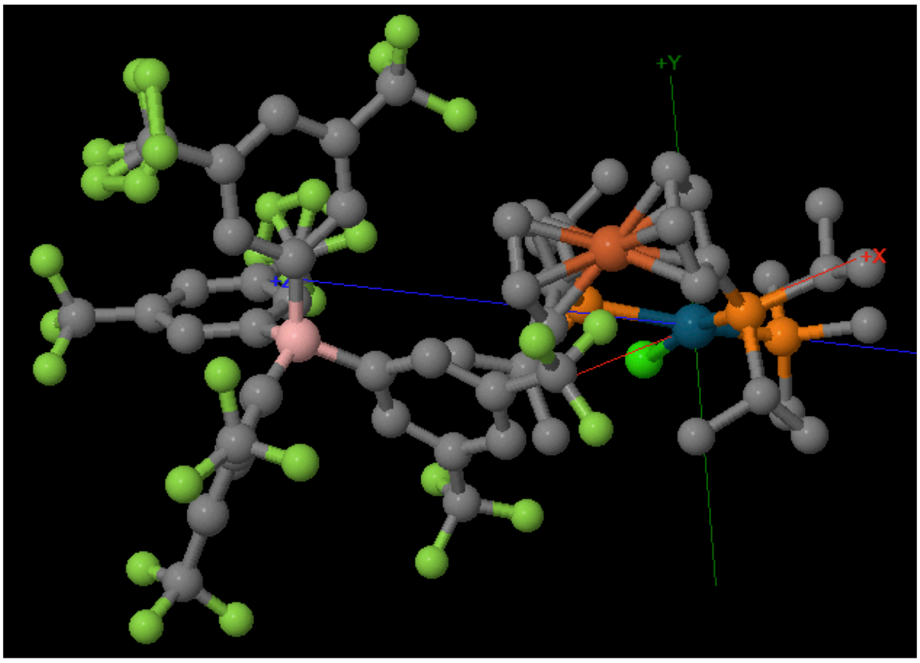

%V Free	**%V Buried**	%V tot/V Ex
78.4	**21.6**	99.9

**Quadrant**	**V f**	**V b**	**V t**	**%V f**	**%V b**
SW	34.5	10.3	44.9	77.0	23.0
NW	33.7	11.1	44.9	75.2	24.8
NE	36.0	8.8	44.9	80.3	19.7
SE	36.4	8.4	44.9	81.2	18.8

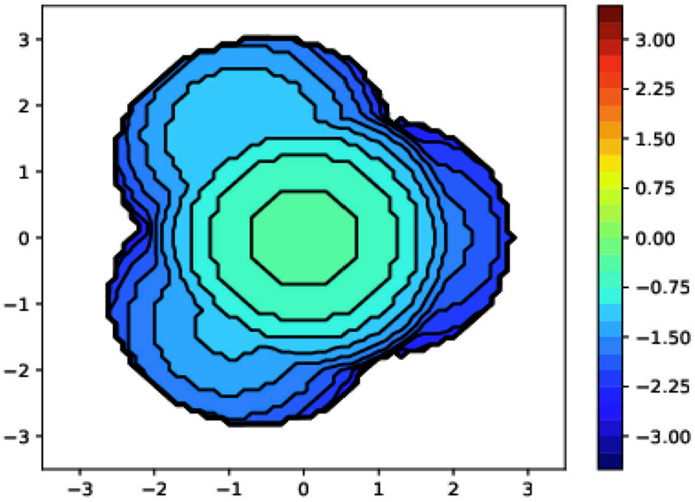

Fig. S21. %V_{bur} calculation for the PMe_3 ligand in [Pd(dippf)(PMe_3)Cl][$BArF_{24}$].

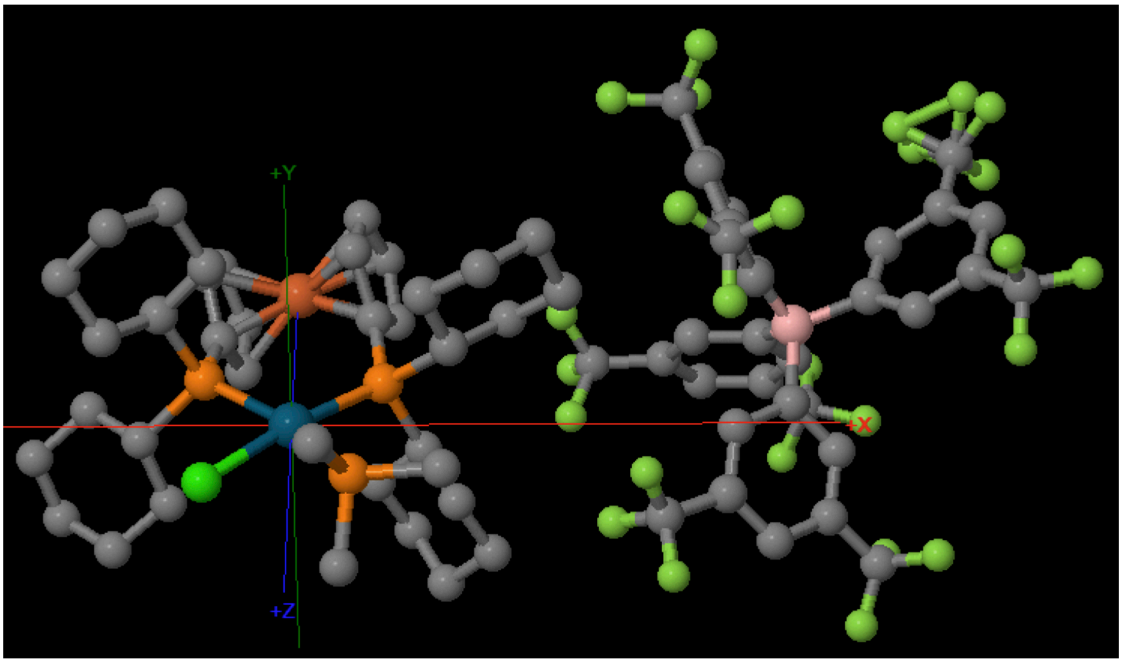

%V Free	%V Buried	%V tot/V Ex
45.0	**55.0**	99.9

Quadrant	V f	V b	V t	%V f	%V b
SW	22.2	22.7	44.9	49.5	50.5
NW	19.5	25.4	44.9	43.5	56.5
NE	20.4	24.4	44.9	45.5	54.5
SE	18.6	26.2	44.9	41.5	58.5

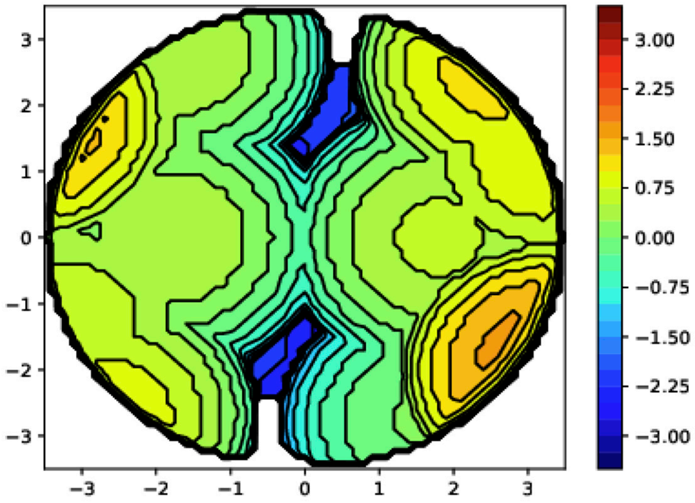

Fig. S22. $\%V_{bur}$ calculation for the dcpf ligand in $[Pd(dcpf)(PMe_3)Cl][BArF_{24}]$.

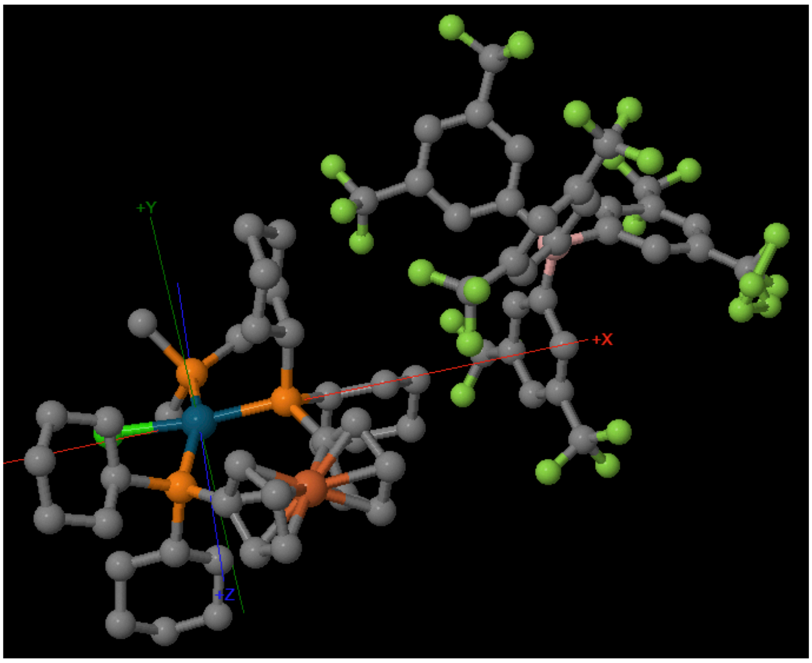

%V Free	**%V Buried**	%V tot/V Ex
78.5	**21.5**	99.9

**Quadrant**	**V f**	**V b**	**V t**	**%V f**	**%V b**
SW	33.8	11.0	44.9	75.5	24.5
NW	34.3	10.6	44.9	76.4	23.6
NE	36.5	8.4	44.9	81.3	18.7
SE	36.2	8.6	44.9	80.8	19.2

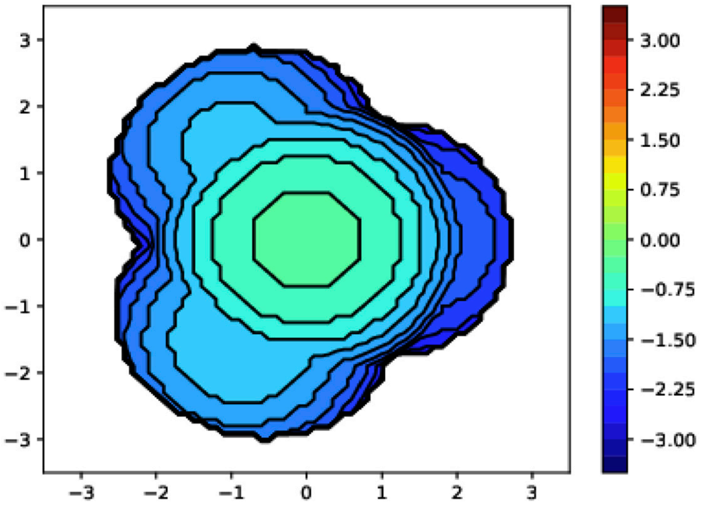

Fig. S23. %V_{bur} calculation for the PMe_3 ligand in [Pd(dcpf)(PMe_3)Cl][$BArF_{24}$].

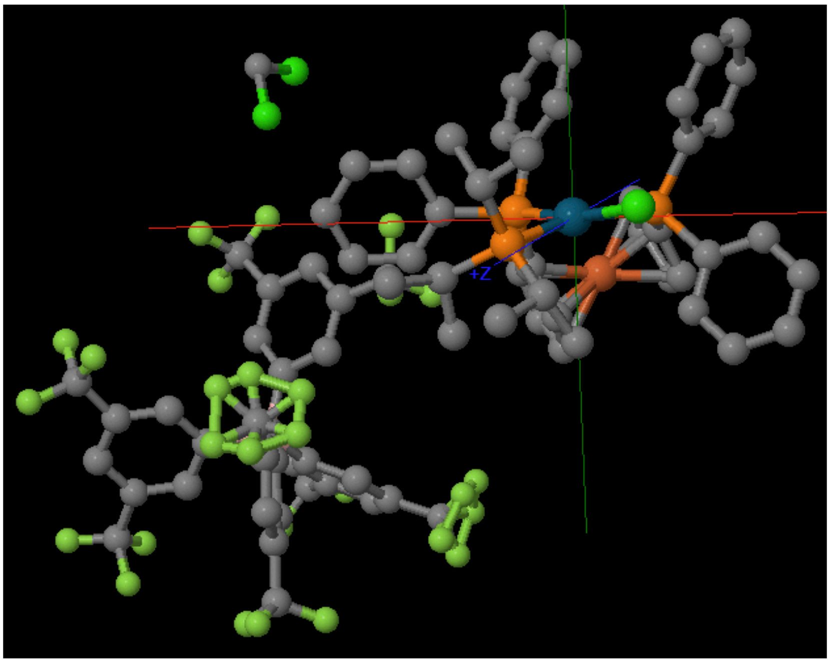

%V Free	**%V Buried**	%V tot/V Ex
47.9	**52.1**	99.9

**Quadrant**	**V f**	**V b**	**V t**	**%V f**	**%V b**
SW	22.8	22.0	44.9	50.9	49.1
NW	21.8	23.1	44.9	48.5	51.5
NE	21.6	23.3	44.9	48.1	51.9
SE	19.8	25.1	44.9	44.1	55.9

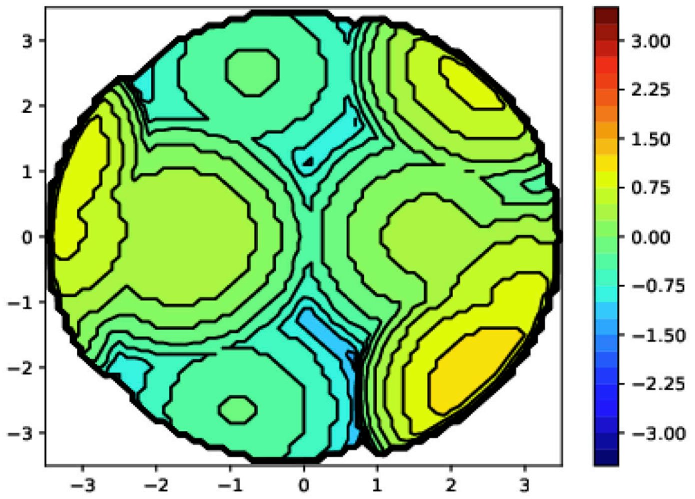

Fig. S24. $\%V_{bur}$ calculation for the dppf ligand in [Pd(dppf)(P^iPr_3)Cl][$BArF_{24}$].

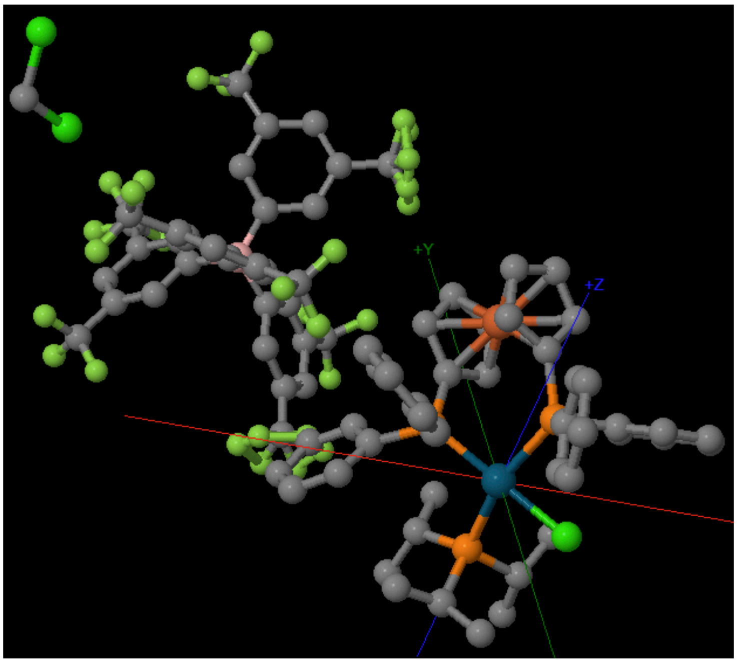

%V Free	**%V Buried**	%V tot/V Ex
70.9	**29.1**	99.9

**Quadrant**	**V f**	**V b**	**V t**	**%V f**	**%V b**
SW	31.3	13.5	44.9	69.9	30.1
NW	33.5	11.3	44.9	74.8	25.2
NE	29.4	15.5	44.9	65.5	34.5
SE	33.0	11.9	44.9	73.5	26.5

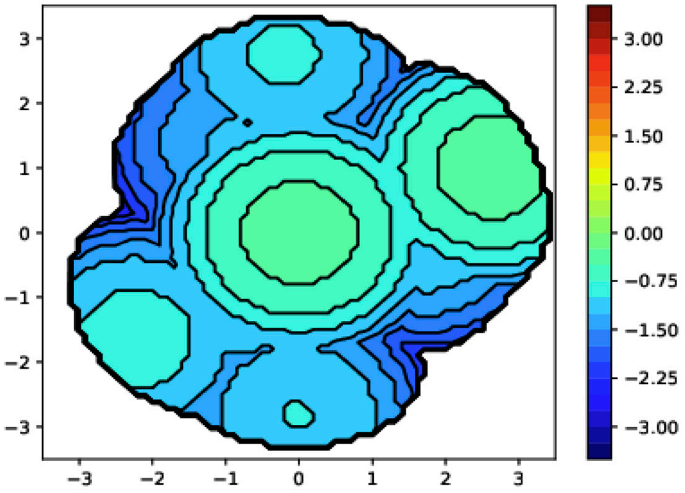

Fig. S25. $\%V_{bur}$ calculation for the $P^{i}Pr_3$ ligand in $[Pd(dppf)(P^{i}Pr_3)Cl][BArF_{24}]$.

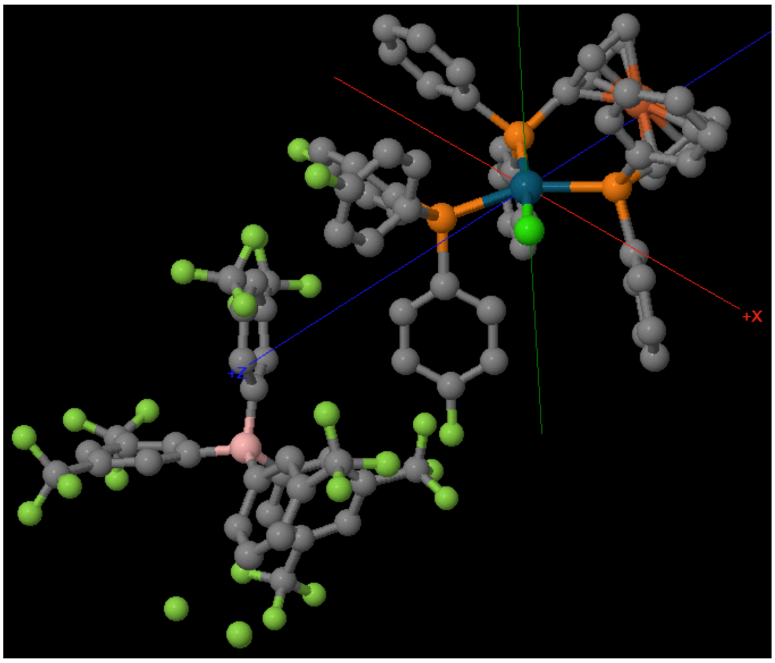

%V Free	**%V Buried**	%V tot/V Ex
43.9	**56.1**	99.9

**Quadrant**	**V f**	**V b**	**V t**	**%V f**	**%V b**
SW	19.8	25.1	44.9	44.1	55.9
NW	18.1	26.7	44.9	40.4	59.6
NE	20.0	24.9	44.9	44.5	55.5
SE	20.9	23.9	44.9	46.6	53.4

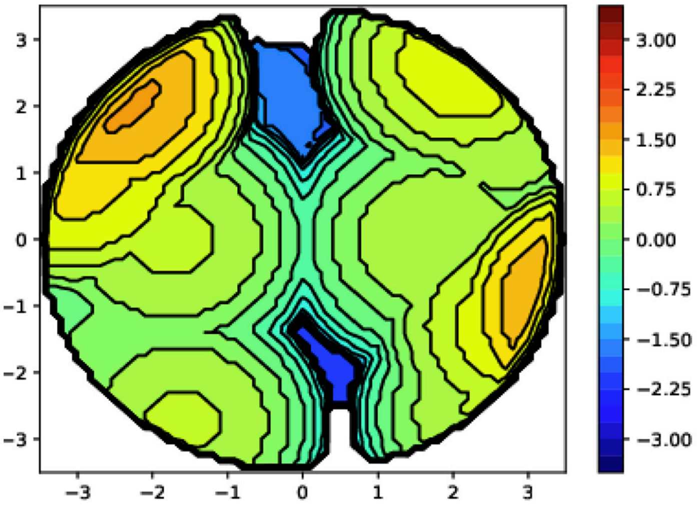

Fig. S26. %V_{bur} calculation for the dppf ligand in [Pd(dppf)(P(*p*-C_6H_4F)$_3$)Cl][BArF$_{24}$].

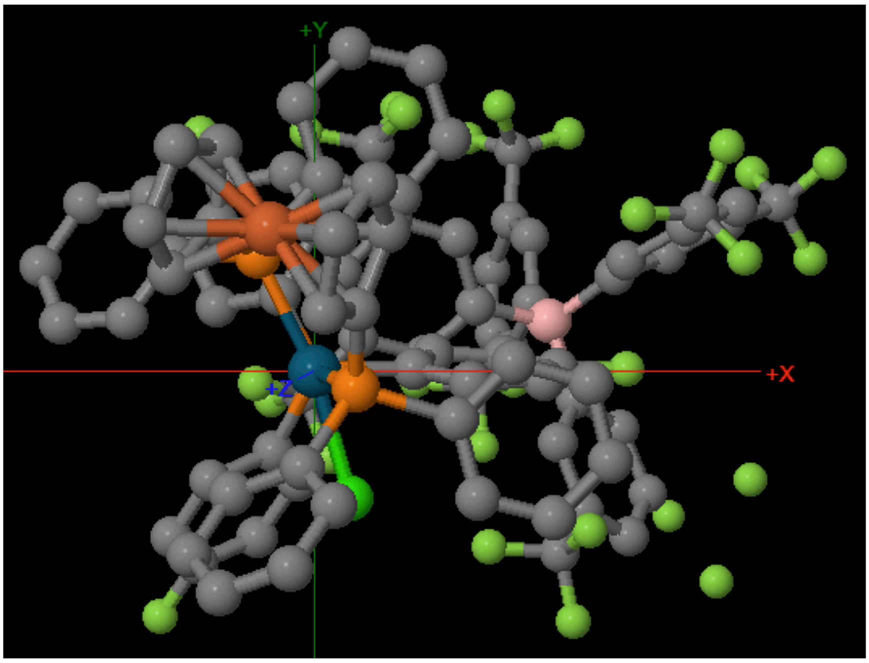

%V Free	**%V Buried**	%V tot/V Ex
72.1	**27.9**	99.9

**Quadrant**	**V f**	**V b**	**V t**	**%V f**	**%V b**
SW	30.4	14.5	44.9	67.8	32.2
NW	35.5	9.4	44.9	79.2	20.8
NE	32.1	12.8	44.9	71.5	28.5
SE	31.3	13.5	44.9	69.8	30.2

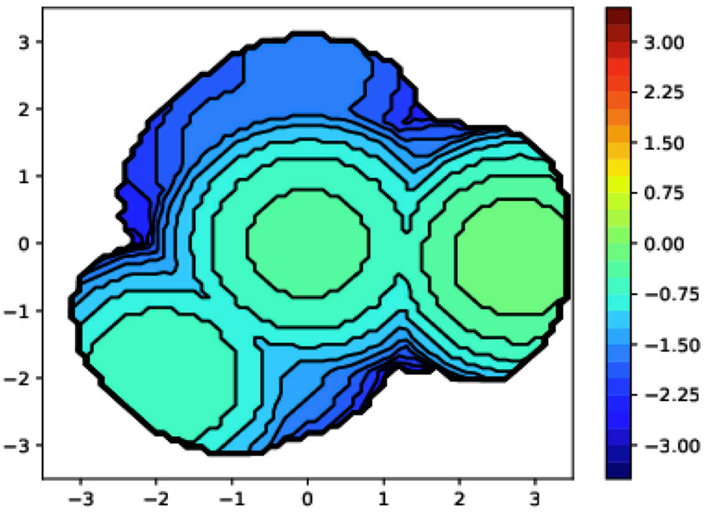

Fig. S27. %V_{bur} calculation for the P(*p*-C_6H_4F)$_3$ ligand in [Pd(dppf)(P(*p*-C_6H_4F)$_3$)Cl][BArF$_{24}$].

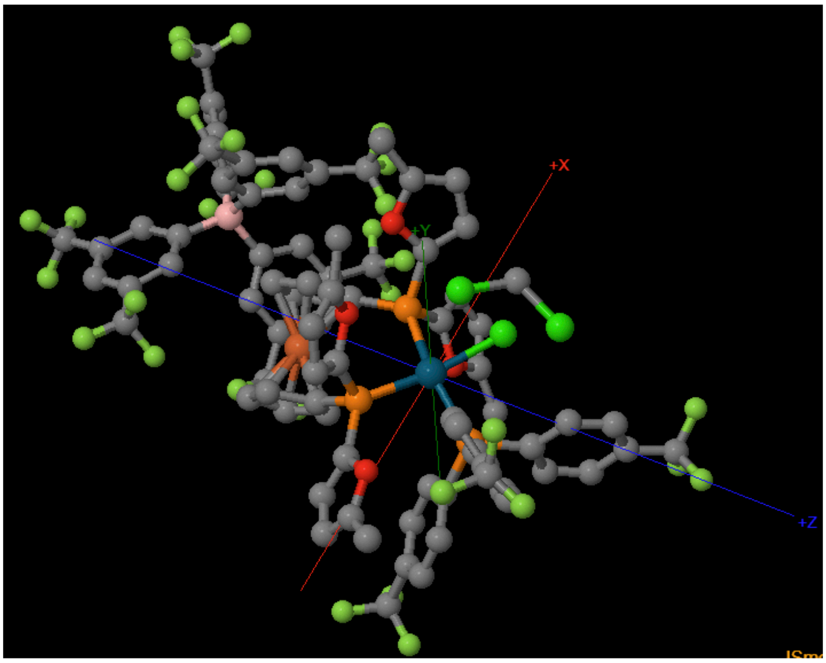

%V Free	%V Buried	%V tot/V Ex
48.7	**51.3**	99.9

Quadrant	V f	V b	V t	%V f	%V b
SW	24.2	20.7	44.9	53.9	46.1
NW	21.4	23.5	44.9	47.7	52.3
NE	24.4	20.5	44.9	54.4	45.6
SE	17.4	27.4	44.9	38.8	61.2

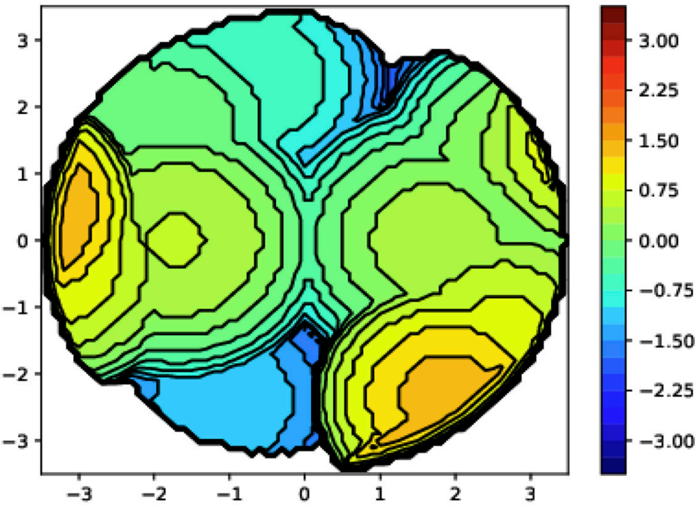

Fig. S28. $\%V_{bur}$ calculation for the dfurpf ligand in [Pd(dfurpf)(P(*p*-$C_6H_4CF_3$)$_3$)Cl][BArF$_{24}$].

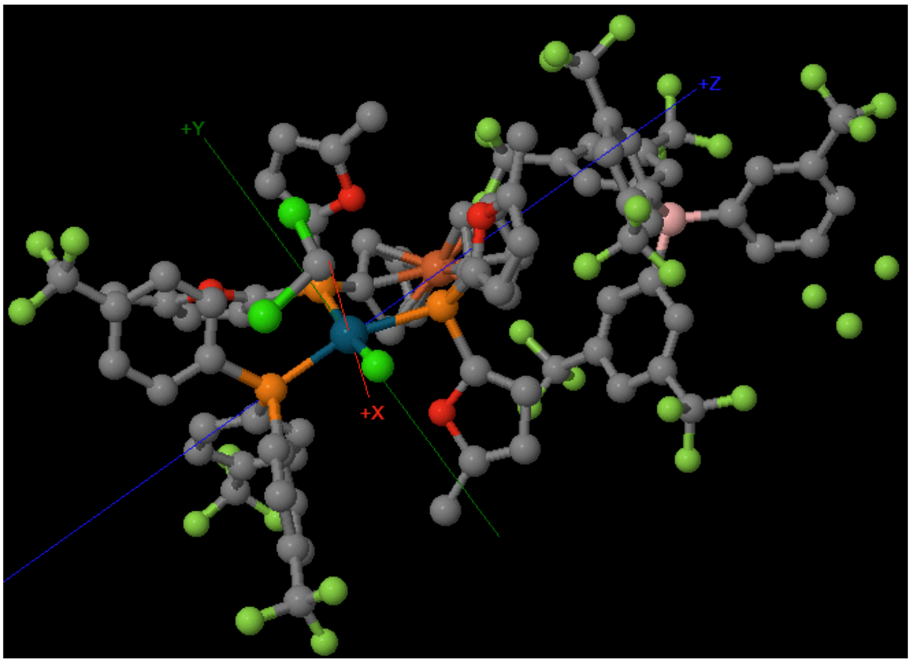

%V Free	**%V Buried**	%V tot/V Ex
71.3	**28.7**	99.9

Quadrant	V f	V b	V t	%V f	%V b
SW	33.6	11.3	44.9	74.9	25.1
NW	30.3	14.5	44.9	67.6	32.4
NE	34.2	10.6	44.9	76.3	23.7
SE	29.8	15.1	44.9	66.4	33.6

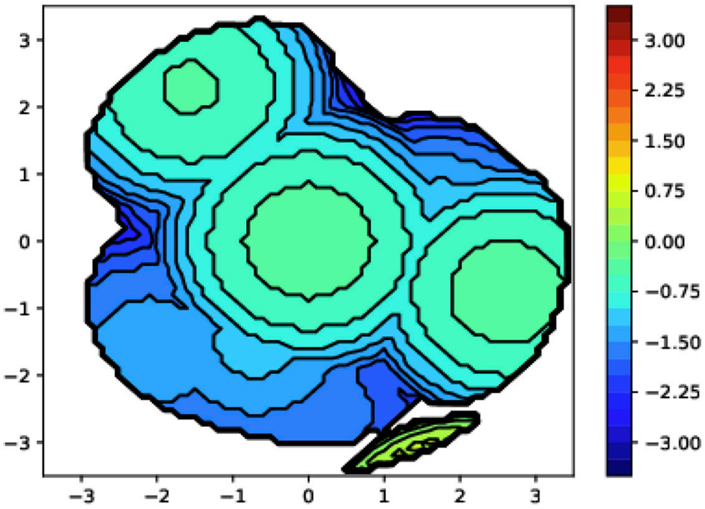

Fig. S29. $\%V_{bur}$ calculation for the $P(p\text{-}C_6H_4CF_3)_3$ ligand in $[Pd(dfurpf)(P(p\text{-}C_6H_4CF_3)_3)Cl][BArF_{24}]$.

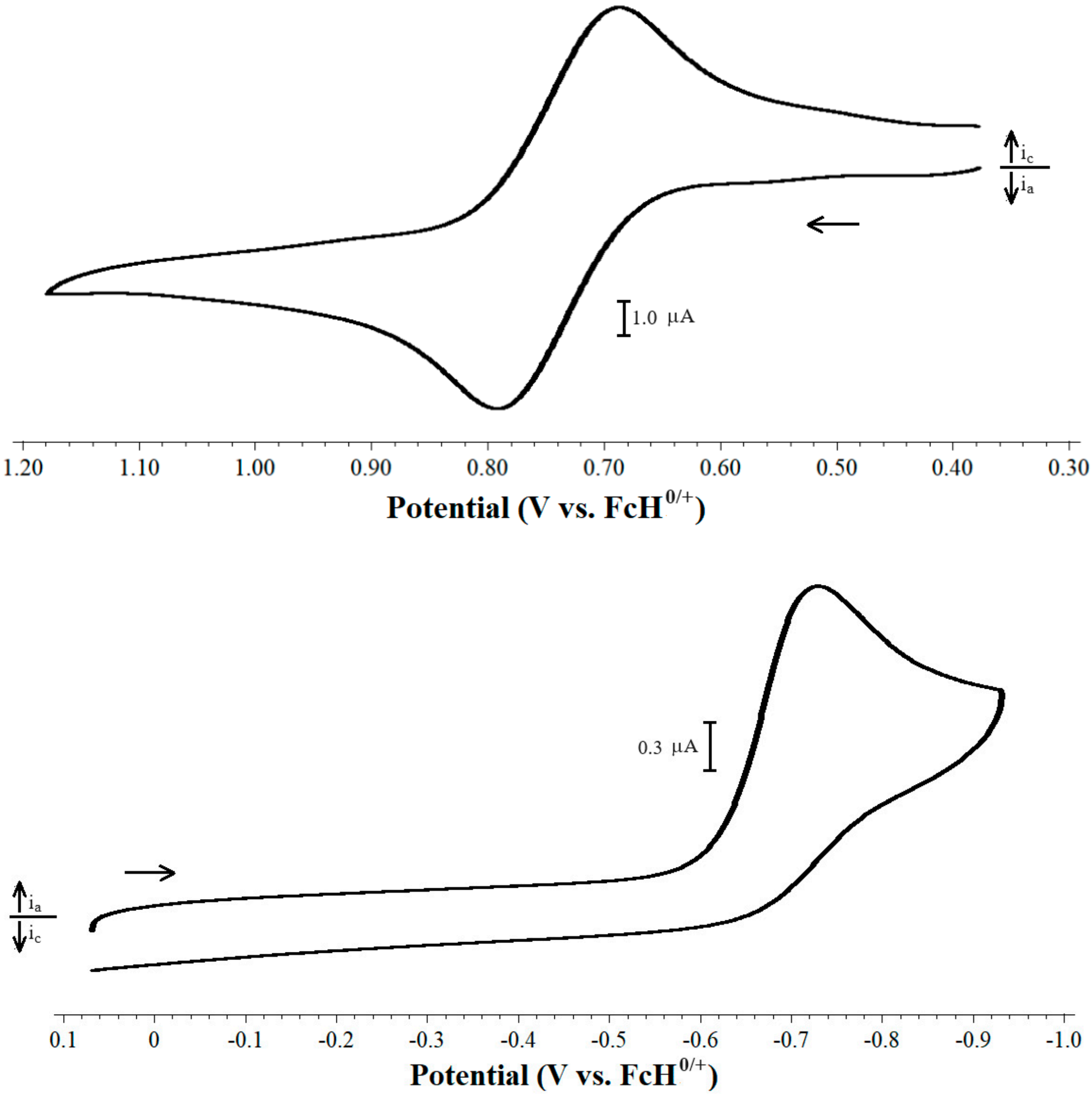

Fig. S30. CV scans of 1.0 mM $[Pd_2(dfurpf)_2(\mu\text{-}Cl)_2][BArF_{24}]_2$ with 0.1 M $[NBu_4][PF_6]$ as the supporting electrolyte measured at 100 mV s^{-1}.

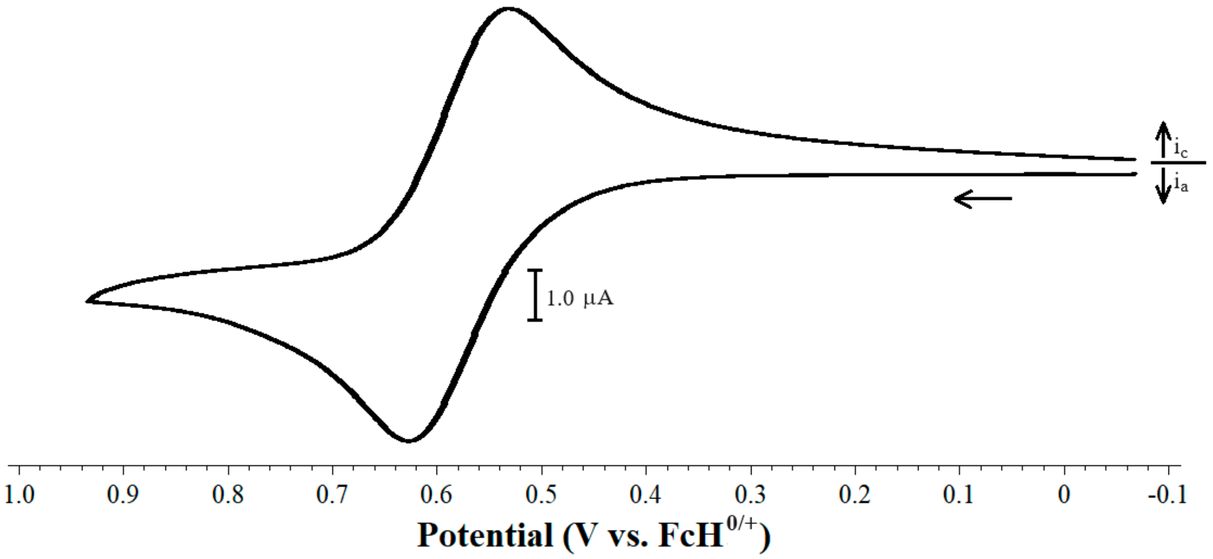

Fig. S31. CV scan of 1.0 mM [Pd(dippf)(PMe_3)Cl][$BArF_{24}$] with 0.1 M [NBu_4][PF_6] as the supporting electrolyte measured at 100 mV s^{-1}.

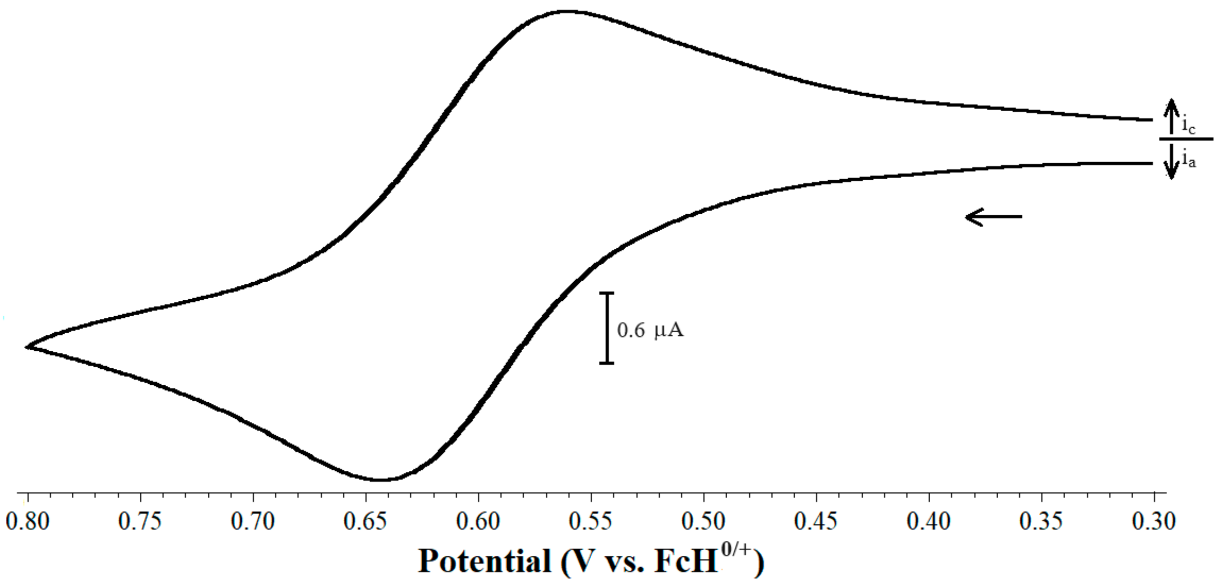

Fig. S32. CV scan of 1.0 mM [Pd(dippf)(PPh_3)Cl][$BArF_{24}$] with 0.1 M [NBu_4][PF_6] as the supporting electrolyte measured at 100 mV s^{-1}.

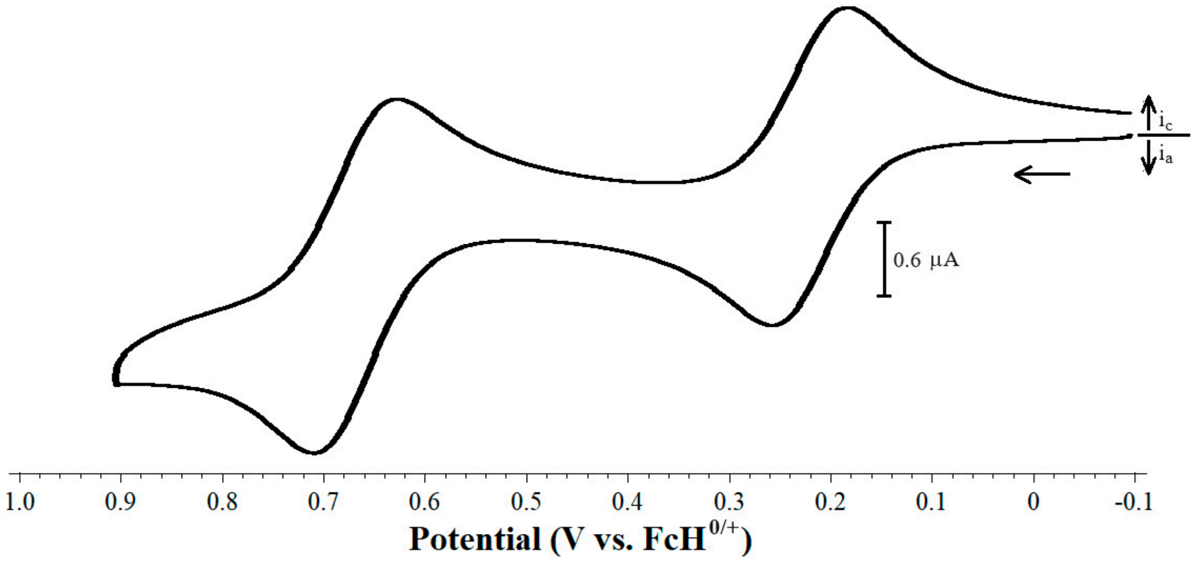

Fig. S33. CV scan of 1.0 mM [Pd(dippf)(PPh$_2$Fc)Cl][BArF$_{24}$] with 0.1 M [NBu$_4$][PF$_6$] as the supporting electrolyte measured at 100 mV s^{-1}.

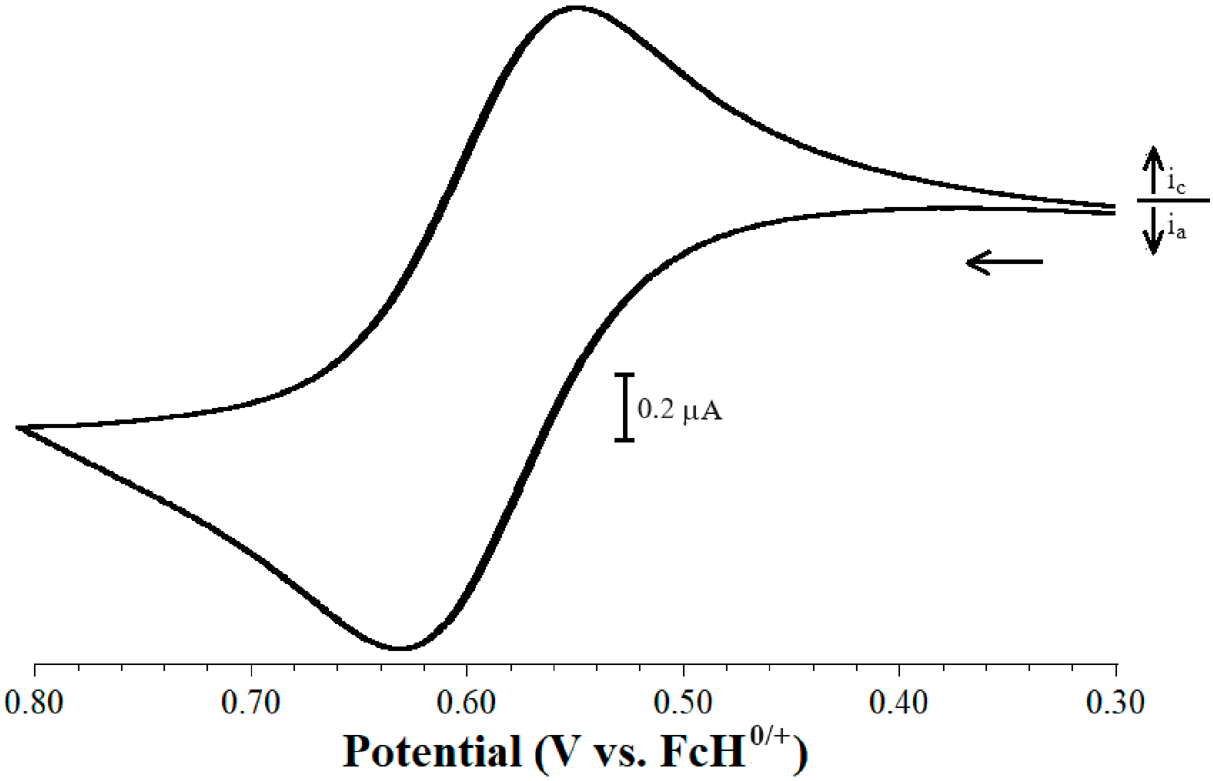

Fig. S34. CV scan of 1.0 mM [Pd(dppdtbpf)(PMe_3)Cl][$BArF_{24}$] with 0.1 M [NBu_4][PF_6] as the supporting electrolyte measured at 100 mV s^{-1}.

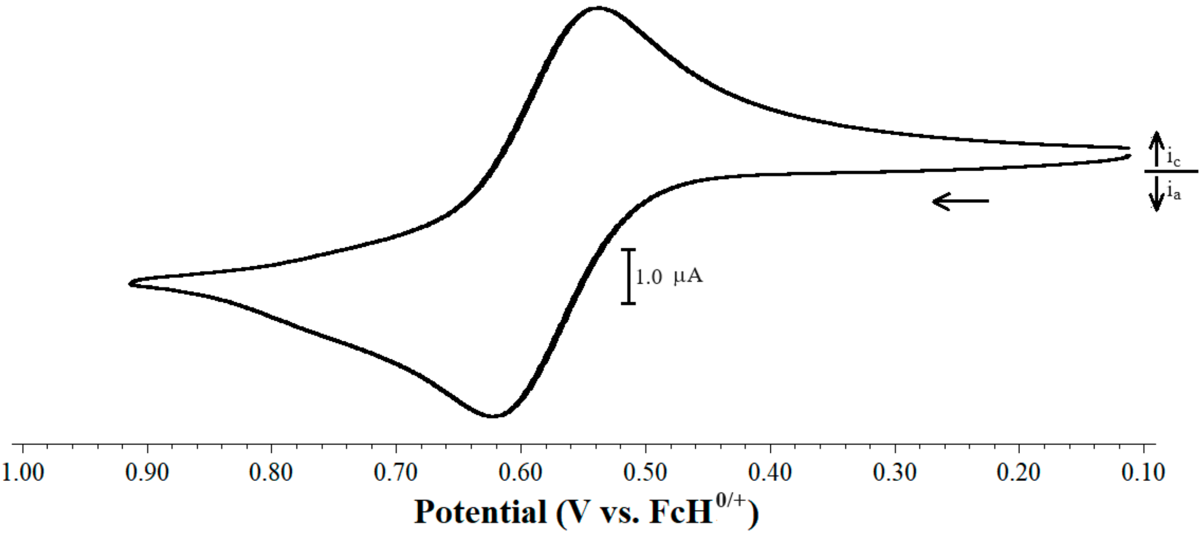

Fig. S35. CV scan of 1.0 mM [Pd(dppdtbpf)(PPh_3)Cl][$BArF_{24}$] with 0.1 M [NBu_4][PF_6] as the supporting electrolyte measured at 100 mV s^{-1}.

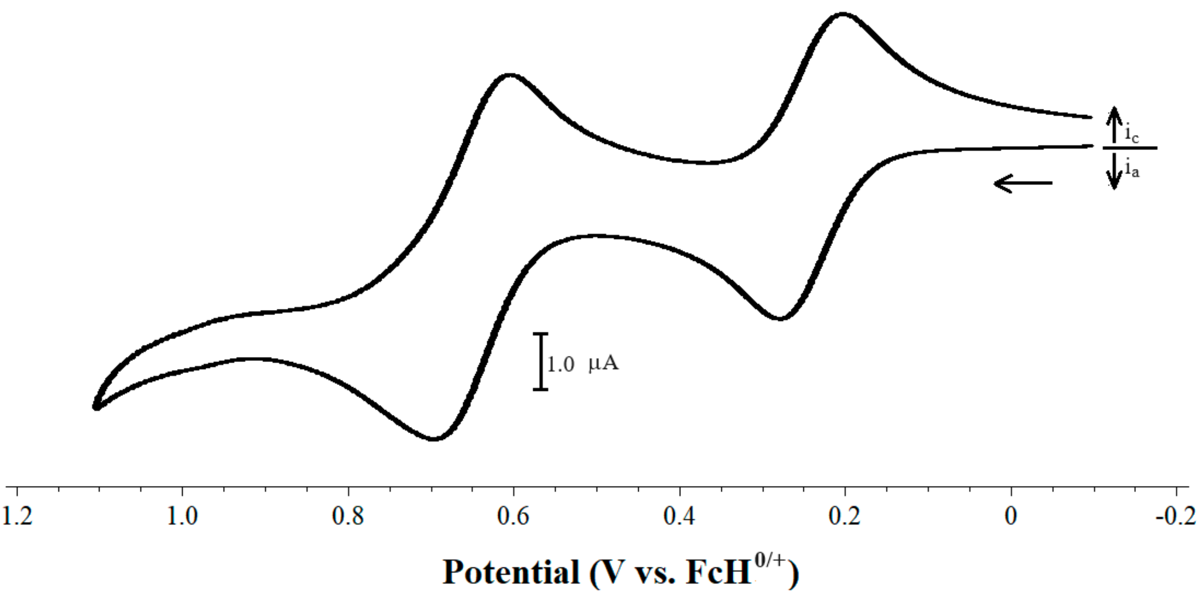

Fig. S36. CV scan of 1.0 mM [Pd(dppdtbpf)(PPh_2Fc)Cl][$BArF_{24}$] with 0.1 M [NBu_4][PF_6] as the supporting electrolyte measured at 100 mV s^{-1}.

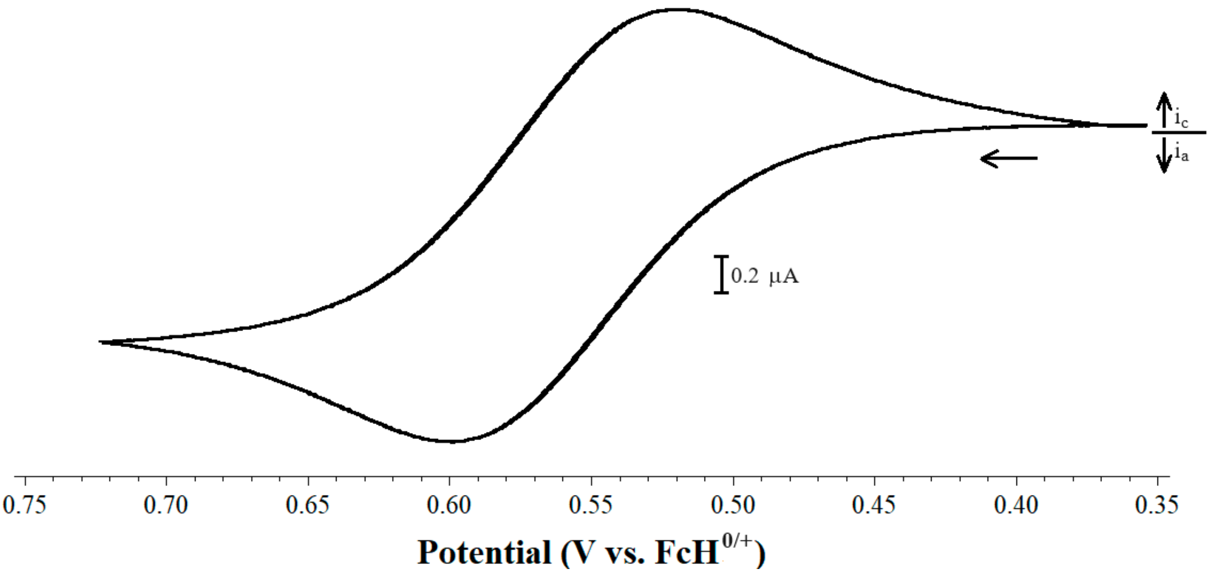

Fig. S37. CV scan of 1.0 mM [Pd(dcpf)(PMe_3)Cl][$BArF_{24}$] with 0.1 M [NBu_4][PF_6] as the supporting electrolyte measured at 100 mV s^{-1}.

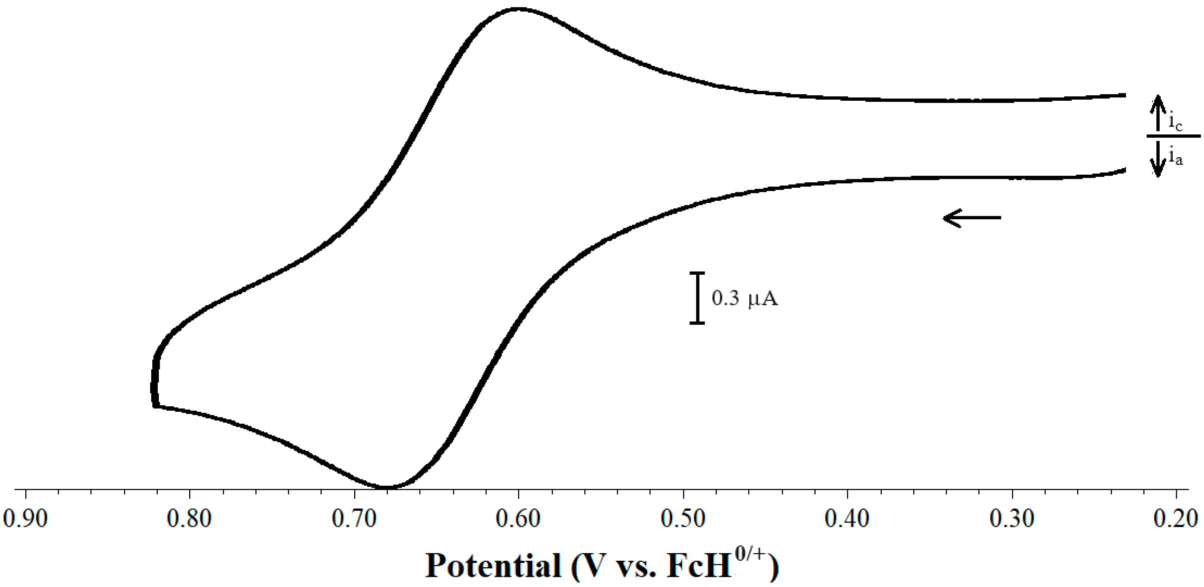

Fig. S38. CV scan of 1.0 mM [Pd(dfurpf)(PMe_3)Cl][$BArF_{24}$] with 0.1 M [NBu_4][PF_6] as the supporting electrolyte measured at 100 mV s^{-1}.

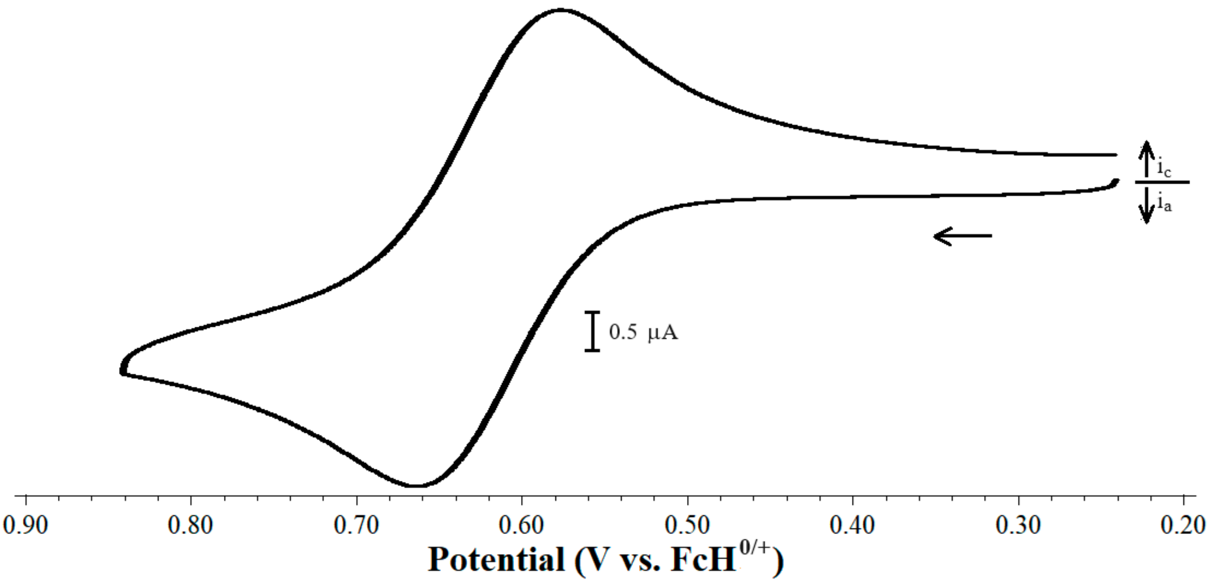

Fig. S39. CV scan of 1.0 mM [Pd(dfurpf)(PPh_3)Cl][$BArF_{24}$] with 0.1 M [NBu_4][PF_6] as the supporting electrolyte measured at 100 mV s^{-1}.

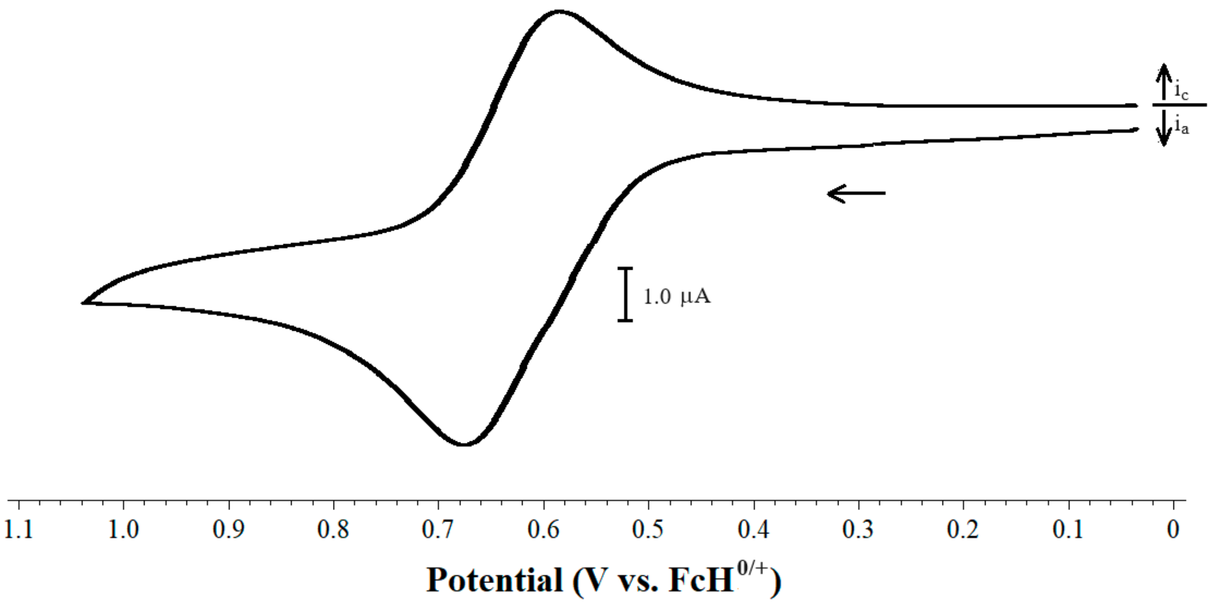

Fig. S40. CV scan of 1.0 mM [Pd(dppf)(P(NMe_2)$_3$)Cl][$BArF_{24}$] with 0.1 M [NBu_4][PF_6] as the supporting electrolyte measured at 100 mV s^{-1}.

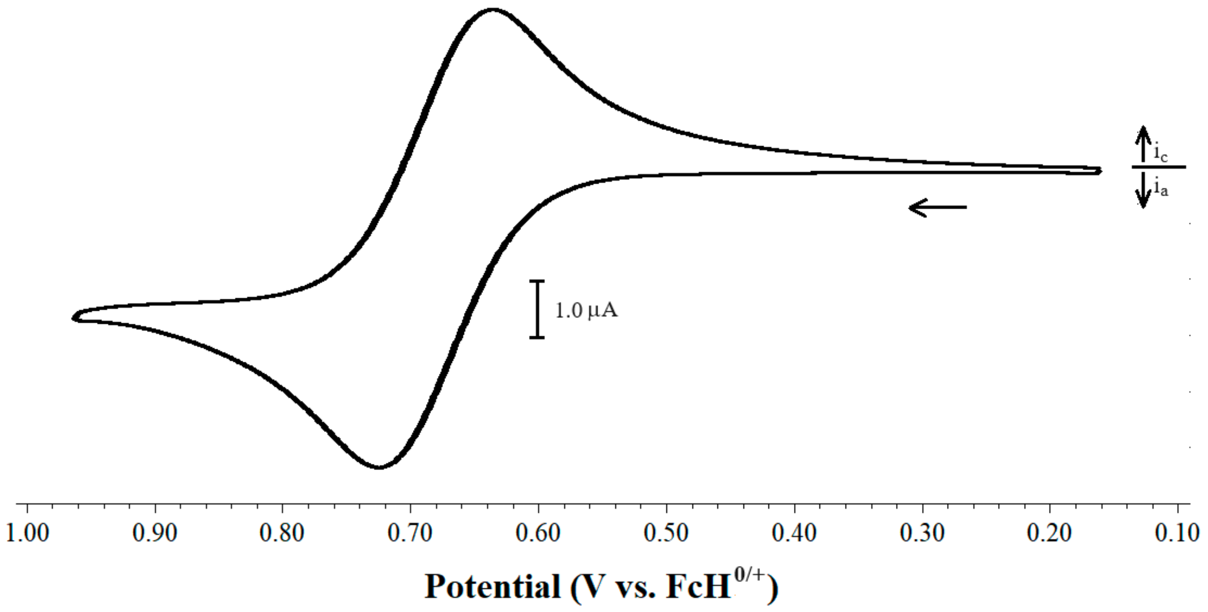

Fig. S41. CV scan of 1.0 mM [Pd(dppf)(P($CH_2Ph)_3$)Cl][$BArF_{24}$] with 0.1 M [NBu_4][PF_6] as the supporting electrolyte measured at 100 mV s^{-1}.

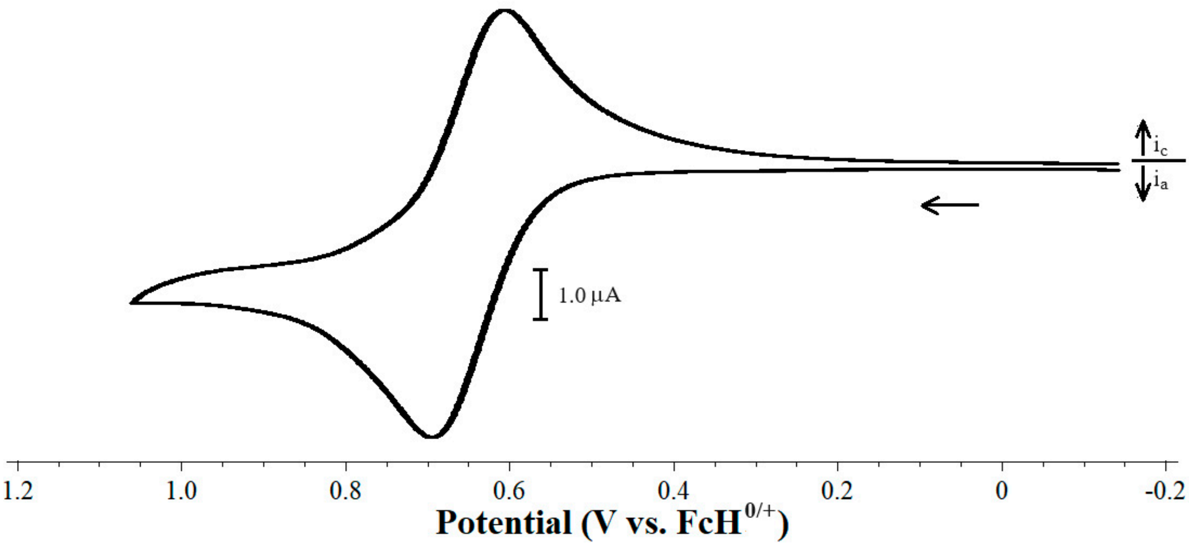

Fig. S42. CV scan of 1.0 mM [Pd(dppf)(P(*m*-tol)$_3$)Cl][BArF$_{24}$] with 0.1 M [NBu$_4$][PF$_6$] as the supporting electrolyte measured at 100 mV s^{-1}.

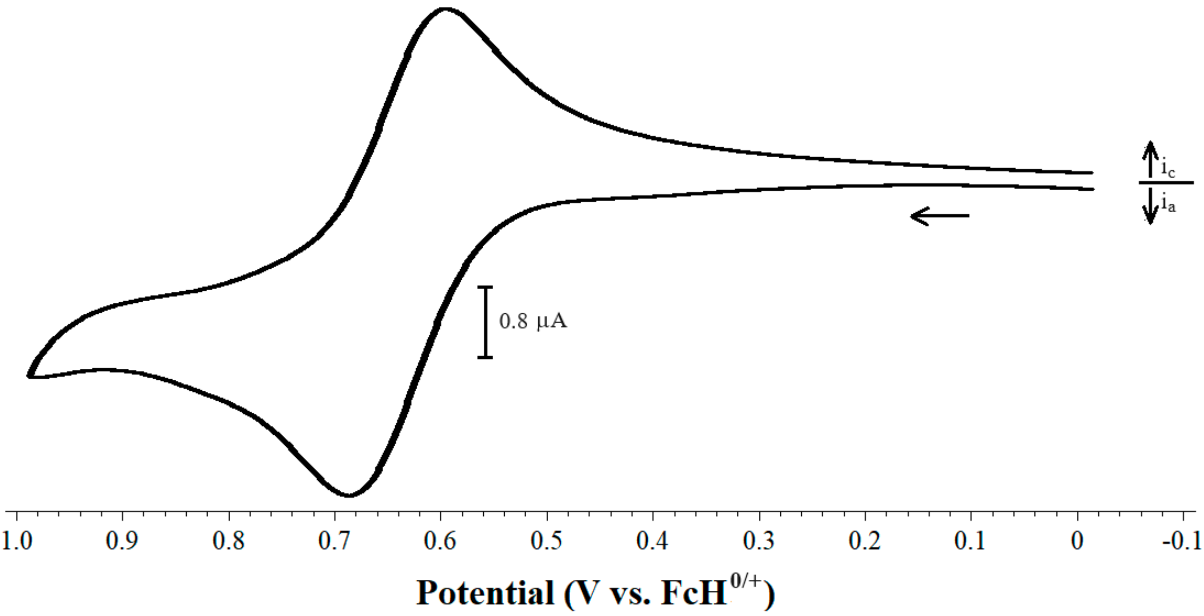

Fig. S43. CV scan of 1.0 mM [Pd(dppf)(P(*p*-tol)$_3$)Cl][BArF$_{24}$] with 0.1 M [NBu_4][PF_6] as the supporting electrolyte measured at 100 mV s^{-1}.

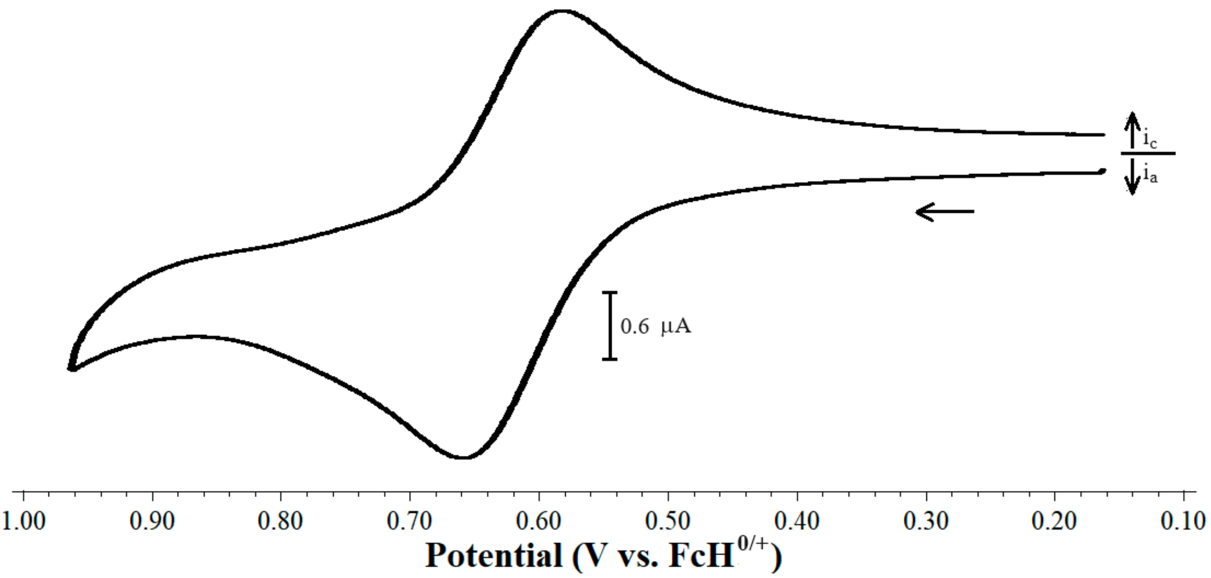

Fig. S44. CV scan of 1.0 mM [Pd(dppf)(P(*p*-$C_6H_4OMe)_3$)Cl][$BArF_{24}$] with 0.1 M [NBu_4][PF_6] as the supporting electrolyte measured at 100 mV s^{-1}.

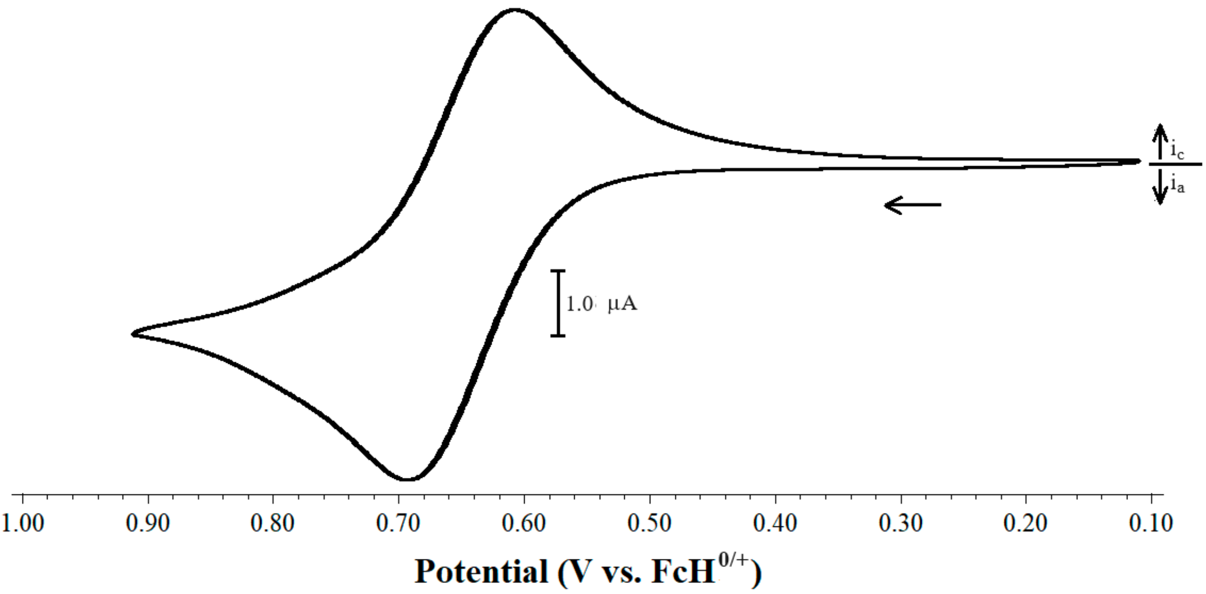

Fig. S45. CV scan of 1.0 mM [Pd(dppf)(P(*p*-C_6H_4F)$_3$)Cl][$BArF_{24}$] with 0.1 M [NBu_4][PF_6] as the supporting electrolyte measured at 100 mV s^{-1}.

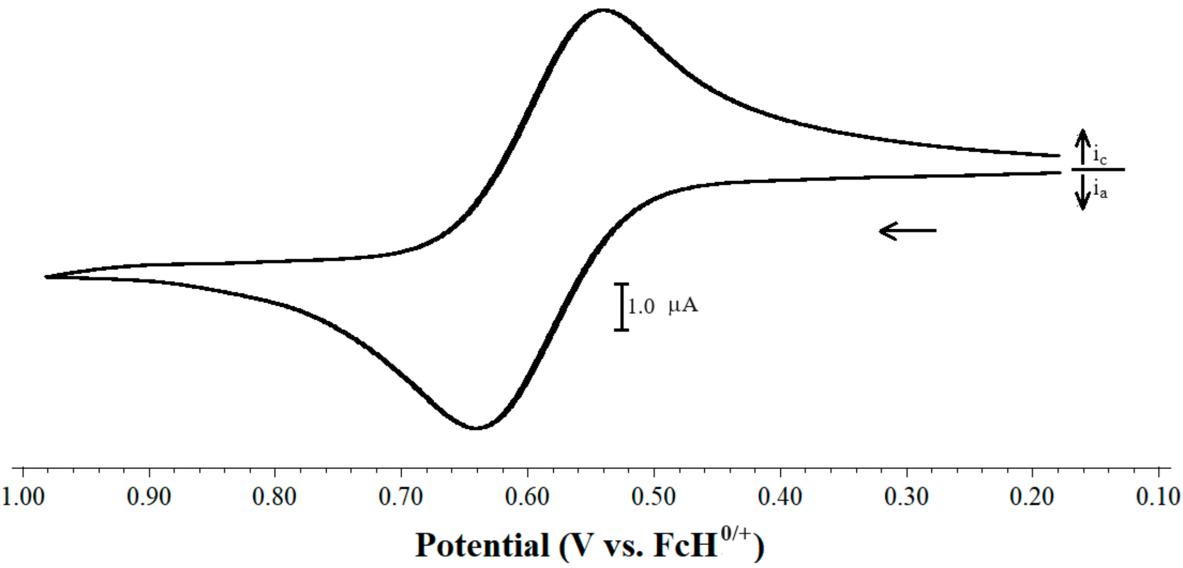

Fig. S46. CV scan of 1.0 mM [Pd(dfurpf)(P(*p*-$C_6H_4CF_3$)$_3$)Cl][BArF$_{24}$] with 0.1 M [NBu_4][PF_6] as the supporting electrolyte measured at 100 mV s^{-1}.
